# MicroRNAome Profile of *Euphorbia kansui* in Response to Methyl Jasmonate

**DOI:** 10.3390/ijms20061267

**Published:** 2019-03-13

**Authors:** Peng Li, Zheni Tian, Qing Zhang, Yue Zhang, Meng Wang, Xiaoai Fang, Wenjing Shi, Xia Cai

**Affiliations:** 1 Key Laboratory of Resource Biology and Biotechnology in Western China, Ministry of Education, Northwest University, Xi’an 710069, China; dangjiayuan2007@163.com (P.L.); tzn2734914487@163.com (Z.T.); 15771785620@163.com (Q.Z.); zy729225025@163.com (Y.Z.); wangmengss501@163.com (M.W.); 17791713237@163.com (X.F.); 2 Shaanxi Pharmaceutical Holding Group Co., Ltd., Xi’an 710069, China; shiwenjing82@163.com

**Keywords:** *Euphorbia kansui*, methyl jasmonate, microRNA

## Abstract

miRNAs play vital regulatory roles in different plant developmental stages and in plant response to biotic and abiotic stresses. However, information is limited on the miRNA regulatory mechanism to methyl jasmonate (MeJA). In this study, we used the microRNAome profile to illustrate the relevant regulatory mechanisms of *Euphorbia kansui* in response to methyl jasmonate (MeJA) through Illumina RNA-Seq. As a result, we identified 875 miRNAs corresponding to 11,277 target mRNAs, among them, 168 known miRNA families representing 6019 target mRNAs sequences were obtained. 452 miRNA-mRNA pairs presented an anti-correlationship (Cor < −0.50 and *p*-value of correlation ≤ 0.05). The miRNA with a fold change ≥ 2 and a *p* (*p*-Value) < 0.05 in pairwise comparison were identified as significant differentially expressed miRNAs (DEMs). The DEMs in MeJA treatment of 0, 24, 36 and 48 h were compared by using Short Time Expression Miner (STEM) cluster and 4 significant gene profiles (*p*-value ≤ 0.02) were identified. Through the kyoto encyclopedia of genes and genomes (KEGG) pathway and gene ontology (GO) enrichment analysis on all miRNA targets, we identified 33 mRNAs in terpenoid biosynthesis, which were regulated by miRNAs under MeJA treatment, so the miRNA maybe involved in the response of *E. kansui* plant to exogenous MeJA and the results would provide very useful information on illustrating the regulatory mechanism of *E. kansui* and also provide an overall view of the miRNAs response to MeJA stress of a non-model plant.

## 1. Introduction

*Euphorbia kansui* Liou is a classical traditional Chinese medicine (TCM), which dried root has long been used to treat asthma, edema, ascites [1]. There are many secondary metabolites in *E. kansui*, diterpense and triterpenes are the main compounds, which show anti-virus, skin irritating and modulation of multidrug resistance effects [2,3,4]. The extracts in folk are widely used in anti-tumor, anti-cancer, anti-fertility, anti-virus, anti-inflammatory activity, regulating the immune system and other activities [5,6,7,8].

Jasmonic acid (JA) and its derivatives referred to as jasmonates, are key signaling molecules and play roles in many biological processes, such as growth inhibition, senescence, wound response, plant defense and the secondary mechanism [9,10]. The JA system is an important component in complex plant hormone signaling systems [11], which can elicit three major classes of plant secondary metabolites, terpenoids, alkaloids and phenyipropanoids [9,12]. Methyl jasmonate (MeJA) is the most important kind of JA system, which has also been used to elicit defense responses in many species through enhancing the secondary metabolites production [13,14,15,16], such as volatile terpenoids from *Amomum villosum* [17], triterpene in *Euphorbia pekinensis* [18] and tropane alkaloids in *Hyoscyamus niger* [19].

MicroRNAs (miRNAs) are short non-coding RNAs with a length of 19–25 nucleotides. Many studies have demonstrated that miRNAs play crucial roles in a wide range of biological processes and stress responses [20,21,22,23]. In plant, miRNAs are post-transcriptional regulators of gene expression, which are related to growth, development and stress, and in different stages of plant development the miRNAs expression differs [24,25,26,27]. Biswas et al. [28] reported that miRNAs play an important role in different processes in a post-transcriptional manner by down-regulating target gene products. Shriram et al. [29] also reported that when plants are stressed, miRNAs downregulated their target mRNAs and acculated the accumulation and function of positive regulators. Some studies have found that miRNAs are involved in the drought stress of wheat [30,31] or dehydration stress in *Triticeae* [32]. A large-scale multiomics analysis showed that miRNAs involved the potential regulatory in wheat stem sawfly [33]. Shen et al. reported that some miRNAs of *Oryza sativa* responded to a drought, salt and the cold [34].

At present, miRNA have attracted more and more attention, and in different plants where they are more conserved and novel miRNAs have been identified [35,36]. Some miRNAs were regulated to respond to stress conditions, including MeJA, salicylic acid (SA), abscisic acid (ABA), melatonin and rice stripe virus (RSV), which suggests that miRNAs direct post-transcriptional regulation of their respective target genes to replay the stress [34,37,38,39,40].

Clarifying the regulatory relationships between miRNAs and their corresponding mRNA targets, many biological questions have been further understood [40,41,42]. Expression level analysis of miRNAs is particularly important in exploring their biological functions. Using Illumina RNA-Seq, a second-generation sequencing-based technology, it is possible to measure miRNA expression levels in tissues of interest. In this study, we used Illumina RNA-Seq to examine the microRNA transcriptome of the control group and MeJA treatment groups at different handling times of *Euphorbia kansui*. Through analysis, the microRNAome profile was used to illustrate the relevant regulatory mechanisms of *E. kansui* in response to MeJA. The results showed that miRNAs involved in the response of *E. kansui* plant to exogenous MeJA, which will provide very useful information on illustrating the regulatory mechanism of *E. kansui* and also provide an overall view of miRNAs response to MeJA stress of a non-model plant.

## 2. Results

### 2.1. Sequencing of miRNA Library and Identification of miRNA Families

**Figure 1:**
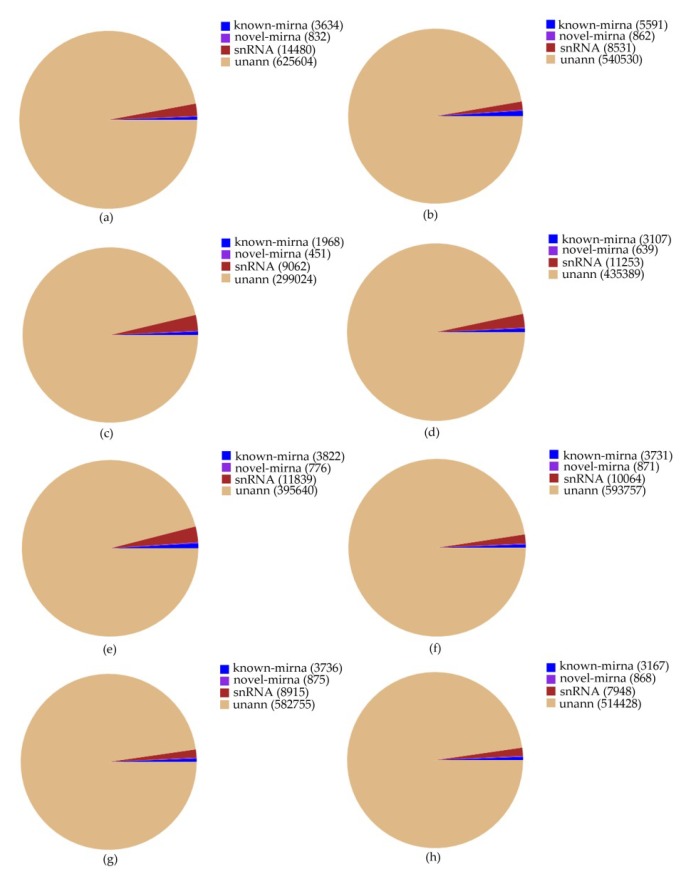
Tag quantity and abundance statistics of different categories in eight small RNA libraries. CK, T1, T2 and T3 represent 0 h, 24 h, 36 h and 48 h MeJA treatment respectively and (**a**–**h**) represents CK-1, CK-2, T1-1, T1-2, T2-1, T2-2, T3-1, T3-2 respectively (two biological replicates for each treatment, named CK-1, CK-2, etc.).

In order to examine whether miRNAs are involved in the MeJA response in *Euphorbia kansui*, we performed high-throughput sequencing analysis of miRNA in leaves treated with MeJA 0 (CK), 24 (T1), 36 (T2) and 48 (T3) hours (two biological replicates for each treatment, named CK1, CK2, T1-1, T1-2, T2-1, T2-2 and T3-1 T3-2, in turn). Eight small RNA libraries were constructed independently. 14,388,846, 12,351,316, 12,031,311, 14,334,780, 14,756,498, 14,955,951, 15,196,075 and 12,307,706 raw reads were generated from MeJA treatment of eight small RNA libraries, respectively. After adaptor, insert and poly (A) contaminations and low quality reads (smaller than 18 nt) were removed, 10,293,875, 7,715,305, 9,866,402, 11,727,057, 6,583,270, 10,268,043 and 10,094,894, 8,794,507 clean reads were obtained. 186,344, 147,068, 89,160, 128,308, 130,317, 166,994 and 156,566, 143,462 unique reads were mapped to our transcriptome dataset, respectively. The mapped small RNA sequences were clustered into known miRNAs, novel miRNAs, small nuclear RNA (snRNA) and an uncharacterized group (Table S1, Figure 1). The sequence of precursors and the secondary structures of the novel miRNAs were listed in Table S2.

### 2.2. Differentially Expressed miRNAs (DEMs) Respond to Methyl Jasmonate (MeJA) Treatment

**Figure 2:**
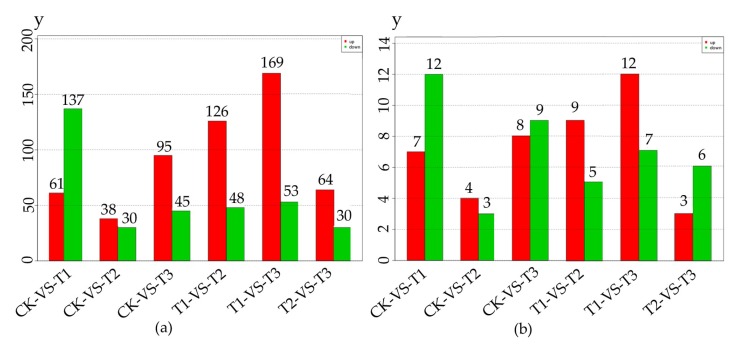
The number of differentially expressed miRNA in 4 methyl jasmonate (MeJA) treatment groups. The numbers on the column showed the quantity of upregulated (red) and downregulated (green) miRNA. (**a**) The number of differentially expressed total miRNA; (**b**) The number of differentially known miRNA. CK, T1, T2 and T3 represented 0 h, 24 h, 36 h and 48 h MeJA treatments respectively. The *y*-axis represents the number of miRNA.

We analyzed differentially expressed miRNAs (DEMs) to study the regulation of miRNA in MeJA treatment. From Figure 2a,b, we could obtain the number of differentially expressed total miRNA and known miRNA respectively.

**Figure 3:**
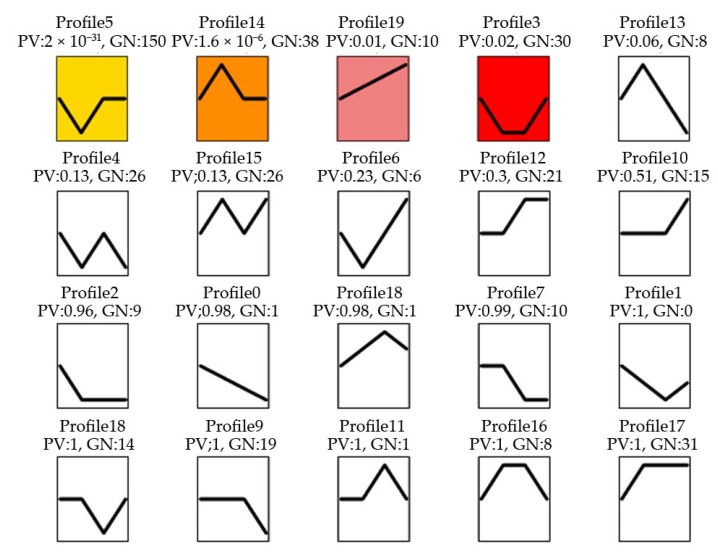
miRNA expression trend profiles among four MeJA treatment groups. PV: *p*-value; GN: the number of miRNA. The colour square frame denotes significant profiles (*p*-value ≤ 0.02). Each graph displays the mean pattern of expression (black lines) of the profile miRNA. The *x*-axis represents 0 h, 24 h, 36 h and 48 h MeJA treatment and the *y*-axis represents the log2 fold change of miRNA expression.

The expression patterns of these DEMs in response to MeJA treatment of 0, 24, 36 and 48 h were compared by using a Short Time Expression Miner (STEM) cluster of 4 significant gene profiles (*p*-value ≤ 0.02): STEM profile 5 (150 genes), profile 14 (38 genes), profile 19 (10 genes) and profile 3 (30 genes) were identified (Figure 3). It could be found that in profile 5 and 3, after MeJA treatment 24 h, the miRNA expression levels decreased to the lowest. While in profile 14, miRNA expression levels increased to the highest at MeJA treatment 24 h, and in profile 19, miRNA expression levels increased directly to the highest at MeJA treatment 48 h.

### 2.3. Target RNA Prediction and Enrichment Analysis

In total, 875 conserved miRNAs, corresponding to 11,277 target RNA in 8 samples were identified; among them, 168 known miRNA families representing 6019 target RNA were obtained. The details of the target RNA are shown in Table S3. In known miRNA, the most abundant miRNA was the MIR169 family, accounting for 358 of the total miRNA. MIR156 (308), MIR166 (258), MIR171 (237) and MIR395 (232), family also had high abundance of expression (Table S4). In addition, we identified 707 novel miRNA, corresponding to 5258 RNA sequences.

**Figure 4:**
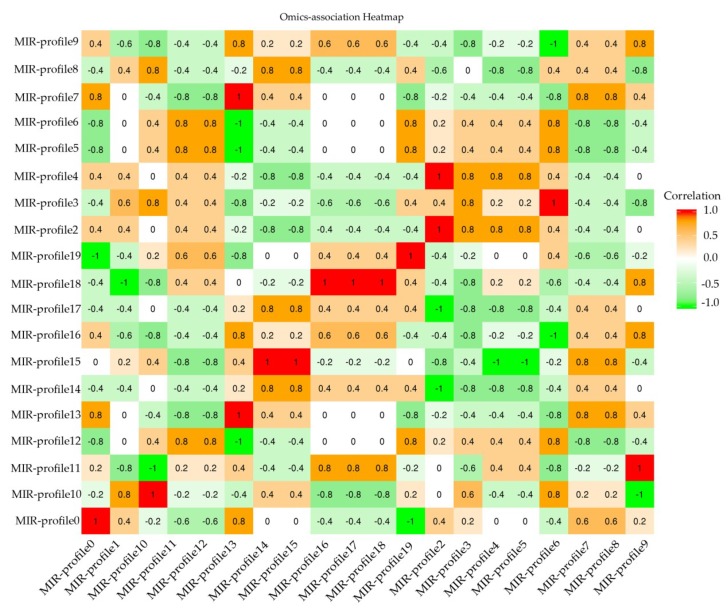
RNA-MIR profile association heatmap. Crosswises are RNA-profiles and lengthways are miRNA-profiles. Correlation < −0.50 is the negative RNA-MIR profile association in response to MeJA treatment.

In general, the target mRNAs were negatively regulated by their mature miRNA with either translational repression or mRNA degradation. According to correlation between miRNA-mRNA, an association of heatmap was built in Figure 4. 452 miRNA-mRNA pairs presented an anti-correlationship between the expression level of miRNAs and mRNAs (Cor < −0.50 and *p*-value of correlation ≤0.05) (Table S5).

**Figure 5:**
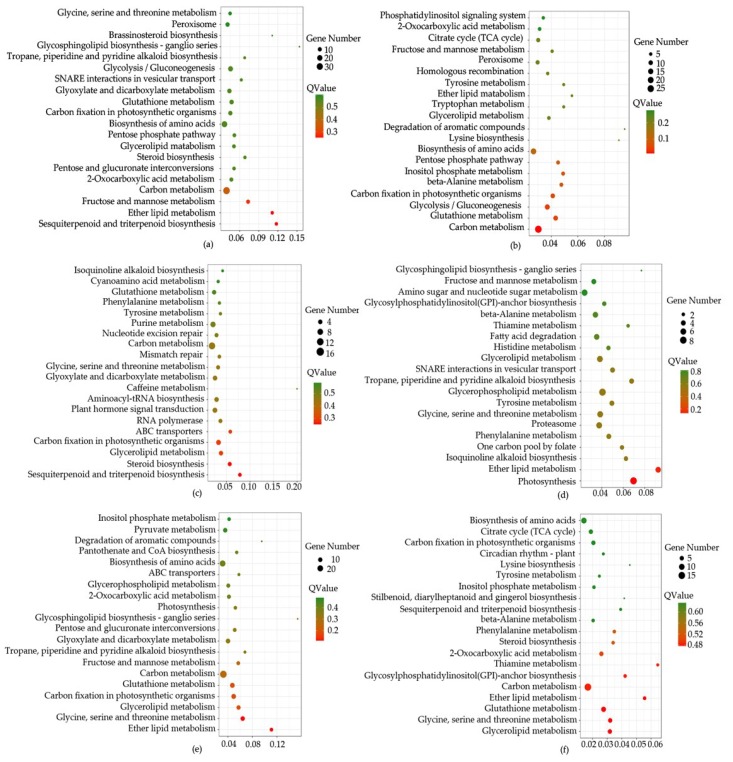
The top 20 of pathway enrichment using pairwise comparison. The size of the dots represent the number of genes. The color of the dot represents the Qvalue. CK, T1, T2 and T3 represent 0 h, 24 h, 36 h and 48 h MeJA treatment respectively. (**a**–**f**) is the description of CK-VS-T1, CK-VS-T1, CK-VS-T3, T1-VS-T2, T1-VS-T3 and T2-VS-T3 respectively. The *x*-axis represents rich factor. The *y*-axis represents the top 20 of pathway enrichment.

Based on all miRNA targets using kyoto encyclopedia of genes and genomes (KEGG) enrichment analysis, we found that some pathways were significantly enriched (*p* < 0.05). For example, in CK-VS-T1, 318 differentially expressed miRNAs (DEGs) were annotated in 112 pathways, “Sesquiterpenoid and triterpenoid biosynthesis” (6, 1.89%), “Ether lipid metabolism” (6, 1.89%), “Carbon metabolism” (35, 11.01%), “Steroid biosynthesis” (6, 1.89%), “Glutathione metabolism” (12, 3.77%) were significantly enriched. In CK-VS-T2, 174 DEGs were annotated in 94 pathways, such as “Carbon metabolism” (27, 15.52%), “Glutathione metabolism” (11, 6.32%), “Ether lipid metabolism” (3, 1.72%), “Steroid biosynthesis” (5, 1.15%), “Sesquiterpenoid and triterpenoid biosynthesis” (1, 0.57%). 156 DEGs (CK-VS-T3) were annotated in 95 pathways, such as “Sesquiterpenoid and triterpenoid biosynthesis” (4, 2.56%), “Steroid biosynthesis” (5, 3.21%), “Carbon metabolism” (7, 10.9%), “Glutathione metabolism” (6, 3.85%), “Ether lipid metabolism” (2, 1.28%). 218 DEGs (T1-VS-T2) were annotated in 101 pathways, such as “Ether lipid metabolism” (5, 2.29%), “Sesquiterpenoid and triterpenoid biosynthesis” (2, 0.92%), “Glutathione metabolism” (6, 2.75%), “Steroid biosynthesis” (1, 0.46%), “Carbon metabolism” (13, 5.96%). 262 DEGs (T1-VS-T3) were annotated in 111 pathways, such as “Ether lipid metabolism” (6, 2.29%), “Glutathione metabolism” (9, 3.44%), “Carbon metabolism” (29, 11.07%), “Sesquiterpenoid and triterpenoid biosynthesis” (3, 1.15%), “Steroid biosynthesis” (3, 1.15%). 125 DEGs (T2-VS-T3) were annotated in 85 pathways, such as “Glutathione metabolism” (7, 5.6%), “Ether lipid metabolism” (3, 2.4%), “Carbon metabolism” (15, 12%), “Steroid biosynthesis” (3, 2.4%), “Sesquiterpenoid and triterpenoid biosynthesis” (2, 1.6%), (Figure 5, Table S6).

Gene Ontology (GO) enrichment of all miRNA target DEGs were investigated to functionally classify the genes affected by MeJA. From Table S7, we found that catalytic activity, metabolic process and cellular process were identified as the top three terms by pairwise comparison. 417 DEGs (CK-VS-T1) in the biological process were enriched in 698 GO terms, including 292 DEGs in the cellular process and 298 DEGs in the metabolic process. 180 DEGs (CK-VS-T2) in the biological process were enriched in 452 GO terms, including 112 DEGs in the cellular process and 130 DEGs in the metabolic process. 215 DEGs (CK-VS-T3) in the biological process were enriched in 542 GO terms, including 146 DEGs in cellular process and 160 DEGs in metabolic process. 363 DEGs (T1-VS-T2) in biological process were enriched in 699 GO terms, including 256 DEGs in the cellular process and 249 DEGs in the metabolic process. 273 DEGs (T1-VS-T3) in the biological process were enriched in 687 GO terms, including 272 DEGs in the cellular process and 267 DEGs in the metabolic process. 137 DEGs (T2-VS-T3) in the biological process were enriched in 396 GO terms, including 91 DEGs in the cellular process and 91 DEGs in the metabolic process.

### 2.4. Differentially Expressed Genes (DEGs) and DEMs in Terpenoid Biosynthesis Associated with MeJA Treatment

Kyoto encyclopedia of genes and genomes (KEGG) is a database about genomic information. Due to the lack of an available genome sequence for *Euphorbia kansui*, 318, 174, 156, 218, 262 and 125 DEGs in CK-VS-T1, CK-VS-T2, CK-VS-T3, T1-VS-T2, T1-VS-T2 and T2-VS-T3 with KEGG pathway annotation respectively. In addition, in CK-VS-T1, 6 (1.89%) DEGs were detected in sesquiterpenoid and triterpenoid biosynthesis pathway, 6 (1.89%) in steroid biosynthesis pathway, 2 (0.63%) DEGs in terpenoid backbone biosynthesis pathway and 3 (0.94%) DEGs in ubiquinone and other terpenoid-quinone biosynthesis pathway. In CK-VS-T2, 2 (1.15%) DEGs were detected in steroid biosynthesis pathway, 1 (0.57%) DEGs in terpenoid backbone biosynthesis pathway and 2 (1.15%) DEGs in ubiquinone and other terpenoid-quinone biosynthesis pathway. In CK-VS-T3, 4 (2.56%) were detected in sequiterpenoid and triterpenoid biosynthesis pathways, 5 (3.21%) in steroid biosynthesis pathway, 1 (0.64%) DEGs in terpenoid backbone biosynthesis pathway and 1 (0.64%) DEGs in ubiquinone and the other terpenoid-quinone biosynthesis pathway. In T1-VS-T2, 2 (0.92%) DEGs were detected in sequiterpenoid and triterpenoid biosynthesis pathways, 2 (0.92%) in the steroid biosynthesis pathway, 1 (0.64%) in the steroid biosynthesis pathway, 4 (1.83%) in the terpenoid backbone biosynthesis pathway, 1 (0.46%) DEG in the ubiquinone and other terpenoid-quinone biosynthesis pathway. In T1-VS-T3, 3 (1.15%) DEMs were detected in sesquiterpenoid and triterpenoid biosynthesis, 3 (1.15%) DEGs in the steroid biosynthesis pathway, 4 (1. 53%) DEGs in terpenoid backbone biosynthesis pathway and 2 (0.76%) DEGs in the ubiquinone and other terpenoid-quinone biosynthesis pathway. In T2-VS-T3, 2 (2.16%) DEGs were detected in sesquiterpenoid and triterpenoid biosynthesis, 1 (0.8%) in terpenoid backbone biosynthesis pathway and 2 (1.6%) DEGs in ubiquinone and the other terpenoid-quinone biosynthesis pathway (Table 1). The miRNAs, which target these DEGs are displayed in Table S5. Moreover, we identified 33 mRNAs in terpenoid biosynthesis, which were regulated by miRNAs under the MeJA treatment (Table S8)

### 2.5. Verification of DEM Profiles Associated with MeJA Treatment with Real-time Quantitative PCR Detecting System (QPCR)

**Figure 6:**
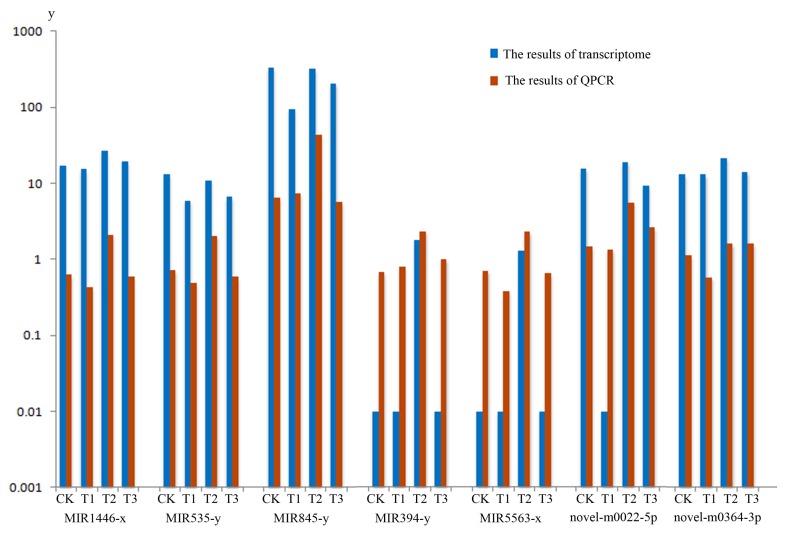
Real-time Quantitative PCR Detecting System (QPCR) verification and transcriptome of the miRNA. The blue column shows the data of miRNA transcriptome. The orange column shows the results of QPCR verification. CK, T1, T2 and T3 represent 0 h, 24 h, 36 h and 48 h MeJA treatment, respectively. The *y*-axis represents the relative expression of genes of transcriptom and QPCR.

In order to experimentally verify the DEMs profile obtained by sequence data associated with MeJA treatment, Real-time Quantitative PCR Detecting System (QPCR) analysis was conducted respectively. A total of seven miRNA in the terpenoid biosynthesis pathway, including MIR1446-x, MIR535-y, MIR845-y, MIR394-y, MIR5563-x, novel-m0022-5p, novel-m0346-3p were selected to analyze (two biological repeats per miRNA), the QPCR verification results of seven miRNA in the terpenoid biosynthesis calculation as shown in Table S9 and miRNA expression patterns can be seen from Figure 6. The results suggested that the expression levels of the miRNAs verified were mostly consistent with those transcriptome data (Table S8). Overall, QPCR verification analysis confirmed the expression profiles detected by DEMs analysis from transcriptome.

## 3. Discussion

### 3.1. miRNA Involved in the Response of Euphorbia kansui Plant to Exogenous MeJA

The methyl jasmonate (MeJA) has been identified as a vital cellular regulator that mediates the secondary metabolism containing biosynthesis of flavonoids, alkaloids and terpenoids and so on. Many studies have showed that miRNAs are involved the potential regulatory when plants are stressed by drought, dehydration, salt and cold [30,31,32,33,34]. In the present study, in order to find MeJA-mediated miRNAs, we used the *E. kansui* leaves after MeJA mock-treatment 24, 36 and 48 h for miRNA analysis to profile their transcriptional alterations in response to MeJA elicitation. 875 conserved miRNAs corresponding to 11,277 target RNAs were identified, which contained 168 known miRNAs and 707 novel miRNAs. Among miRNAs identified, differentially expressed miRNAs in response to MeJA were obtained. From expressed profiles and the correlation between DEGs and DEMs, 452 miRNA-mRNA pairs presented an anti-correlationship between the expression level of miRNAs and mRNAs were found. As a result, miRNA maybe involved in the response of the *E. kansui* plant to exogenous MeJA, and the expression patterns of miRNA regulate various metabolic pathways.

### 3.2. miRNA Involved in the Regulation of Terpenoid Biosynthesis after Exogenous MeJA Treatment in Euphorbia kansui

MicroRNAs (miRNAs) are endogenous and short non-coding small RNAs, which participate in biological and metabolic processes in plants through negatively regulating their target mRNA. The negative interaction between the miRNA and its target mRNA regulates its expression through mRNA cleavage, translational repression, or epigenetic modifications [43,44].

Due to the lack of an available genome sequence for *E. kansui*, we found 424 DEMs with KEGG pathway annotation. The pathways involved in terpenoid biosynthesis including terpenoid backbone biosynthesis pathway, sesquiterpenoid and triterpenoid biosynthesis pathway, steroid biosynthesis pathway, ubiquinone and other terpenoid-quinone biosynthesis pathway, and diterpenoid biosynthesis pathway were annotated in different comparisons of control and three MeJA treatments by KEGG analysis (Table 1).

We identified 33 mRNAs in terpenoid biosynthesis, which were regulated by miRNAs under MeJA treatment (Table S8). At present, information on miRNA involvement in the biosynthesis of the secondary metabolites in plants is limited. Some reports were focused on the involvement of some special miRNA in the regulation of a plant’s secondary metabolism [45,46,47,48,49]. Shen et al. [50] found that auxin (indole acetic acid, IAA) repressed the expression of key terpenoid indole alkaloid pathway genes in *Catharanthus roseus* seedlings. Hazra et al. [51] reported that some non-responsive genes of reactive oxygen species were controlled by MeJA through the down regulation of five secondary metabolite biosynthesis specific miRNAs. From our results, we found the high abundance of expression was focused on MIR156, MIR166, MIR169, MIR171 and MIR395 family. It was previously reported that MIR156, MIR166, MIR169, MIR171 were all connected with the development of seedling. MIR395 family participated in the development of Euphorbiaceae and might be involved in metabolic processes [52]. These MIR families were up-regulated or down-regulated under the inducing of cold, drought, tension, compression, dehydration, salinity, treatment of phytohormone abscisic acid and ultraviolet-B (UV-B) radiation and other abiotic and biotic stresses [53,54,55,56,57]. In the present studies, we found some miRNAs in a different pathway of terpenoid biosynthesis, which were induced by MeJA (Table S8). We selected seven miRNA’s in the terpenoid biosynthesis pathway to verify the QPCR analysis. From Figure 6, we observed the verification results of seven miRNAs, which were mostly in agreement with the miRNA-seq data.

MIR1446-x targets unigene063000 coding PRPOL, which takes part in ubiquinone and other terpenoid-quinone biosynthesis, MIR845-y targets unigene014530 coding DHCR24, which is an important enzyme in steroid biosynthesis, novel-m0022-5p targets Unigene003418 coding MHGR, which is an important enzyme in terpenoid backbone biosynthesis, MIR394-y targets unigene069994 coding VTE4, which is an important enzyme in ubiquinone and other terpenoid-quinone biosynthesis, MIR5563-x targets unigene069994 which is a sesquiterpene synthase) (Table S10). All these indicated that these DEMs were involved in the regulation of terpenoid biosynthesis by their target mRNA at an early stage of MeJA treatment.

### 3.3. miRNA Involved in Other Pathways

From the miRNA targets KEGG enrichment analysis, we found that some miRNA involved in other pathways, such as “Glutathione metabolism” and “Ether lipid metabolism” (Table S10) were affected by MeJA obviously.

Glutathione (GSH) is a low molecular weight tripeptide, which is playing a vital role in metabolism and cell function, e.g., providing homeostasis, involvement in the ascorbate–glutathione cycle and the detoxification of xenobiotics, and GSH is also important to detoxify the exogenous substances, isolate heavy metal and take part in other stress [58]. In our study, we found Unigene028077, which was predicted to be glutathione reductase, cytosolic-like (GR), was an important component of the antioxidant machinery to respond to abiotic stress in plant and also play an important role in tolerance to abiotic stress [59]. GR was regulated by novel-m0076-3p; novel-m0247-5, novel-m0415-3p, novel-m0438-5p, novel-m0439-5p and novel-m0639-5p.

Miao et al. reported that miRNA-34a target genes were enriched in many pathways, including ether lipid metabolism, alpha-linolenic and so on [60]. From the KEGG enrichment, we found that the miRNA6483-y and novel-0375-3p target Unigene027979 in ether lipid metabolism were affected by MeJA. Moreover, Unigene027979 was predicted to be choline/ethanol aminephosphotransferase 1 (Jatropha curcas), which is the key enzyme in the final step of the synthesis of PtdCho (phosphatidylcholine) via the Kennedy pathway.

## 4. Materials and Methods

### 4.1. Plant Material and MeJA Treatment

Healthy *Euphorbia kansui* plants were grown at the Botanical Garden of Northwest University in Shaanxi Province (Shaanxi, China).

The 2 to 3 year old plants at the same development stage were assigned to four groups (Two biological repeats per group), sprayed with 20 μM MeJA (in Milli-Q water). The MeJA solution was sprayed as a fine mist to completely wet the adaxial side of each leaves. Leaves from the group treated with MeJA 24 (T1), 36 (T2) and 48 (T3) h were collected separately and directly into liquid nitrogen. All materials were stored at −80 °C until analysis.

### 4.2. MicroRNA Sequencing

Total RNA from four groups (two biological repeats per group) was separately isolated from leaves using RNAprep Pure Plant Kit (TIANGEN, DP441, Beijing, China), according to the manufacturer’s instructions. The RNA molecules in a size range of 18–30 nt were separated and purified by polyacrylamide gel electrophoresis (PAGE). The 5′ adapters were ligated to the RNAs as well, then the 3′ adapters were added and the 36–44 nt RNAs were enriched. The ligation products were reverse transcripted by PCR amplification and the 140–160 bp size PCR products were enriched to generate a cDNA library and sequenced using Illumina HiSeqSE50 by Sagene Biotech Co., Ltd. (Guangzhou, China). *E. kansui* leaves of four treatment groups (two biological replicates for each treatment) were sequenced and have been deposited into the NCBI (National Center for Biotechnology Information) Sequence Read Archive database (accession number: SRP126443).

### 4.3. Sequencing Data Processing and Analysis

Reads obtained from the sequencing machines included dirty reads containing adapters or low quality bases which would have affected the following assembly and analysis. To get clean tags, raw reads were further filtered through after the low quality reads were removed, which contained more than one low quality (*Q*-value ≤ 20) base or contained the unknown nucleotides (N), reads without 3′ adapters, reads containing 5′ adapters, and then removed the reads containing 3′ and 5′ adapters but no small RNA fragment between them. From the reads containing polyA in small RNA fragments and reads shorter than 18 nt (not include adapters), we obtained the clean tags.

All of the clean tags were aligned with small RNAs in the GeneBank database (Release 209.0), Rfam database (11.0) and reference genome to identify and remove rRNA, scRNA, snoRNA, snRNA and tRNA. The tags that mapped to exons or introns might have been fragments from mRNA degradation and the tags that mapped to repeat sequences, all of these tags were removed.

All of the clean tags were then searched against the miRBase database (Release 21) to identify known miRNAs of *E. kansui*, then those species the miRNAs alignment with other species was a dependable way to identify the known miRNAs. All of the unannotated tags were aligned with reference transcriptome. According to their transcriptome positions and hairpin structures predicted by software Mireap_v0.2, the novel miRNA candidates were identified.

### 4.4. miRNA Expression Profiles

Total miRNA consists of existing miRNAs, miRNA, known miRNA and novel miRNA, miRNA expression level was calculated based on their expression in each sample and normalized to transcripts per million (TPM) according to the following formula.

TPM = Actual miRNA counts/Total counts of clean tags × 106

In addition, the expression of existing miRNA, known miRNA and novel miRNA was also analyzed individually. Meantime, the analysis of miRNA families was used to identify if the miRNAs existed in other species. The analysis result was marked as “+” or “−” which referred to existing or non-existing, respectively.

The mRNA-sequence on the four groups described in 4.1 (two biological repeats per group) were also analyzed using Illumina HiSeqTM 4000 (Gene Denovo Biotechnology Co., Guangzhou, China) and the mRNA transcriptome of *Euphorbia kansui* in response to MeJA were obtained. The sequences have been deposited into the NCBI Sequence Read Archive database (Accession number: SRP126436).

The correlation analysis between miRNA-mRNA profiles were performed using the method of correlation coefficient. Correlation < −0.50 is the negative RNA-MIR profile association in response to MeJA treatment.

### 4.5. Differentially Expressed miRNAs Analysis and Target Prediction

Differentially expressed miRNAs (DEMs) between the control and three treatments were identified with a fold change ≥ 2 and *p*-value < 0.05 in a comparison as significant DEMs. The calculation formula was as follows. Based on the sequences of the known miRNAs and novel miRNAs, the candidate target genes were predicted using patmatch softwares (v1.2, https://www.arabidopsis.org/cgi-bin/patmatch/nph-patmatch.pl).

$$\begin{array}{l} p(x|y)={\left(\frac{N_{2}}{N_{1}}\right)}^{y}\frac{\left(x+y\right)!}{x!y!{\left(1+\frac{N_{2}}{N_{1}}\right)}^{\left(x+y+1\right)}}\\ C(y\leq y_{\min}|x)=\sum_{y=0}^{y\leq y_{\min}|}p(y|x)\\ D(y\geq y_{\max}|x)=\sum_{y\geq y_{\max}|}^{\omega }p(y|x) \end{array}$$

miRNA target genes function categorization and pathway enrichment were performed based on the GO and KEGG databases. DEGs were subjected to enrichment analysis of KEGG pathways. The calculation formula was as follows (*N* represents the number of all genes with pathway annotation, n represents the number of DEGs in *N*, *M* is the number of all genes that are annotated to the specific pathway terms; m represents the number of DEGs in *M*). This analysis could recognize the most biochemical metabolic pathway and signal transduction pathways for differentially expressed genes (DEGs).

$$P=1-\sum_{i=0}^{m-1}\frac{\left({}_{i}^{M}\right)\left({}_{n-1}^{N-M}\right)}{\left({}_{n}^{N}\right)}$$

DEGs were also subjected to enrichment analysis of GO functions. The calculation formula is as above (*N* represents the number of all genes with GO annotation, n represents the number of DEGs in *N*, *M* is the number of all genes that are annotated to the specific GO terms; m represents the number of DEGs in *M*). This analysis could recognize the main biological functions of DEGs.

### 4.6. Target Gene Prediction

Based on the sequence results of all miRNAs, including the existing miRNAs, known miRNAs and novel miRNAs, we used the software patmatch (v1.2, https://www.arabidopsis.org/cgi-bin/patmatch/nph-patmatch.pl) to predict the target genes. The candidate target mRNAs were predicted as follows: (1) No more than 4 mismatches between sRNA and target (G-U bases count as 0\.5 mismatches) and no more than two adjacent mismatches in the miRNA/target duplex; (2) no adjacent mismatches in positions 2\−12 of the miRNA/target duplex (5\′ of miRNA) and no mismatches in positions 10–11 of miRNA/target duplex; (3) No more than 2\.5 mismatches in positions 1\−12 of the of the miRNA/target duplex (5\′ of miRNA);. (4) Minimum free energy (MFE) of the miRNA/target duplex should be \>\= 74% of the MFE of the miRNA bound to its perfect complement.

### 4.7. Quantitative PCR (QPCR) Analysis and Statistical Analysis

In order to verify the miRNA, total RNA was separately isolated from leaves as mentioned above, then All-in-One™ miRNA First-Strand cDNA Synthesis Kit (GeneCopoeia QP013, Rockville, MD, USA) was used for reverse transcription, according to the manufacturer’s instructions. The reaction mixture was gently mixed, after brief centrifugation and incubation for 60 min at 37 °C. Then at 85 °C incubated for 5 min (termination of the reverse transcription reaction). Expression levels of miRNA were investigated by Quantitative PCR (QPCR) (ABI Stepone Plus, Boston, MA, USA). The QPCR analysis was performed in a 20 μL reaction system using GoTaq qPCR Master Mix (A6002, Promega, Fitchburg, WI, USA), which contained 10 μL 2 ×qPCR Master Mix, 0.5 μL Forward primer (10 μM), 0.5 μL Reverse primer (10 μM), 1.0 μL cDNA template (the reverse transcription product is diluted 5 times) and 8.0 μL ddH_2_O. The small RNA-specific primers for QPCR are listed in Table 2. The QPCR conditions consisted of an initial denaturation at 95 °C for 10 min, followed by 40 cycles of denaturation at 95 °C for 15 s, annealing at 60 °C for 20 s, and extension at 72 °C for 20 s, then dissociation at 95 °C for 15 s, 60 °C for 1 min and 95 °C for 15 s. Experiments were performed in triplicate. The comparative *C*_t_ method was used to analyze the data, firstly the 2^−ΔΔ*C*t^ was calculated and then the standard (STD) was calculated for analysis.

## 5. Conclusions

The results of methyl jasmonate responsive microRNA transcriptome on *Euphorbia kansui* showed that miRNA may be involved in the response of *E. kansui* plant to exogenous MeJA. KEGG pathway analysis, GO enrichment on RNA-Seq data and QPCR verification showed that miRNAs might play roles on terpenoid biosynthesis through their target RNAs in *E. kansui* response to MeJA. In addition, based on the results of KEGG enrichment analysis on miRNA targets, miRNA might also be involved in glutathione metabolism and ether lipid metabolism. However, further studies would be needed in the future.

## Figures and Tables

**Table 1:** Methyl jasmonate (MeJA)-responsive differentially expressed miRNAs (DEGs) in terpenoid biosynthesis of Kyoto encyclopedia of genes and genomes (KEGG) analysis.

Pathway	DEGs Genes with Pathway Annotation					
	CK-VS-T1 (318)	CK-VS-T2 (174)	CK-VS-T3 (156)	T1-VS-T2 (218)	T1-VS-T3 (262)	T2-VS-T3 (125)
Terpenoid backbone biosynthesis	2 (0.63%)	1 (0.57%)	1 (0.64%)	4 (1.83%)	4 (1.53%)	1 (0.8%)
Ubiquinone and other terpenoid-quinone biosynthesis	3 (0.94%)	2 (1.15%)	1 (0.64%)	1 (0.46%)	2 (0.76%)	2 (1.6%)
Steroid biosynthesis	6 (1.89%)	2 (1.15%)	5 (3.21%)	1 (0.46%)	3 (1.15%)	3 (2.4%)
Sesquiterpenoid and triterpenoid biosynthesis	6 (1.89%)	1 (0.57%)	4(2.56%)	2 (0.92%)	3 (1.15%)	2 (1.6%)
Diterpenoid biosynthesis	0	0	0	1 (0.46%)	2 (0.76%)	0

CK, T1, T2 and T3 represent 0 h, 24 h, 36 h and 48 h MeJA treatment respectively.

**Table 2:** The miRNA specific primers for expression verification of selected MeJA responsive DEMs in terpenoid biosynthesis by real-time Quantitative PCR Detecting System (QPCR).

RNA Name	Small RNA Primer
Kansui U6-11537-F	CATACAAATACCACGAAACCTC
Kansui U6-11537-R	TGATCTGCTTCACGATGCTAC
MIR1446-x	TTGAACTCTCTCCCTCAT
MIR535-y	TTGACAAAGAGAGAGAGCACA
MIR-845-y	TGCTCTGATACCACTTGA
MIR-394-y	CTCTGTTGGTCTCTCTTTG
MIR-5563-x	TATCAGGCAACTCTTTCC
novel-m0022-5p	TTGGAATACTGTTGAGAAGCAC
novel-m0364-3p	TTTGGGCATGTTGGATAAAA

## Supplementary Materials

Supplementary Materials can be found at https://www.mdpi.com/1422-0067/20/6/1267/s1.
