# Supplementary material for: MicroRNAome Profile of Euphorbia kansui in Response to Methyl Jasmonate
Source: Int J Mol Sci. 2019 Mar 13;20(6):1267. doi: 10.3390/ijms20061267 (PMC6471261; doi:10.3390/ijms20061267)
Supplement: Supplementary file 1 [file ijms-20-01267-s001.zip › ijms-432617-SI-final/Table S1.doc]

**Additional file(1) Table S1:** Statistics of miRNA sequences in response to MeJA treatment of *Euphorbia kansui* from the individual libraries.

|  | CK | | T1 | | T2 | | T3 | |
| --- | --- | --- | --- | --- | --- | --- | --- | --- |
|  | CK1 | CK2 | T1-1 | T1-2 | T2-1 | T2-2 | T3-1 | T3-2 |
| total reads | 14388846 | 12351316 | 12031311 | 14334780 | 14756498 | 14955951 | 15196075 | 12307706 |
| high quality reads | 14118394 | 11742673 | 11793923 | 14082004 | 14458531 | 14633343 | 14832007 | 12079466 |
| clean_reads | 10293875 | 7715305 | 9866402 | 11727057 | 6583270 | 10268043 | 10094894 | 8794507 |
| Total unique Tags | 644550 | 555514 | 310505 | 450388 | 412077 | 608423 | 5962801 | 526411 |
| Unique Mapped Tags | 186344 | 147068 | 89160 | 128308 | 130317 | 166944 | 156566 | 143462 |
| Kn-miRs | 91 | 91 | 67 | 82 | 97 | 93 | 83 | 86 |
| Kn-miRs Unique Tags | 3634 | 5591 | 1968 | 3107 | 3822 | 3731 | 3736 | 3167 |
| Nov-miRs | 505 | 536 | 302 | 433 | 482 | 536 | 555 | 553 |
| Nov-miRs Unique Tags | 832 | 862 | 451 | 639 | 776 | 871 | 875 | 868 |
| snRNA | 14480 | 8531 | 9062 | 11253 | 11839 | 10064 | 8915 | 7948 |
| unann | 625604 | 540530 | 299024 | 435389 | 395640 | 593757 | 582755 | 514257 |

Notes: CK, T1, T2 and T3 represent 0 h, 24 h, 36 h and 48 h MeJA treatment respectively (two biological replicates for each treatment, named CK-1, CK-2, T1-1, T1-2, T2-1, T2-2, T3-1 and T3-2).
