# Supplementary material for: MicroRNAome Profile of Euphorbia kansui in Response to Methyl Jasmonate
Source: Int J Mol Sci. 2019 Mar 13;20(6):1267. doi: 10.3390/ijms20061267 (PMC6471261; doi:10.3390/ijms20061267)
Supplement: Supplementary file 1 [file ijms-20-01267-s001.zip › ijms-432617-SI-final/Table S10.doc]

**Additional File10 Table S10: The DEMs and their targets in [terpenoid biosynthesis](../../../../H:%5C%E6%9D%8E%E9%B9%8F%5CSGR1026_Euphorbia_kansu_12_RNAdenovo%5CSGR1026_Euphorbia_kansu_12_RNAdenovo%5C4.Basic_Annotation%5CKEGG%5CEuphorbia_kansu.htm#gene50%23gene50) and other pathway associated with MeJA treatment**

| **Pathway** | **CK_vs_T1** GeneID | Description | miRNA |
| --- | --- | --- | --- |
| Sesquiterpenoid and  triterpenoid biosynthesis | Unigene003865 | PREDICTED: beta-amyrin synthase-like  isoform X4 [Populus euphratica] | MIR1863-y, novel-m0277-3p |
|  | Unigene006820 | beta-amyrin synthase [Euphorbia tirucalli] | MIR535-z |
|  | Unigene045324 | squalene synthetase [Euphorbia tirucalli] | MIR8565-x |
|  | Unigene069286 | multifunctional triterpene synthase  [Kandelia candel] | novel-m0330-3p |
|  | Unigene073014 | squalene monooxygenase [Jatropha curcas] | MIR8175-y;novel-m0568-3p |
|  | Unigene076528 | Squalene monooxygenase [Glycine soja] | MIR1134-z;novel-m0168-p;  novel-m0225-5p;novel-m0595-3p |
| Ether lipid metabolism | Unigene027979 | PREDICTED: choline/ethanol aminephosphotransferase  1 [Jatropha curcas] | MIR6483-y;novel-m0375-3p |
|  | Unigene031929 | triacylglycerol lipase [Galdieria sulphuraria] | novel-m0540-3p |
|  | Unigene038303 | Phospholipase D delta -like protein  [Gossypium arboreum] | novel-m0027-5p;novel-m0123-5p  novel-m0134-3p;novel-m0164-3p  novel-m0244-3p;novel-m0269-3p  novel-m0334-3p;novel-m0350-3p  novel-m0361-3p;novel-m0374-5p  novel-m0386-3p;novel-m0387-3p  novel-m0395-5p;novel-m0498-3p  novel-m0499-3p;novel-m0526-3p  novel-m0593-5p;novel-m0626-3p  novel-m0628-3p |
|  | Unigene038305 | PREDICTED: phospholipase D beta 1-like  [Jatropha curcas] | novel-m0205-5p |
|  |  |  |  |
|  | Unigene068989 | PREDICTED: lysophospholipid acyltransferase LPEAT2 isoform X1 [Jatropha curcas] | novel-m0332-5p |
|  | Unigene090552 | PREDICTED: phospholipase D p1-like  [Cicer arietinum] | MIR7726-x;MIR8177-x;  MIR8762-x |
| Steroid biosynthesis | Unigene031929 | triacylglycerol lipase [Galdieria sulphuraria] | novel-m0540-3p |
|  | Unigene045324 | squalene synthetase [Euphorbia tirucalli] | MIR8565-x |
|  | Unigene049481 | PREDICTED: triacylglycerol lipase 2-like  [Jatropha curcas] | MIR2275-x;novel-m0041-5p |
|  | Unigene057348 | PREDICTED: 3beta-hydroxysteroid-dehydrogenase/decarboxylase isoform 3 [Jatropha curcas] | MIR6034-y;novel-m0527-5p |
|  | Unigene073014 | squalene monooxygenase [Jatropha curcas] | MIR8175-y;novel-m0568-3p |
|  | Unigene076528 | Squalene monooxygenase [Glycine soja] | MIR1134-z;novel-m0168-5p;  novel-m0225-5p;  novel-m0595-3p |
| Glutathione metabolism | Unigene028077 | PREDICTED: glutathione reductase, cytosolic-like [Malus domestica] | novel-m0076-3p;novel-m0247-5p  novel-m0415-3p;novel-m0438-5p  novel-m0439-5p;novel-m0639-5p |
|  | Unigene045549 | spermine synthase [Nicotiana benthamiana] | MIR1134-z |
|  | Unigene045554 | spermidine synthase [Linum usitatissimum] | MIR1134-z |
|  | Unigene047291 | PREDICTED: glucose-6-phosphate 1-dehydrogenase, cytoplasmic isoform-like [Pyrus x bretschneideri] | novel-m0039-3p;novel-m0244-3p  novel-m0269-3p;novel-m0334-3p  novel-m0350-3p;novel-m0386-3p  novel-m0387-3p;novel-m0498-3p  novel-m0499-3p;novel-m0526-3p |
|  | Unigene049661 | PREDICTED: puromycin-sensitive aminopeptidase isoform X2 [Sesamum indicum] | novel-m0039-3p |
|  | Unigene054883 | PREDICTED: glucose-6-phosphate 1-dehydrogenase, chloroplastic-like [Eucalyptus grandis] | novel-m0071-5p;novel-m0072-5p  novel-m0073-5p |
|  | Unigene068358 | PREDICTED: probable glutathione S-transferase [Jatropha curcas] | MIR8565-x |
|  | Unigene081678 | PREDICTED: cytosolic isocitrate dehydrogenase [NADP]-like [Pyrus x bretschneideri] | novel-m0136-3p;novel-m0540-3p |
|  | Unigene081679 | Isocitrate dehydrogenase [Medicago truncatula] | novel-m0136-3p;novel-m0540-3p |
|  | Unigene081680 | NADP-dependent isocitrate dehydrogenase family protein [Populus trichocarpa] | novel-m0540-3p |
|  | Unigene085559 | PREDICTED: glutathione S-transferase F9-like [Citrus sinensis] | novel-m0015-3p;novel-m0447-3p  novel-m0448-3p;novel-m0554-5p  novel-m0556-3p |
|  | Unigene087760 | glutathione reductase [Nicotiana tabacum] | novel-m0026-5p;novel-m0294-5p  novel-m0528-3p |
| [Terpenoid backbone biosynthesis](../../../../E:%5CRNA和小RNA数据%5C有用数据库%5C9月26日9万smallRNA删除样本重做差异分析%5C9万SGR1026-Euphorbia_kansu-12-smallRNA_result%5C3.target_goko%5Cgoko%5Call_mirna%5Cgroup_diff%5CKO%5CCK_vs_T2%5CCK_vs_T2_map%5Cmap00900.html) | Unigene003418 | PREDICTED:4-hydroxy-3-methylbut-2-enyl diphosphate reductase, chloroplastic  [Jatropha curcas] | MIR7747-x;novel-m0022-5p |
|  | Unigene049665 | PREDICTED: dehydrodolichyl diphosphate synthase 6-like [Jatropha curcas] | MIR5139-x;MIR8155-y |
| [Ubiquinone and other terpenoid-quinone biosynthesis](../../../../E:%5CRNA和小RNA数据%5C有用数据库%5C9月26日9万smallRNA删除样本重做差异分析%5C9万SGR1026-Euphorbia_kansu-12-smallRNA_result%5C3.target_goko%5Cgoko%5Call_mirna%5Cgroup_diff%5CKO%5CCK_vs_T2%5CCK_vs_T2_map%5Cmap00130.html) | Unigene011218  Unigene062800  Unigene062801 | PREDICTED: 4-coumarate--CoA ligase 2  [Jatropha curcas]  PREDICTED: probable tocopherol O-methyltransferase, chloroplastic isoform  X2 [Jatropha curcas]  PREDICTED: probable tocopherol O-methyltransferase, chloroplastic isoform  X2 [Jatropha curcas] | novel-m0364-5p;novel-m0370-5p  MIR1134-z  MIR1134-z |
| **Pathway** | **CK_vs_T2 (174)** | **All (12228)** | **Pvalue** |
| Glutathione metabolism | Unigene028077 | PREDICTED: glutathione reductase, cytosolic-like [Malus domestica] | novel-m0076-3p;novel-m0247-5p  novel-m0415-3p;novel-m0438-5p  novel-m0439-5p;novel-m0639-5p |
|  | Unigene031053 | spermidine synthase [Panax ginseng] | MIR2916-x |
|  | Unigene045549 | spermine synthase [Nicotiana benthamiana] | MIR1134-z |
|  | Unigene045554 | spermidine synthase [Linum usitatissimum] | MIR1134-z |
|  | Unigene047291 | PREDICTED: glucose-6-phosphate 1-dehydrogenase, cytoplasmic isoform-like [Pyrus x bretschneideri] | novel-m0039-3p;novel-m0244-3p  novel-m0269-3p;novel-m0334-3p  novel-m0350-3p;novel-m0386-3p  novel-m0387-3p;novel-m0498-3p  novel-m0499-3p;novel-m0526-3p |
|  | Unigene048650 | PREDICTED: ribonucleoside-diphosphate reductase large subunit-like [Populus euphratica] | MIR7783-y |
|  | Unigene054883 | PREDICTED: glucose-6-phosphate 1-dehydrogenase, chloroplastic-like [Eucalyptus grandis] | novel-m0071-5p;novel-m0072-5p  novel-m0073-5p |
|  | Unigene081678 | PREDICTED: cytosolic isocitrate dehydrogenase [NADP]-like [Pyrus x bretschneideri] | novel-m0136-3p;novel-m0540-3p |
|  | Unigene081679 | Isocitrate dehydrogenase [Medicago truncatula] | novel-m0136-3p;novel-m0540-3p |
|  | Unigene081680 | NADP-dependent isocitrate dehydrogenase family protein [Populus trichocarpa] | novel-m0540-3p |
|  | Unigene087760 | glutathione reductase [Nicotiana tabacum] | novel-m0026-5p;novel-m0294-5p  novel-m0528-3p |
| Ether lipid metabolism | Unigene031929 | triacylglycerol lipase [Galdieria sulphuraria] | novel-m0540-3p |
|  | Unigene038303 | Phospholipase D delta -like protein  [Gossypium arboreum] | novel-m0027-5p;novel-m0123-5p  novel-m0134-3p;novel-m0164-3p  novel-m0244-3p;novel-m0269-3p  novel-m0334-3p;novel-m0350-3p  novel-m0361-3p;novel-m0374-5p  novel-m0386-3p;novel-m0387-3p  novel-m0395-5p;novel-m0498-3p  novel-m0499-3p;novel-m0526-3p  novel-m0593-5p;novel-m0626-3p  novel-m0628-3p |
|  | Unigene068989 | PREDICTED: lysophospholipid acyltransferase LPEAT2 isoform X1 [Jatropha curcas] | novel-m0332-5p |
| Sesquiterpenoid and triterpenoid biosynthesis | Unigene076528 | Squalene monooxygenase [Glycine soja] | MIR1134-z;novel-m0168-p;  novel-m0225-5p;novel-m0595-3p |
| Steroid biosynthesis | Unigene031929 | triacylglycerol lipase [Galdieria sulphuraria] | novel-m0540-3p |
|  | Unigene076528 | Squalene monooxygenase [Glycine soja] | MIR1134-z;novel-m0168-p;  novel-m0225-5p;novel-m0595-3p |
| [Terpenoid backbone biosynthesis](../../../../E:%5CRNA和小RNA数据%5C有用数据库%5C9月26日9万smallRNA删除样本重做差异分析%5C9万SGR1026-Euphorbia_kansu-12-smallRNA_result%5C3.target_goko%5Cgoko%5Call_mirna%5Cgroup_diff%5CKO%5CCK_vs_T2%5CCK_vs_T2_map%5Cmap00900.html) | Unigene023256 | PREDICTED: probable phytol kinase 3, chloroplastic [Jatropha curcas] | novel-m0252-5p;novel-m0323-3p  novel-m0432-3p;novel-m0491-5p  novel-m0492-5p;novel-m0623-3p |
| [Ubiquinone and other terpenoid-quinone biosynthesis](../../../../E:%5CRNA和小RNA数据%5C有用数据库%5C9月26日9万smallRNA删除样本重做差异分析%5C9万SGR1026-Euphorbia_kansu-12-smallRNA_result%5C3.target_goko%5Cgoko%5Call_mirna%5Cgroup_diff%5CKO%5CCK_vs_T2%5CCK_vs_T2_map%5Cmap00130.html) | Unigene062800  Unigene062801 | PREDICTED: probable tocopherol O-methyltransferase, chloroplastic isoform  X2 [Jatropha curcas]  PREDICTED: probable tocopherol O-methyltransferase, chloroplastic isoform  X2 [Jatropha curcas] | MIR1134-z  MIR1134-z |
| **Pathway** | **CK_vs_T3 (156)** | **All (12228)** | **Pvalue** |
| Sesquiterpenoid and triterpenoid biosynthesis | Unigene003865 | PREDICTED: beta-amyrin synthase-like  isoform X4 [Populus euphratica] | MIR1863-y, novel-m0277-3p |
|  | Unigene045324 | squalene synthetase [Euphorbia tirucalli] | MIR8565-x |
|  | Unigene073014 | squalene monooxygenase [Jatropha curcas] | MIR8175-y;novel-m0568-3p |
|  | Unigene076528 | Squalene monooxygenase [Glycine soja] | MIR1134-z;novel-m0168-p;  novel-m0225-5p;novel-m0595-3p |
| Steroid biosynthesis | Unigene020790 | Cell elongation protein / DWARF1 / DIMINUTO isoform 2 [Theobroma cacao] | MIR159-y;MIR319-y |
|  | Unigene045324 | squalene synthetase [Euphorbia tirucalli] | MIR8565-x |
|  | Unigene049481 | PREDICTED: triacylglycerol lipase 2-like  [Jatropha curcas] | MIR2275-x;novel-m0041-5p |
|  | Unigene073014 | squalene monooxygenase [Jatropha curcas] | MIR8175-y;novel-m0568-3p |
|  | Unigene076528 | Squalene monooxygenase [Glycine soja] | MIR1134-z;novel-m0168-5p;  novel-m0225-5p;  novel-m0595-3p |
| Glutathione metabolism | Unigene028077 | PREDICTED: glutathione reductase, cytosolic-like [Malus domestica] | novel-m0076-3p;novel-m0247-5p  novel-m0415-3p;novel-m0438-5p  novel-m0439-5p;novel-m0639-5p |
|  | Unigene036111 | PREDICTED: L-ascorbate peroxidase 3, peroxisomal [Sesamum indicum] | novel-m0360-5p |
|  | Unigene047291 | PREDICTED: glucose-6-phosphate 1-dehydrogenase, cytoplasmic isoform-like [Pyrus x bretschneideri] | novel-m0039-3p;novel-m0244-3p  novel-m0269-3p;novel-m0334-3p  novel-m0350-3p;novel-m0386-3p  novel-m0387-3p;novel-m0498-3p  novel-m0499-3p;novel-m0526-3p |
|  | Unigene049661 | PREDICTED: puromycin-sensitive aminopeptidase isoform X2 [Sesamum indicum] | novel-m0039-3p |
|  | Unigene054883 | PREDICTED: glucose-6-phosphate 1-dehydrogenase, chloroplastic-like [Eucalyptus grandis] | novel-m0071-5p;novel-m0072-5p  novel-m0073-5p |
|  | Unigene068358 | PREDICTED: probable glutathione S-transferase [Jatropha curcas] | MIR8565-x |
| Ether lipid metabolism | Unigene038303 | Phospholipase D delta -like protein  [Gossypium arboreum] | novel-m0027-5p;novel-m0123-5p  novel-m0134-3p;novel-m0164-3p  novel-m0244-3p;novel-m0269-3p  novel-m0334-3p;novel-m0350-3p  novel-m0361-3p;novel-m0374-5p  novel-m0386-3p;novel-m0387-3p  novel-m0395-5p;novel-m0498-3p  novel-m0499-3p;novel-m0526-3p  novel-m0593-5p;novel-m0626-3p  novel-m0628-3p |
|  | Unigene068989 | PREDICTED: lysophospholipid acyltransferase LPEAT2 isoform X1 [Jatropha curcas] | novel-m0332-5p |
| [Ubiquinone and other terpenoid-quinone biosynthesis](../../../../E:%5CRNA%E5%92%8C%E5%B0%8FRNA%E6%95%B0%E6%8D%AE%5C%E6%9C%89%E7%94%A8%E6%95%B0%E6%8D%AE%E5%BA%93%5C9%E6%9C%8826%E6%97%A59%E4%B8%87smallRNA%E5%88%A0%E9%99%A4%E6%A0%B7%E6%9C%AC%E9%87%8D%E5%81%9A%E5%B7%AE%E5%BC%82%E5%88%86%E6%9E%90%5C9%E4%B8%87SGR1026-Euphorbia_kansu-12-smallRNA_result%5C3.target_goko%5Cgoko%5Call_mirna%5Cgroup_diff%5CKO%5CCK_vs_T3%5CCK_vs_T3.htm#gene77)  [Terpenoid backbone biosynthesis](../../../../E:%5CRNA%E5%92%8C%E5%B0%8FRNA%E6%95%B0%E6%8D%AE%5C%E6%9C%89%E7%94%A8%E6%95%B0%E6%8D%AE%E5%BA%93%5C9%E6%9C%8826%E6%97%A59%E4%B8%87smallRNA%E5%88%A0%E9%99%A4%E6%A0%B7%E6%9C%AC%E9%87%8D%E5%81%9A%E5%B7%AE%E5%BC%82%E5%88%86%E6%9E%90%5C9%E4%B8%87SGR1026-Euphorbia_kansu-12-smallRNA_result%5C3.target_goko%5Cgoko%5Call_mirna%5Cgroup_diff%5CKO%5CCK_vs_T3%5CCK_vs_T3.htm#gene85) | Unigene011218  Unigene023256 | PREDICTED: 4-coumarate--CoA ligase 2  [Jatropha curcas]  PREDICTED: probable phytol kinase 3, chloroplastic [Jatropha curcas] | novel-m0364-5p;novel-m0370-5p  novel-m0252-5p;novel-m0323-3p  novel-m0432-3p;novel-m0491-5p  novel-m0492-5p;novel-m0623-3p |
| **Pathway** | **T1_vs_T2 (218)** | **All (12228)** | **Pvalue** |
| Ether lipid metabolism | Unigene038303 | Phospholipase D delta -like protein  [Gossypium arboreum] | novel-m0027-5p;novel-m0123-5p  novel-m0134-3p;novel-m0164-3p  novel-m0244-3p;novel-m0269-3p  novel-m0334-3p;novel-m0350-3p  novel-m0361-3p;novel-m0374-5p  novel-m0386-3p;novel-m0387-3p  novel-m0395-5p;novel-m0498-3p  novel-m0499-3p;novel-m0526-3p  novel-m0593-5p;novel-m0626-3p  novel-m0628-3p |
|  | Unigene027747 | PREDICTED: non-specific phospholipase C2 [Jatropha curcas] | MIR172-y |
|  | Unigene027979 | PREDICTED: choline/ethanolaminephosphotransferase  1 [Jatropha curcas] | MIR6483-y;novel-m0375-3p |
|  | Unigene090552 | PREDICTED: phospholipase D p1-like  [Cicer arietinum] | MIR7726-x;MIR8177-x;  MIR8762-x |
|  | Unigene095384 | PREDICTED: phospholipase D epsilon [Jatropha curcas] | novel-m0203-5p |
| Sesquiterpenoid and triterpenoid biosynthesis | Unigene006820 | beta-amyrin synthase [Euphorbia tirucalli] | MIR535-z |
|  | Unigene045324 | squalene synthetase [Euphorbia tirucalli] | MIR8565-x |
| Glutathione metabolism | Unigene028077 | PREDICTED: glutathione reductase, cytosolic-like [Malus domestica] | novel-m0076-3p;novel-m0247-5p  novel-m0415-3p;novel-m0438-5p  novel-m0439-5p;novel-m0639-5p |
|  | Unigene047291 | PREDICTED: glucose-6-phosphate 1-dehydrogenase, cytoplasmic isoform-like [Pyrus x bretschneideri] | novel-m0039-3p;novel-m0244-3p  novel-m0269-3p;novel-m0334-3p  novel-m0350-3p;novel-m0386-3p  novel-m0387-3p;novel-m0498-3p  novel-m0499-3p;novel-m0526-3p |
|  | Unigene049661 | PREDICTED: puromycin-sensitive aminopeptidase isoform X2 [Sesamum indicum] | novel-m0039-3p |
|  | Unigene085559 | PREDICTED: glutathione S-transferase F9-like [Citrus sinensis] | novel-m0015-3p;novel-m0447-3p  novel-m0448-3p;novel-m0554-5p  novel-m0556-3p |
|  | Unigene087760 | glutathione reductase [Nicotiana tabacum] | novel-m0026-5p;novel-m0294-5p  novel-m0528-3p |
|  | Unigene068358 | PREDICTED: probable glutathione  S-transferase [Jatropha curcas] | MIR8565-x |
| Steroid biosynthesis | Unigene045324 | squalene synthetase [Euphorbia tirucalli] | MIR8565-x |
| [Ubiquinone and other terpenoid-quinone biosynthesis](../../../../E:%5CRNA%E5%92%8C%E5%B0%8FRNA%E6%95%B0%E6%8D%AE%5C%E6%9C%89%E7%94%A8%E6%95%B0%E6%8D%AE%E5%BA%93%5C9%E6%9C%8826%E6%97%A59%E4%B8%87smallRNA%E5%88%A0%E9%99%A4%E6%A0%B7%E6%9C%AC%E9%87%8D%E5%81%9A%E5%B7%AE%E5%BC%82%E5%88%86%E6%9E%90%5C9%E4%B8%87SGR1026-Euphorbia_kansu-12-smallRNA_result%5C3.target_goko%5Cgoko%5Call_mirna%5Cgroup_diff%5CKO%5CCK_vs_T3%5CCK_vs_T3.htm#gene77)  [Terpenoid backbone biosynthesis](../../../../E:%5CRNA%E5%92%8C%E5%B0%8FRNA%E6%95%B0%E6%8D%AE%5C%E6%9C%89%E7%94%A8%E6%95%B0%E6%8D%AE%E5%BA%93%5C9%E6%9C%8826%E6%97%A59%E4%B8%87smallRNA%E5%88%A0%E9%99%A4%E6%A0%B7%E6%9C%AC%E9%87%8D%E5%81%9A%E5%B7%AE%E5%BC%82%E5%88%86%E6%9E%90%5C9%E4%B8%87SGR1026-Euphorbia_kansu-12-smallRNA_result%5C3.target_goko%5Cgoko%5Call_mirna%5Cgroup_diff%5CKO%5CT1_vs_T2%5CT1_vs_T2.htm#gene24) | Unigene011218  Unigene003418  Unigene023256 | PREDICTED: 4-coumarate--CoA ligase 2  [Jatropha curcas]  PREDICTED: 4-hydroxy-3-methylbut-2-enyl diphosphate reductase, chloroplastic [Jatropha curcas] | novel-m0364-5p;novel-m0370-5p  MIR7747-x;novel-m0022-5p |
|  | Unigene049665  Unigene055774 | PREDICTED: dehydrodolichyl diphosphate synthase 6-like [Jatropha curcas]  1-deoxy-D-xylulose 5-phosphate reductoisomerase [Euphorbia lathyris] | MIR5139-x;MIR8155-y  novel-m0389-5p |
| **Pathway** | **T1_vs_T3 (262)** | **All (12228)** | **Pvalue** |
| Ether lipid metabolism | Unigene027979 | PREDICTED: choline/ethanolaminephosphotransferase  1 [Jatropha curcas] | MIR6483-y;novel-m0375-3p |
|  | Unigene031929 | triacylglycerol lipase [Galdieria sulphuraria] | novel-m0540-3p |
|  | Unigene038303 | Phospholipase D delta -like protein  [Gossypium arboreum] | novel-m0027-5p;novel-m0123-5p  novel-m0134-3p;novel-m0164-3p  novel-m0244-3p;novel-m0269-3p  novel-m0334-3p;novel-m0350-3p  novel-m0361-3p;novel-m0374-5p  novel-m0386-3p;novel-m0387-3p  novel-m0395-5p;novel-m0498-3p  novel-m0499-3p;novel-m0526-3p  novel-m0593-5p;novel-m0626-3p  novel-m0628-3p |
|  | Unigene038305 | PREDICTED: phospholipase D beta 1-like  [Jatropha curcas] | novel-m0205-5p |
|  | Unigene090552 | PREDICTED: phospholipase D p1-like  [Cicer arietinum] | MIR7726-x;MIR8177-x;  MIR8762-x |
|  | Unigene094438 | PREDICTED: non-specific phospholipase C1 [Cucumis melo] | novel-m0316-5p;novel-m0644-5p |
| Glutathione metabolism | Unigene006447 | membrane alanylamino-peptidase [Medicago truncatula var. truncatula] | novel-m0411-5p |
|  | Unigene028077 | PREDICTED: glutathione reductase, cytosolic-like [Malus domestica] | novel-m0076-3p;novel-m0247-5p  novel-m0415-3p;novel-m0438-5p  novel-m0439-5p;novel-m0639-5p |
|  | Unigene047291 | PREDICTED: glucose-6-phosphate 1-dehydrogenase, cytoplasmic isoform-like [Pyrus x bretschneideri] | novel-m0039-3p;novel-m0244-3p  novel-m0269-3p;novel-m0334-3p  novel-m0350-3p;novel-m0386-3p  novel-m0387-3p;novel-m0498-3p  novel-m0499-3p;novel-m0526-3p |
|  | Unigene049661 | PREDICTED: puromycin-sensitive aminopeptidase isoform X2 [Sesamum indicum] | novel-m0039-3p |
|  | Unigene054883 | PREDICTED: glucose-6-phosphate 1-dehydrogenase, chloroplastic-like [Eucalyptus grandis] | novel-m0071-5p;novel-m0072-5p  novel-m0073-5p |
|  | Unigene068358 | PREDICTED: probable glutathione  S-transferase [Jatropha curcas] | MIR8565-x |
|  | Unigene081678 | PREDICTED: cytosolic isocitrate dehydrogenase [NADP]-like [Pyrus x bretschneideri] | novel-m0136-3p;novel-m0540-3p |
|  | Unigene081679 | Isocitrate dehydrogenase [Medicago truncatula] | novel-m0136-3p;novel-m0540-3p |
|  | Unigene081680 | NADP-dependent isocitrate dehydrogenase family protein [Populus trichocarpa] | novel-m0540-3p |
|  | Unigene087760 | glutathione reductase [Nicotiana tabacum] | novel-m0026-5p;novel-m0294-5p  novel-m0528-3p |
|  | Unigene089725 | PREDICTED: puromycin-sensitive aminopeptidase-like isoform X2 [Citrus sinensis] | novel-m0411-5p |
|  | Unigene089726 | PREDICTED: LOW QUALITY PROTEIN: puromycin-sensitive aminopeptidase [Malus domestica] | novel-m0411-5p |
| Sesquiterpenoid and triterpenoid biosynthesis | Unigene006820 | beta-amyrin synthase [Euphorbia tirucalli] | MIR535-z |
|  | Unigene045324 | squalene synthetase [Euphorbia tirucalli] | MIR8565-x |
|  | Unigene073014 | squalene monooxygenase [Jatropha curcas] | MIR8175-y;novel-m0568-3p |
| Steroid biosynthesis | Unigene031929 | triacylglycerol lipase [Galdieria sulphuraria] | novel-m0540-3p |
|  | Unigene045324 | squalene synthetase [Euphorbia tirucalli] | MIR8565-x |
|  | Unigene073014 | squalene monooxygenase [Jatropha curcas] | MIR8175-y;novel-m0568-3p |
| [Ubiquinone and other terpenoid-quinone biosynthesis](../../../../E:%5CRNA%E5%92%8C%E5%B0%8FRNA%E6%95%B0%E6%8D%AE%5C%E6%9C%89%E7%94%A8%E6%95%B0%E6%8D%AE%E5%BA%93%5C9%E6%9C%8826%E6%97%A59%E4%B8%87smallRNA%E5%88%A0%E9%99%A4%E6%A0%B7%E6%9C%AC%E9%87%8D%E5%81%9A%E5%B7%AE%E5%BC%82%E5%88%86%E6%9E%90%5C9%E4%B8%87SGR1026-Euphorbia_kansu-12-smallRNA_result%5C3.target_goko%5Cgoko%5Call_mirna%5Cgroup_diff%5CKO%5CCK_vs_T3%5CCK_vs_T3.htm#gene77) | Unigene011218  Unigene045546 | PREDICTED: 4-coumarate--CoA ligase 2 [Jatropha curcas]  RecName: Full=Trans-cinnamate 4-monooxygenase; AltName: Full=Cinnamic acid 4-hydroxylase; Short=C4H; Short=CA4H; AltName: Full=Cytochrome P450 73; AltName: Full=Cytochrome P450C4H [Catharanthus roseus] | novel-m0364-5p;novel-m0370-5p  novel-m0324-3p |
| [Terpenoid backbone biosynthesis](../../../../E:%5CRNA%E5%92%8C%E5%B0%8FRNA%E6%95%B0%E6%8D%AE%5C%E6%9C%89%E7%94%A8%E6%95%B0%E6%8D%AE%E5%BA%93%5C9%E6%9C%8826%E6%97%A59%E4%B8%87smallRNA%E5%88%A0%E9%99%A4%E6%A0%B7%E6%9C%AC%E9%87%8D%E5%81%9A%E5%B7%AE%E5%BC%82%E5%88%86%E6%9E%90%5C9%E4%B8%87SGR1026-Euphorbia_kansu-12-smallRNA_result%5C3.target_goko%5Cgoko%5Call_mirna%5Cgroup_diff%5CKO%5CT1_vs_T2%5CT1_vs_T2.htm#gene24) | Unigene003418  Unigene023256  Unigene049665  Unigene055774 | PREDICTED: 4-hydroxy-3-methylbut-2-enyl diphosphate reductase, chloroplastic [Jatropha curcas]  PREDICTED: probable phytol kinase 3, chloroplastic [Jatropha curcas]  PREDICTED: dehydrodolichyl diphosphate synthase 6-like [Jatropha curcas]  1-deoxy-D-xylulose 5-phosphate reductoisomerase [Euphorbia lathyris] | MIR7747-x;novel-m0022-5p  novel-m0252-5p;novel-m0323-3p  novel-m0432-3p;novel-m0491-5p  novel-m0492-5p;novel-m0623-3p  MIR5139-x;MIR8155-y  novel-m0389-5p |
| **Pathway** | **T2_vs_T3 (125)** | **All (12228)** | **Pvalue** |
| Glutathione metabolism | Unigene031053 | spermidine synthase [Panax ginseng] | MIR2916-x |
|  | Unigene047291 | PREDICTED: glucose-6-phosphate 1-dehydrogenase, cytoplasmic isoform-like [Pyrus x bretschneideri] | novel-m0039-3p;novel-m0244-3p  novel-m0269-3p;novel-m0334-3p  novel-m0350-3p;novel-m0386-3p  novel-m0387-3p;novel-m0498-3p  novel-m0499-3p;novel-m0526-3p |
|  | Unigene048650 | PREDICTED: ribonucleoside-diphosphate reductase large subunit-like [Populus euphratica] | MIR7783-y |
|  | Unigene049661 | PREDICTED: puromycin-sensitive aminopeptidase isoform X2 [Sesamum indicum] | novel-m0039-3p |
|  | Unigene081678 | PREDICTED: cytosolic isocitrate dehydrogenase [NADP]-like [Pyrus x bretschneideri] | novel-m0136-3p;novel-m0540-3p |
|  | Unigene081679 | Isocitrate dehydrogenase [Medicago truncatula] | novel-m0136-3p;novel-m0540-3p |
|  | Unigene081680 | NADP-dependent isocitrate dehydrogenase family protein [Populus trichocarpa] | novel-m0540-3p |
| Ether lipid metabolism | Unigene027747 | PREDICTED: non-specific phospholipase C2 [Jatropha curcas] | MIR172-y |
|  | Unigene031929 | triacylglycerol lipase [Galdieria sulphuraria] | novel-m0540-3p |
|  | Unigene038303 | Phospholipase D delta -like protein  [Gossypium arboreum] | novel-m0027-5p;novel-m0123-5p  novel-m0134-3p;novel-m0164-3p  novel-m0244-3p;novel-m0269-3p  novel-m0334-3p;novel-m0350-3p  novel-m0361-3p;novel-m0374-5p  novel-m0386-3p;novel-m0387-3p  novel-m0395-5p;novel-m0498-3p  novel-m0499-3p;novel-m0526-3p  novel-m0593-5p;novel-m0626-3p  novel-m0628-3p |
| Sesquiterpenoid and triterpenoid biosynthesis | Unigene073014 | squalene monooxygenase [Jatropha curcas] | MIR8175-y;novel-m0568-3p |
|  | Unigene076528 | Squalene monooxygenase [Glycine soja] | MIR1134-z;novel-m0168-5p  novel-m0225-5p;novel-m0595-3p |
| Steroid biosynthesis | Unigene031929  Unigene073014  Unigene076528 | triacylglycerol lipase [Galdieria sulphuraria]  squalene monooxygenase [Jatropha curcas]  Squalene monooxygenase [Glycine soja] | novel-m0540-3p  MIR8175-y;novel-m0568-3p  MIR1134-z;novel-m0168-5p  novel-m0225-5p;novel-m0595-3p |
| [Ubiquinone and other terpenoid-quinone biosynthesis](../../../../E:%5CRNA%E5%92%8C%E5%B0%8FRNA%E6%95%B0%E6%8D%AE%5C%E6%9C%89%E7%94%A8%E6%95%B0%E6%8D%AE%E5%BA%93%5C9%E6%9C%8826%E6%97%A59%E4%B8%87smallRNA%E5%88%A0%E9%99%A4%E6%A0%B7%E6%9C%AC%E9%87%8D%E5%81%9A%E5%B7%AE%E5%BC%82%E5%88%86%E6%9E%90%5C9%E4%B8%87SGR1026-Euphorbia_kansu-12-smallRNA_result%5C3.target_goko%5Cgoko%5Call_mirna%5Cgroup_diff%5CKO%5CCK_vs_T3%5CCK_vs_T3.htm#gene77) | Unigene028155  Unigene045546 | 4-hydroxyphenylpyruvate dioxygenase  RecName: Full=Trans-cinnamate 4-monooxygenase; AltName: Full=Cinnamic acid 4-hydroxylase; Short=C4H; Short=CA4H; AltName: Full=Cytochrome P450 73; AltName: Full=Cytochrome P450C4H [Catharanthus roseus] | novel-m0580-5p;novel-m0581-5p  novel-m0324-3p |
| [Terpenoid backbone biosynthesis](../../../../E:%5CRNA%E5%92%8C%E5%B0%8FRNA%E6%95%B0%E6%8D%AE%5C%E6%9C%89%E7%94%A8%E6%95%B0%E6%8D%AE%E5%BA%93%5C9%E6%9C%8826%E6%97%A59%E4%B8%87smallRNA%E5%88%A0%E9%99%A4%E6%A0%B7%E6%9C%AC%E9%87%8D%E5%81%9A%E5%B7%AE%E5%BC%82%E5%88%86%E6%9E%90%5C9%E4%B8%87SGR1026-Euphorbia_kansu-12-smallRNA_result%5C3.target_goko%5Cgoko%5Call_mirna%5Cgroup_diff%5CKO%5CT1_vs_T2%5CT1_vs_T2.htm#gene24) | Unigene049665 | PREDICTED: dehydrodolichyl diphosphate synthase 6-like [Jatropha curcas] | MIR5139-x;MIR8155-y |
