# Supplementary material for: MicroRNAome Profile of Euphorbia kansui in Response to Methyl Jasmonate
Source: Int J Mol Sci. 2019 Mar 13;20(6):1267. doi: 10.3390/ijms20061267 (PMC6471261; doi:10.3390/ijms20061267)
Supplement: Supplementary file 1 [file ijms-20-01267-s001.zip › ijms-432617-SI-final/Table S4.doc]

Additional File4 TableS4: The details of known miRNA family

| **ID** | **mir_count** |  |
| --- | --- | --- |
| **MIR1024-y** | 2 | ppt-MIR1024a,ppt-MIR1024b |
| **MIR1057-y** | 1 | ppt-MIR1057 |
| **MIR1098-x** | 1 | smo-MIR1098 |
| **MIR1134-z** | 1 | far-MIR1134,tae-MIR1134 |
| **MIR1437-x** | 1 | osa-MIR1437a,osa-MIR1437b |
| **MIR1446-x** | 1 | gra-MIR1446,mes-MIR1446,nta-MIR1446,ptc-MIR1446a,ptc-MIR1446b,ptc-MIR1446c,ptc-MIR1446d,ptc-MIR1446e |
| **MIR1448-y** | 1 | ptc-MIR1448 |
| **MIR1511-y** | 1 | gma-MIR1511,mdm-MIR1511,ppe-MIR1511 |
| **MIR1531-y** | 1 | gma-MIR1531 |
| **MIR156-x** | 1 | ahy-MIR156a,ahy-MIR156b,ahy-MIR156c,aly-MIR156a,aly-MIR156b,aly-MIR156c,aly-MIR156d,aly-MIR156e,aly-MIR156f,aly-MIR156g,aly-MIR156h,ama-MIR156,aqc-MIR156a,aqc-MIR156b,ata-MIR156a,ata-MIR156b,ata-MIR156c,ata-MIR156d,ata-MIR156e,ath-MIR156a,ath-MIR156b,ath-MIR156c,ath-MIR156d,ath-MIR156e,ath-MIR156f,ath-MIR156g,ath-MIR156h,ath-MIR156i,ath-MIR156j,atr-MIR156a,atr-MIR156b,atr-MIR156c,atr-MIR156d,bcy-MIR156,bdi-MIR156a,bdi-MIR156b,bdi-MIR156c,bdi-MIR156d,bdi-MIR156e,bdi-MIR156f,bdi-MIR156g,bdi-MIR156h,bdi-MIR156i,bdi-MIR156j,bgy-MIR156,bna-MIR156a,bna-MIR156b,bna-MIR156c,bna-MIR156d,bna-MIR156e,bna-MIR156f,bna-MIR156g,bra-MIR156a,bra-MIR156b,bra-MIR156c,bra-MIR156d,bra-MIR156e,bra-MIR156f,bra-MIR156g,cca-MIR156a,cca-MIR156b,cca-MIR156c,cme-MIR156a,cme-MIR156b,cme-MIR156c,cme-MIR156d,cme-MIR156e,cme-MIR156f,cme-MIR156g,cme-MIR156h,cme-MIR156i,cme-MIR156j,cpa-MIR156a,cpa-MIR156b,cpa-MIR156c,cpa-MIR156d,cpa-MIR156e,cpa-MIR156f,csi-MIR156,ctr-MIR156,dpr-MIR156a,dpr-MIR156b,far-MIR156a,far-MIR156b,ghr-MIR156a,ghr-MIR156b,ghr-MIR156c,ghr-MIR156d,gma-MIR156a,gma-MIR156aa,gma-MIR156ab,gma-MIR156b,gma-MIR156c,gma-MIR156d,gma-MIR156e,gma-MIR156f,gma-MIR156g,gma-MIR156h,gma-MIR156i,gma-MIR156j,gma-MIR156k,gma-MIR156l,gma-MIR156m,gma-MIR156n,gma-MIR156o,gma-MIR156p,gma-MIR156q,gma-MIR156r,gma-MIR156s,gma-MIR156t,gma-MIR156u,gma-MIR156v,gma-MIR156w,gma-MIR156x,gma-MIR156y,gma-MIR156z,han-MIR156a,han-MIR156b,han-MIR156c,har-MIR156a,har-MIR156c,hbr-MIR156,hci-MIR156a,hci-MIR156b,hpa-MIR156a,htu-MIR156a,hvu-MIR156a,hvu-MIR156b,lus-MIR156a,lus-MIR156b,lus-MIR156c,lus-MIR156d,lus-MIR156e,lus-MIR156f,lus-MIR156g,lus-MIR156h,lus-MIR156i,mdm-MIR156a,mdm-MIR156aa,mdm-MIR156ab,mdm-MIR156ac,mdm-MIR156ad,mdm-MIR156ae,mdm-MIR156b,mdm-MIR156c,mdm-MIR156d,mdm-MIR156e,mdm-MIR156f,mdm-MIR156g,mdm-MIR156h,mdm-MIR156i,mdm-MIR156j,mdm-MIR156k,mdm-MIR156l,mdm-MIR156m,mdm-MIR156n,mdm-MIR156o,mdm-MIR156p,mdm-MIR156q,mdm-MIR156r,mdm-MIR156s,mdm-MIR156t,mdm-MIR156u,mdm-MIR156v,mdm-MIR156w,mdm-MIR156x,mdm-MIR156y,mdm-MIR156z,mes-MIR156a,mes-MIR156b,mes-MIR156c,mes-MIR156d,mes-MIR156e,mes-MIR156f,mes-MIR156g,mes-MIR156h,mes-MIR156i,mes-MIR156j,mes-MIR156k,mtr-MIR156a,mtr-MIR156b,mtr-MIR156c,mtr-MIR156d,mtr-MIR156e,mtr-MIR156f,mtr-MIR156g,mtr-MIR156h,mtr-MIR156i,mtr-MIR156j,nta-MIR156a,nta-MIR156b,nta-MIR156c,nta-MIR156d,nta-MIR156e,nta-MIR156f,nta-MIR156g,nta-MIR156h,nta-MIR156i,nta-MIR156j,osa-MIR156a,osa-MIR156b,osa-MIR156c,osa-MIR156d,osa-MIR156e,osa-MIR156f,osa-MIR156g,osa-MIR156h,osa-MIR156i,osa-MIR156j,osa-MIR156k,osa-MIR156l,ppe-MIR156a,ppe-MIR156b,ppe-MIR156c,ppe-MIR156d,ppe-MIR156e,ppe-MIR156f,ppe-MIR156g,ppe-MIR156h,ppe-MIR156i,ppt-MIR156a,ppt-MIR156b,ppt-MIR156c,pta-MIR156a,pta-MIR156b,ptc-MIR156a,ptc-MIR156b,ptc-MIR156c,ptc-MIR156d,ptc-MIR156e,ptc-MIR156f,ptc-MIR156g,ptc-MIR156h,ptc-MIR156i,ptc-MIR156j,ptc-MIR156k,ptc-MIR156l,rco-MIR156a,rco-MIR156b,rco-MIR156c,rco-MIR156d,rco-MIR156e,rco-MIR156f,rco-MIR156g,rco-MIR156h,sbi-MIR156a,sbi-MIR156b,sbi-MIR156c,sbi-MIR156d,sbi-MIR156e,sbi-MIR156f,sbi-MIR156g,sbi-MIR156h,sbi-MIR156i,sly-MIR156a,sly-MIR156b,sly-MIR156c,sly-MIR156d,sly-MIR156e,smo-MIR156a,smo-MIR156b,smo-MIR156c,smo-MIR156d,sof-MIR156,ssl-MIR156,ssp-MIR156,stu-MIR156a,stu-MIR156b,stu-MIR156c,stu-MIR156d,stu-MIR156e,stu-MIR156f,stu-MIR156g,stu-MIR156h,stu-MIR156i,stu-MIR156j,stu-MIR156k,tae-MIR156,tcc-MIR156a,tcc-MIR156b,tcc-MIR156c,tcc-MIR156d,tcc-MIR156e,tcc-MIR156f,tcc-MIR156g,vun-MIR156a,vun-MIR156b,vvi-MIR156a,vvi-MIR156b,vvi-MIR156c,vvi-MIR156d,vvi-MIR156e,vvi-MIR156f,vvi-MIR156g,vvi-MIR156h,vvi-MIR156i,zma-MIR156a,zma-MIR156b,zma-MIR156c,zma-MIR156d,zma-MIR156e,zma-MIR156f,zma-MIR156g,zma-MIR156h,zma-MIR156i,zma-MIR156j,zma-MIR156k,zma-MIR156l |
| **MIR156-y** | 1 | ahy-MIR156a,ahy-MIR156b,ahy-MIR156c,aly-MIR156a,aly-MIR156b,aly-MIR156c,aly-MIR156d,aly-MIR156e,aly-MIR156f,aly-MIR156g,aly-MIR156h,ama-MIR156,aqc-MIR156a,aqc-MIR156b,ata-MIR156a,ata-MIR156b,ata-MIR156c,ata-MIR156d,ata-MIR156e,ath-MIR156a,ath-MIR156b,ath-MIR156c,ath-MIR156d,ath-MIR156e,ath-MIR156f,ath-MIR156g,ath-MIR156h,ath-MIR156i,ath-MIR156j,atr-MIR156a,atr-MIR156b,atr-MIR156c,atr-MIR156d,bcy-MIR156,bdi-MIR156a,bdi-MIR156b,bdi-MIR156c,bdi-MIR156d,bdi-MIR156e,bdi-MIR156f,bdi-MIR156g,bdi-MIR156h,bdi-MIR156i,bdi-MIR156j,bgy-MIR156,bna-MIR156a,bna-MIR156b,bna-MIR156c,bna-MIR156d,bna-MIR156e,bna-MIR156f,bna-MIR156g,bra-MIR156a,bra-MIR156b,bra-MIR156c,bra-MIR156d,bra-MIR156e,bra-MIR156f,bra-MIR156g,cca-MIR156a,cca-MIR156b,cca-MIR156c,cme-MIR156a,cme-MIR156b,cme-MIR156c,cme-MIR156d,cme-MIR156e,cme-MIR156f,cme-MIR156g,cme-MIR156h,cme-MIR156i,cme-MIR156j,cpa-MIR156a,cpa-MIR156b,cpa-MIR156c,cpa-MIR156d,cpa-MIR156e,cpa-MIR156f,csi-MIR156,ctr-MIR156,dpr-MIR156a,dpr-MIR156b,far-MIR156a,far-MIR156b,ghr-MIR156a,ghr-MIR156b,ghr-MIR156c,ghr-MIR156d,gma-MIR156a,gma-MIR156aa,gma-MIR156ab,gma-MIR156b,gma-MIR156c,gma-MIR156d,gma-MIR156e,gma-MIR156f,gma-MIR156g,gma-MIR156h,gma-MIR156i,gma-MIR156j,gma-MIR156k,gma-MIR156l,gma-MIR156m,gma-MIR156n,gma-MIR156o,gma-MIR156p,gma-MIR156q,gma-MIR156r,gma-MIR156s,gma-MIR156t,gma-MIR156u,gma-MIR156v,gma-MIR156w,gma-MIR156x,gma-MIR156y,gma-MIR156z,han-MIR156a,han-MIR156b,han-MIR156c,har-MIR156a,har-MIR156c,hbr-MIR156,hci-MIR156a,hci-MIR156b,hpa-MIR156a,htu-MIR156a,hvu-MIR156a,hvu-MIR156b,lus-MIR156a,lus-MIR156b,lus-MIR156c,lus-MIR156d,lus-MIR156e,lus-MIR156f,lus-MIR156g,lus-MIR156h,lus-MIR156i,mdm-MIR156a,mdm-MIR156aa,mdm-MIR156ab,mdm-MIR156ac,mdm-MIR156ad,mdm-MIR156ae,mdm-MIR156b,mdm-MIR156c,mdm-MIR156d,mdm-MIR156e,mdm-MIR156f,mdm-MIR156g,mdm-MIR156h,mdm-MIR156i,mdm-MIR156j,mdm-MIR156k,mdm-MIR156l,mdm-MIR156m,mdm-MIR156n,mdm-MIR156o,mdm-MIR156p,mdm-MIR156q,mdm-MIR156r,mdm-MIR156s,mdm-MIR156t,mdm-MIR156u,mdm-MIR156v,mdm-MIR156w,mdm-MIR156x,mdm-MIR156y,mdm-MIR156z,mes-MIR156a,mes-MIR156b,mes-MIR156c,mes-MIR156d,mes-MIR156e,mes-MIR156f,mes-MIR156g,mes-MIR156h,mes-MIR156i,mes-MIR156j,mes-MIR156k,mtr-MIR156a,mtr-MIR156b,mtr-MIR156c,mtr-MIR156d,mtr-MIR156e,mtr-MIR156f,mtr-MIR156g,mtr-MIR156h,mtr-MIR156i,mtr-MIR156j,nta-MIR156a,nta-MIR156b,nta-MIR156c,nta-MIR156d,nta-MIR156e,nta-MIR156f,nta-MIR156g,nta-MIR156h,nta-MIR156i,nta-MIR156j,osa-MIR156a,osa-MIR156b,osa-MIR156c,osa-MIR156d,osa-MIR156e,osa-MIR156f,osa-MIR156g,osa-MIR156h,osa-MIR156i,osa-MIR156j,osa-MIR156k,osa-MIR156l,ppe-MIR156a,ppe-MIR156b,ppe-MIR156c,ppe-MIR156d,ppe-MIR156e,ppe-MIR156f,ppe-MIR156g,ppe-MIR156h,ppe-MIR156i,ppt-MIR156a,ppt-MIR156b,ppt-MIR156c,pta-MIR156a,pta-MIR156b,ptc-MIR156a,ptc-MIR156b,ptc-MIR156c,ptc-MIR156d,ptc-MIR156e,ptc-MIR156f,ptc-MIR156g,ptc-MIR156h,ptc-MIR156i,ptc-MIR156j,ptc-MIR156k,ptc-MIR156l,rco-MIR156a,rco-MIR156b,rco-MIR156c,rco-MIR156d,rco-MIR156e,rco-MIR156f,rco-MIR156g,rco-MIR156h,sbi-MIR156a,sbi-MIR156b,sbi-MIR156c,sbi-MIR156d,sbi-MIR156e,sbi-MIR156f,sbi-MIR156g,sbi-MIR156h,sbi-MIR156i,sly-MIR156a,sly-MIR156b,sly-MIR156c,sly-MIR156d,sly-MIR156e,smo-MIR156a,smo-MIR156b,smo-MIR156c,smo-MIR156d,sof-MIR156,ssl-MIR156,ssp-MIR156,stu-MIR156a,stu-MIR156b,stu-MIR156c,stu-MIR156d,stu-MIR156e,stu-MIR156f,stu-MIR156g,stu-MIR156h,stu-MIR156i,stu-MIR156j,stu-MIR156k,tae-MIR156,tcc-MIR156a,tcc-MIR156b,tcc-MIR156c,tcc-MIR156d,tcc-MIR156e,tcc-MIR156f,tcc-MIR156g,vun-MIR156a,vun-MIR156b,vvi-MIR156a,vvi-MIR156b,vvi-MIR156c,vvi-MIR156d,vvi-MIR156e,vvi-MIR156f,vvi-MIR156g,vvi-MIR156h,vvi-MIR156i,zma-MIR156a,zma-MIR156b,zma-MIR156c,zma-MIR156d,zma-MIR156e,zma-MIR156f,zma-MIR156g,zma-MIR156h,zma-MIR156i,zma-MIR156j,zma-MIR156k,zma-MIR156l |
| **MIR156-z** | 1 | ahy-MIR156a,ahy-MIR156b,ahy-MIR156c,aly-MIR156a,aly-MIR156b,aly-MIR156c,aly-MIR156d,aly-MIR156e,aly-MIR156f,aly-MIR156g,aly-MIR156h,ama-MIR156,aqc-MIR156a,aqc-MIR156b,ata-MIR156a,ata-MIR156b,ata-MIR156c,ata-MIR156d,ata-MIR156e,ath-MIR156a,ath-MIR156b,ath-MIR156c,ath-MIR156d,ath-MIR156e,ath-MIR156f,ath-MIR156g,ath-MIR156h,ath-MIR156i,ath-MIR156j,atr-MIR156a,atr-MIR156b,atr-MIR156c,atr-MIR156d,bcy-MIR156,bdi-MIR156a,bdi-MIR156b,bdi-MIR156c,bdi-MIR156d,bdi-MIR156e,bdi-MIR156f,bdi-MIR156g,bdi-MIR156h,bdi-MIR156i,bdi-MIR156j,bgy-MIR156,bna-MIR156a,bna-MIR156b,bna-MIR156c,bna-MIR156d,bna-MIR156e,bna-MIR156f,bna-MIR156g,bra-MIR156a,bra-MIR156b,bra-MIR156c,bra-MIR156d,bra-MIR156e,bra-MIR156f,bra-MIR156g,cca-MIR156a,cca-MIR156b,cca-MIR156c,cme-MIR156a,cme-MIR156b,cme-MIR156c,cme-MIR156d,cme-MIR156e,cme-MIR156f,cme-MIR156g,cme-MIR156h,cme-MIR156i,cme-MIR156j,cpa-MIR156a,cpa-MIR156b,cpa-MIR156c,cpa-MIR156d,cpa-MIR156e,cpa-MIR156f,csi-MIR156,ctr-MIR156,dpr-MIR156a,dpr-MIR156b,far-MIR156a,far-MIR156b,ghr-MIR156a,ghr-MIR156b,ghr-MIR156c,ghr-MIR156d,gma-MIR156a,gma-MIR156aa,gma-MIR156ab,gma-MIR156b,gma-MIR156c,gma-MIR156d,gma-MIR156e,gma-MIR156f,gma-MIR156g,gma-MIR156h,gma-MIR156i,gma-MIR156j,gma-MIR156k,gma-MIR156l,gma-MIR156m,gma-MIR156n,gma-MIR156o,gma-MIR156p,gma-MIR156q,gma-MIR156r,gma-MIR156s,gma-MIR156t,gma-MIR156u,gma-MIR156v,gma-MIR156w,gma-MIR156x,gma-MIR156y,gma-MIR156z,han-MIR156a,han-MIR156b,han-MIR156c,har-MIR156a,har-MIR156c,hbr-MIR156,hci-MIR156a,hci-MIR156b,hpa-MIR156a,htu-MIR156a,hvu-MIR156a,hvu-MIR156b,lus-MIR156a,lus-MIR156b,lus-MIR156c,lus-MIR156d,lus-MIR156e,lus-MIR156f,lus-MIR156g,lus-MIR156h,lus-MIR156i,mdm-MIR156a,mdm-MIR156aa,mdm-MIR156ab,mdm-MIR156ac,mdm-MIR156ad,mdm-MIR156ae,mdm-MIR156b,mdm-MIR156c,mdm-MIR156d,mdm-MIR156e,mdm-MIR156f,mdm-MIR156g,mdm-MIR156h,mdm-MIR156i,mdm-MIR156j,mdm-MIR156k,mdm-MIR156l,mdm-MIR156m,mdm-MIR156n,mdm-MIR156o,mdm-MIR156p,mdm-MIR156q,mdm-MIR156r,mdm-MIR156s,mdm-MIR156t,mdm-MIR156u,mdm-MIR156v,mdm-MIR156w,mdm-MIR156x,mdm-MIR156y,mdm-MIR156z,mes-MIR156a,mes-MIR156b,mes-MIR156c,mes-MIR156d,mes-MIR156e,mes-MIR156f,mes-MIR156g,mes-MIR156h,mes-MIR156i,mes-MIR156j,mes-MIR156k,mtr-MIR156a,mtr-MIR156b,mtr-MIR156c,mtr-MIR156d,mtr-MIR156e,mtr-MIR156f,mtr-MIR156g,mtr-MIR156h,mtr-MIR156i,mtr-MIR156j,nta-MIR156a,nta-MIR156b,nta-MIR156c,nta-MIR156d,nta-MIR156e,nta-MIR156f,nta-MIR156g,nta-MIR156h,nta-MIR156i,nta-MIR156j,osa-MIR156a,osa-MIR156b,osa-MIR156c,osa-MIR156d,osa-MIR156e,osa-MIR156f,osa-MIR156g,osa-MIR156h,osa-MIR156i,osa-MIR156j,osa-MIR156k,osa-MIR156l,ppe-MIR156a,ppe-MIR156b,ppe-MIR156c,ppe-MIR156d,ppe-MIR156e,ppe-MIR156f,ppe-MIR156g,ppe-MIR156h,ppe-MIR156i,ppt-MIR156a,ppt-MIR156b,ppt-MIR156c,pta-MIR156a,pta-MIR156b,ptc-MIR156a,ptc-MIR156b,ptc-MIR156c,ptc-MIR156d,ptc-MIR156e,ptc-MIR156f,ptc-MIR156g,ptc-MIR156h,ptc-MIR156i,ptc-MIR156j,ptc-MIR156k,ptc-MIR156l,rco-MIR156a,rco-MIR156b,rco-MIR156c,rco-MIR156d,rco-MIR156e,rco-MIR156f,rco-MIR156g,rco-MIR156h,sbi-MIR156a,sbi-MIR156b,sbi-MIR156c,sbi-MIR156d,sbi-MIR156e,sbi-MIR156f,sbi-MIR156g,sbi-MIR156h,sbi-MIR156i,sly-MIR156a,sly-MIR156b,sly-MIR156c,sly-MIR156d,sly-MIR156e,smo-MIR156a,smo-MIR156b,smo-MIR156c,smo-MIR156d,sof-MIR156,ssl-MIR156,ssp-MIR156,stu-MIR156a,stu-MIR156b,stu-MIR156c,stu-MIR156d,stu-MIR156e,stu-MIR156f,stu-MIR156g,stu-MIR156h,stu-MIR156i,stu-MIR156j,stu-MIR156k,tae-MIR156,tcc-MIR156a,tcc-MIR156b,tcc-MIR156c,tcc-MIR156d,tcc-MIR156e,tcc-MIR156f,tcc-MIR156g,vun-MIR156a,vun-MIR156b,vvi-MIR156a,vvi-MIR156b,vvi-MIR156c,vvi-MIR156d,vvi-MIR156e,vvi-MIR156f,vvi-MIR156g,vvi-MIR156h,vvi-MIR156i,zma-MIR156a,zma-MIR156b,zma-MIR156c,zma-MIR156d,zma-MIR156e,zma-MIR156f,zma-MIR156g,zma-MIR156h,zma-MIR156i,zma-MIR156j,zma-MIR156k,zma-MIR156l |
| **MIR158-y** | 1 | aly-MIR158a,aly-MIR158b,ath-MIR158a,ath-MIR158b,bra-MIR158 |
| **MIR159-x** | 1 | ahy-MIR159,aly-MIR159a,aly-MIR159b,aly-MIR159c,aqc-MIR159,ath-MIR159a,ath-MIR159b,ath-MIR159c,atr-MIR159,bdi-MIR159a,bdi-MIR159b,bdi-MIR159c,bna-MIR159,bra-MIR159a,cme-MIR159a,cme-MIR159b,cpa-MIR159a,cpa-MIR159b,csi-MIR159,far-MIR159,gma-MIR159a,gma-MIR159b,gma-MIR159c,gma-MIR159d,gma-MIR159e,gma-MIR159f,hbr-MIR159a,hbr-MIR159b,htu-MIR159a,hvu-MIR159a,hvu-MIR159b,lus-MIR159a,lus-MIR159b,lus-MIR159c,mdm-MIR159a,mdm-MIR159b,mdm-MIR159c,mes-MIR159a,mes-MIR159b,mes-MIR159c,mes-MIR159d,mtr-MIR159a,mtr-MIR159b,nta-MIR159,osa-MIR159a,osa-MIR159b,osa-MIR159c,osa-MIR159d,osa-MIR159e,osa-MIR159f,pde-MIR159,ppe-MIR159,pta-MIR159a,pta-MIR159b,pta-MIR159c,ptc-MIR159a,ptc-MIR159b,ptc-MIR159c,ptc-MIR159d,ptc-MIR159e,pvu-MIR159a,rco-MIR159,sbi-MIR159a,sbi-MIR159b,sly-MIR159,smo-MIR159,sof-MIR159a,sof-MIR159b,sof-MIR159c,sof-MIR159d,sof-MIR159e,ssp-MIR159a,tae-MIR159a,tae-MIR159b,vvi-MIR159a,vvi-MIR159b,vvi-MIR159c,zma-MIR159a,zma-MIR159b,zma-MIR159c,zma-MIR159d,zma-MIR159e,zma-MIR159f,zma-MIR159g,zma-MIR159h,zma-MIR159i,zma-MIR159j,zma-MIR159k |
| **MIR159-y** | 1 | ahy-MIR159,aly-MIR159a,aly-MIR159b,aly-MIR159c,aqc-MIR159,ath-MIR159a,ath-MIR159b,ath-MIR159c,atr-MIR159,bdi-MIR159a,bdi-MIR159b,bdi-MIR159c,bna-MIR159,bra-MIR159a,cme-MIR159a,cme-MIR159b,cpa-MIR159a,cpa-MIR159b,csi-MIR159,far-MIR159,gma-MIR159a,gma-MIR159b,gma-MIR159c,gma-MIR159d,gma-MIR159e,gma-MIR159f,hbr-MIR159a,hbr-MIR159b,htu-MIR159a,hvu-MIR159a,hvu-MIR159b,lus-MIR159a,lus-MIR159b,lus-MIR159c,mdm-MIR159a,mdm-MIR159b,mdm-MIR159c,mes-MIR159a,mes-MIR159b,mes-MIR159c,mes-MIR159d,mtr-MIR159a,mtr-MIR159b,nta-MIR159,osa-MIR159a,osa-MIR159b,osa-MIR159c,osa-MIR159d,osa-MIR159e,osa-MIR159f,pde-MIR159,ppe-MIR159,pta-MIR159a,pta-MIR159b,pta-MIR159c,ptc-MIR159a,ptc-MIR159b,ptc-MIR159c,ptc-MIR159d,ptc-MIR159e,pvu-MIR159a,rco-MIR159,sbi-MIR159a,sbi-MIR159b,sly-MIR159,smo-MIR159,sof-MIR159a,sof-MIR159b,sof-MIR159c,sof-MIR159d,sof-MIR159e,ssp-MIR159a,tae-MIR159a,tae-MIR159b,vvi-MIR159a,vvi-MIR159b,vvi-MIR159c,zma-MIR159a,zma-MIR159b,zma-MIR159c,zma-MIR159d,zma-MIR159e,zma-MIR159f,zma-MIR159g,zma-MIR159h,zma-MIR159i,zma-MIR159j,zma-MIR159k |
| **MIR160-x** | 1 | aau-MIR160,ahy-MIR160,aly-MIR160a,aly-MIR160b,aly-MIR160c,aqc-MIR160a,aqc-MIR160b,ata-MIR160a,ata-MIR160b,ata-MIR160c,ath-MIR160a,ath-MIR160b,ath-MIR160c,atr-MIR160,bdi-MIR160a,bdi-MIR160b,bdi-MIR160c,bdi-MIR160d,bdi-MIR160e,bdi-MIR160f,bna-MIR160a,bna-MIR160b,bna-MIR160c,bna-MIR160d,bra-MIR160a,cca-MIR160a,cca-MIR160b,cme-MIR160a,cme-MIR160b,cme-MIR160c,cme-MIR160d,cpa-MIR160a,cpa-MIR160b,cpa-MIR160c,cpa-MIR160d,cpa-MIR160e,cpa-MIR160f,csi-MIR160,dpr-MIR160,far-MIR160,ghr-MIR160,gma-MIR160a,gma-MIR160b,gma-MIR160c,gma-MIR160d,gma-MIR160e,gma-MIR160f,han-MIR160a,htu-MIR160a,lus-MIR160a,lus-MIR160b,lus-MIR160c,lus-MIR160d,lus-MIR160e,lus-MIR160f,lus-MIR160g,lus-MIR160h,lus-MIR160i,lus-MIR160j,mdm-MIR160a,mdm-MIR160b,mdm-MIR160c,mdm-MIR160d,mdm-MIR160e,mes-MIR160a,mes-MIR160b,mes-MIR160c,mes-MIR160d,mes-MIR160e,mes-MIR160f,mes-MIR160g,mes-MIR160h,mtr-MIR160a,mtr-MIR160b,mtr-MIR160c,mtr-MIR160d,mtr-MIR160e,mtr-MIR160f,nta-MIR160a,nta-MIR160b,nta-MIR160c,nta-MIR160d,osa-MIR160a,osa-MIR160b,osa-MIR160c,osa-MIR160d,osa-MIR160e,osa-MIR160f,pab-MIR160a,pab-MIR160b,ppe-MIR160a,ppe-MIR160b,ppt-MIR160a,ppt-MIR160b,ppt-MIR160c,ppt-MIR160d,ppt-MIR160e,ppt-MIR160f,ppt-MIR160g,ppt-MIR160h,ppt-MIR160i,ptc-MIR160a,ptc-MIR160b,ptc-MIR160c,ptc-MIR160d,ptc-MIR160e,ptc-MIR160f,ptc-MIR160g,ptc-MIR160h,rco-MIR160a,rco-MIR160b,rco-MIR160c,sbi-MIR160a,sbi-MIR160b,sbi-MIR160c,sbi-MIR160d,sbi-MIR160e,sbi-MIR160f,sly-MIR160a,smo-MIR160a,smo-MIR160b,stu-MIR160a,stu-MIR160b,tae-MIR160,tcc-MIR160a,tcc-MIR160b,tcc-MIR160c,ttu-MIR160,vun-MIR160,vvi-MIR160a,vvi-MIR160b,vvi-MIR160c,vvi-MIR160d,vvi-MIR160e,zma-MIR160a,zma-MIR160b,zma-MIR160c,zma-MIR160d,zma-MIR160e,zma-MIR160f,zma-MIR160g |
| **MIR161-x** | 1 | aly-MIR161,ath-MIR161,bna-MIR161,bra-MIR161 |
| **MIR162-x** | 1 | aau-MIR162,aly-MIR162a,aly-MIR162b,ath-MIR162a,ath-MIR162b,bdi-MIR162,bna-MIR162a,bra-MIR162,cln-MIR162,cme-MIR162,cpa-MIR162a,csi-MIR162,ghr-MIR162a,gma-MIR162a,gma-MIR162b,gma-MIR162c,hpe-MIR162a,htu-MIR162a,lus-MIR162a,lus-MIR162b,mdm-MIR162a,mdm-MIR162b,mes-MIR162,mtr-MIR162,nta-MIR162a,nta-MIR162b,osa-MIR162a,osa-MIR162b,pde-MIR162,ppe-MIR162,ptc-MIR162a,ptc-MIR162b,rco-MIR162,sbi-MIR162,sly-MIR162,stu-MIR162a,stu-MIR162b,tcc-MIR162,vun-MIR162,vvi-MIR162,zma-MIR162 |
| **MIR162-y** | 1 | aau-MIR162,aly-MIR162a,aly-MIR162b,ath-MIR162a,ath-MIR162b,bdi-MIR162,bna-MIR162a,bra-MIR162,cln-MIR162,cme-MIR162,cpa-MIR162a,csi-MIR162,ghr-MIR162a,gma-MIR162a,gma-MIR162b,gma-MIR162c,hpe-MIR162a,htu-MIR162a,lus-MIR162a,lus-MIR162b,mdm-MIR162a,mdm-MIR162b,mes-MIR162,mtr-MIR162,nta-MIR162a,nta-MIR162b,osa-MIR162a,osa-MIR162b,pde-MIR162,ppe-MIR162,ptc-MIR162a,ptc-MIR162b,rco-MIR162,sbi-MIR162,sly-MIR162,stu-MIR162a,stu-MIR162b,tcc-MIR162,vun-MIR162,vvi-MIR162,zma-MIR162 |
| **MIR163-y** | 1 | aly-MIR163,ath-MIR163 |
| **MIR164-x** | 1 | aly-MIR164a,aly-MIR164b,aly-MIR164c,ata-MIR164a,ata-MIR164b,ata-MIR164c,ath-MIR164a,ath-MIR164b,ath-MIR164c,atr-MIR164a,atr-MIR164b,bdi-MIR164a,bdi-MIR164b,bdi-MIR164c,bdi-MIR164e,bdi-MIR164f,bna-MIR164a,bna-MIR164b,bna-MIR164c,bna-MIR164d,bra-MIR164a,bra-MIR164b,bra-MIR164c,bra-MIR164d,bra-MIR164e,cca-MIR164,cln-MIR164,cme-MIR164a,cme-MIR164b,cme-MIR164c,cme-MIR164d,cpa-MIR164a,cpa-MIR164b,cpa-MIR164c,cpa-MIR164d,cpa-MIR164e,csi-MIR164,ctr-MIR164,far-MIR164a,far-MIR164b,ghr-MIR164,gma-MIR164a,gma-MIR164b,gma-MIR164c,gma-MIR164d,gma-MIR164e,gma-MIR164f,gma-MIR164g,gma-MIR164h,gma-MIR164i,gma-MIR164j,gma-MIR164k,gra-MIR164a,hci-MIR164a,lus-MIR164a,lus-MIR164b,lus-MIR164c,lus-MIR164d,lus-MIR164e,mdm-MIR164a,mdm-MIR164b,mdm-MIR164c,mdm-MIR164d,mdm-MIR164e,mdm-MIR164f,mes-MIR164a,mes-MIR164b,mes-MIR164c,mes-MIR164d,mtr-MIR164a,mtr-MIR164b,mtr-MIR164c,mtr-MIR164d,nta-MIR164a,nta-MIR164b,nta-MIR164c,osa-MIR164a,osa-MIR164b,osa-MIR164c,osa-MIR164d,osa-MIR164e,osa-MIR164f,ppe-MIR164a,ppe-MIR164b,ppe-MIR164c,ppe-MIR164d,ptc-MIR164a,ptc-MIR164b,ptc-MIR164c,ptc-MIR164d,ptc-MIR164e,ptc-MIR164f,rco-MIR164a,rco-MIR164b,rco-MIR164c,rco-MIR164d,rgl-MIR164,sbi-MIR164a,sbi-MIR164b,sbi-MIR164c,sbi-MIR164d,sbi-MIR164e,sly-MIR164a,sly-MIR164b,ssl-MIR164a,ssl-MIR164b,stu-MIR164,tae-MIR164,tcc-MIR164a,tcc-MIR164b,tcc-MIR164c,vun-MIR164,vvi-MIR164a,vvi-MIR164b,vvi-MIR164c,vvi-MIR164d,zma-MIR164a,zma-MIR164b,zma-MIR164c,zma-MIR164d,zma-MIR164e,zma-MIR164f,zma-MIR164g,zma-MIR164h |
| **MIR164-y** | 1 | aly-MIR164a,aly-MIR164b,aly-MIR164c,ata-MIR164a,ata-MIR164b,ata-MIR164c,ath-MIR164a,ath-MIR164b,ath-MIR164c,atr-MIR164a,atr-MIR164b,bdi-MIR164a,bdi-MIR164b,bdi-MIR164c,bdi-MIR164e,bdi-MIR164f,bna-MIR164a,bna-MIR164b,bna-MIR164c,bna-MIR164d,bra-MIR164a,bra-MIR164b,bra-MIR164c,bra-MIR164d,bra-MIR164e,cca-MIR164,cln-MIR164,cme-MIR164a,cme-MIR164b,cme-MIR164c,cme-MIR164d,cpa-MIR164a,cpa-MIR164b,cpa-MIR164c,cpa-MIR164d,cpa-MIR164e,csi-MIR164,ctr-MIR164,far-MIR164a,far-MIR164b,ghr-MIR164,gma-MIR164a,gma-MIR164b,gma-MIR164c,gma-MIR164d,gma-MIR164e,gma-MIR164f,gma-MIR164g,gma-MIR164h,gma-MIR164i,gma-MIR164j,gma-MIR164k,gra-MIR164a,hci-MIR164a,lus-MIR164a,lus-MIR164b,lus-MIR164c,lus-MIR164d,lus-MIR164e,mdm-MIR164a,mdm-MIR164b,mdm-MIR164c,mdm-MIR164d,mdm-MIR164e,mdm-MIR164f,mes-MIR164a,mes-MIR164b,mes-MIR164c,mes-MIR164d,mtr-MIR164a,mtr-MIR164b,mtr-MIR164c,mtr-MIR164d,nta-MIR164a,nta-MIR164b,nta-MIR164c,osa-MIR164a,osa-MIR164b,osa-MIR164c,osa-MIR164d,osa-MIR164e,osa-MIR164f,ppe-MIR164a,ppe-MIR164b,ppe-MIR164c,ppe-MIR164d,ptc-MIR164a,ptc-MIR164b,ptc-MIR164c,ptc-MIR164d,ptc-MIR164e,ptc-MIR164f,rco-MIR164a,rco-MIR164b,rco-MIR164c,rco-MIR164d,rgl-MIR164,sbi-MIR164a,sbi-MIR164b,sbi-MIR164c,sbi-MIR164d,sbi-MIR164e,sly-MIR164a,sly-MIR164b,ssl-MIR164a,ssl-MIR164b,stu-MIR164,tae-MIR164,tcc-MIR164a,tcc-MIR164b,tcc-MIR164c,vun-MIR164,vvi-MIR164a,vvi-MIR164b,vvi-MIR164c,vvi-MIR164d,zma-MIR164a,zma-MIR164b,zma-MIR164c,zma-MIR164d,zma-MIR164e,zma-MIR164f,zma-MIR164g,zma-MIR164h |
| **MIR165-y** | 1 | aly-MIR165a,aly-MIR165b,ath-MIR165a,ath-MIR165b |
| **MIR166-x** | 1 | aly-MIR166a,aly-MIR166b,aly-MIR166c,aly-MIR166d,aly-MIR166e,aly-MIR166f,aly-MIR166g,aly-MIR166h,aqc-MIR166a,aqc-MIR166b,aqc-MIR166c,aqc-MIR166d,aqc-MIR166e,ata-MIR166a,ata-MIR166b,ata-MIR166c,ata-MIR166d,ata-MIR166e,ath-MIR166a,ath-MIR166b,ath-MIR166c,ath-MIR166d,ath-MIR166e,ath-MIR166f,ath-MIR166g,atr-MIR166a,atr-MIR166b,atr-MIR166c,atr-MIR166d,bdi-MIR166a,bdi-MIR166b,bdi-MIR166c,bdi-MIR166d,bdi-MIR166e,bdi-MIR166f,bdi-MIR166g,bdi-MIR166h,bdi-MIR166i,bdi-MIR166j,bna-MIR166a,bna-MIR166b,bna-MIR166c,bna-MIR166d,bna-MIR166e,bna-MIR166f,cln-MIR166,cme-MIR166a,cme-MIR166b,cme-MIR166c,cme-MIR166d,cme-MIR166e,cme-MIR166f,cme-MIR166g,cme-MIR166h,cme-MIR166i,cpa-MIR166a,cpa-MIR166b,cpa-MIR166c,cpa-MIR166d,cpa-MIR166e,crt-MIR166a,crt-MIR166b,csi-MIR166a,csi-MIR166b,csi-MIR166c,csi-MIR166d,csi-MIR166e,ctr-MIR166,dpr-MIR166a,dpr-MIR166b,far-MIR166,ghr-MIR166b,gma-MIR166a,gma-MIR166b,gma-MIR166c,gma-MIR166d,gma-MIR166e,gma-MIR166f,gma-MIR166g,gma-MIR166h,gma-MIR166i,gma-MIR166j,gma-MIR166k,gma-MIR166l,gma-MIR166m,gma-MIR166n,gma-MIR166o,gma-MIR166p,gma-MIR166q,gma-MIR166r,gma-MIR166s,gma-MIR166t,gma-MIR166u,gra-MIR166c,gra-MIR166d,hbr-MIR166a,hbr-MIR166b,hpa-MIR166a,hpe-MIR166a,hvu-MIR166a,hvu-MIR166b,hvu-MIR166c,lus-MIR166a,lus-MIR166b,lus-MIR166c,lus-MIR166d,lus-MIR166e,lus-MIR166f,lus-MIR166g,lus-MIR166h,lus-MIR166i,lus-MIR166j,lus-MIR166k,mdm-MIR166a,mdm-MIR166b,mdm-MIR166c,mdm-MIR166d,mdm-MIR166e,mdm-MIR166f,mdm-MIR166g,mdm-MIR166h,mdm-MIR166i,mes-MIR166a,mes-MIR166b,mes-MIR166c,mes-MIR166d,mes-MIR166e,mes-MIR166f,mes-MIR166g,mes-MIR166h,mes-MIR166i,mes-MIR166j,mtr-MIR166a,mtr-MIR166b,mtr-MIR166c,mtr-MIR166d,mtr-MIR166e,mtr-MIR166f,mtr-MIR166g,nta-MIR166a,nta-MIR166b,nta-MIR166c,nta-MIR166d,nta-MIR166e,nta-MIR166f,nta-MIR166g,nta-MIR166h,osa-MIR166a,osa-MIR166b,osa-MIR166c,osa-MIR166d,osa-MIR166e,osa-MIR166f,osa-MIR166g,osa-MIR166h,osa-MIR166i,osa-MIR166j,osa-MIR166k,osa-MIR166l,osa-MIR166m,pab-MIR166a,pab-MIR166b,pde-MIR166a,pde-MIR166b,ppe-MIR166a,ppe-MIR166b,ppe-MIR166c,ppe-MIR166d,ppe-MIR166e,ppt-MIR166a,ppt-MIR166b,ppt-MIR166c,ppt-MIR166d,ppt-MIR166e,ppt-MIR166f,ppt-MIR166g,ppt-MIR166h,ppt-MIR166i,ppt-MIR166j,ppt-MIR166k,ppt-MIR166l,ppt-MIR166m,pta-MIR166a,pta-MIR166b,pta-MIR166c,ptc-MIR166a,ptc-MIR166b,ptc-MIR166c,ptc-MIR166d,ptc-MIR166e,ptc-MIR166f,ptc-MIR166g,ptc-MIR166h,ptc-MIR166i,ptc-MIR166j,ptc-MIR166k,ptc-MIR166l,ptc-MIR166m,ptc-MIR166n,ptc-MIR166o,ptc-MIR166p,ptc-MIR166q,pvu-MIR166a,rco-MIR166a,rco-MIR166b,rco-MIR166c,rco-MIR166d,rco-MIR166e,sbi-MIR166a,sbi-MIR166b,sbi-MIR166c,sbi-MIR166d,sbi-MIR166e,sbi-MIR166f,sbi-MIR166g,sbi-MIR166h,sbi-MIR166i,sbi-MIR166j,sbi-MIR166k,sly-MIR166a,sly-MIR166b,sly-MIR166c,smo-MIR166a,smo-MIR166b,smo-MIR166c,ssl-MIR166a,ssl-MIR166b,ssp-MIR166,stu-MIR166a,stu-MIR166b,stu-MIR166c,stu-MIR166d,tcc-MIR166a,tcc-MIR166b,tcc-MIR166c,tcc-MIR166d,vvi-MIR166a,vvi-MIR166b,vvi-MIR166c,vvi-MIR166d,vvi-MIR166e,vvi-MIR166f,vvi-MIR166g,vvi-MIR166h,zma-MIR166a,zma-MIR166b,zma-MIR166c,zma-MIR166d,zma-MIR166e,zma-MIR166f,zma-MIR166g,zma-MIR166h,zma-MIR166i,zma-MIR166j,zma-MIR166k,zma-MIR166l,zma-MIR166m,zma-MIR166n |
| **MIR166-y** | 1 | aly-MIR166a,aly-MIR166b,aly-MIR166c,aly-MIR166d,aly-MIR166e,aly-MIR166f,aly-MIR166g,aly-MIR166h,aqc-MIR166a,aqc-MIR166b,aqc-MIR166c,aqc-MIR166d,aqc-MIR166e,ata-MIR166a,ata-MIR166b,ata-MIR166c,ata-MIR166d,ata-MIR166e,ath-MIR166a,ath-MIR166b,ath-MIR166c,ath-MIR166d,ath-MIR166e,ath-MIR166f,ath-MIR166g,atr-MIR166a,atr-MIR166b,atr-MIR166c,atr-MIR166d,bdi-MIR166a,bdi-MIR166b,bdi-MIR166c,bdi-MIR166d,bdi-MIR166e,bdi-MIR166f,bdi-MIR166g,bdi-MIR166h,bdi-MIR166i,bdi-MIR166j,bna-MIR166a,bna-MIR166b,bna-MIR166c,bna-MIR166d,bna-MIR166e,bna-MIR166f,cln-MIR166,cme-MIR166a,cme-MIR166b,cme-MIR166c,cme-MIR166d,cme-MIR166e,cme-MIR166f,cme-MIR166g,cme-MIR166h,cme-MIR166i,cpa-MIR166a,cpa-MIR166b,cpa-MIR166c,cpa-MIR166d,cpa-MIR166e,crt-MIR166a,crt-MIR166b,csi-MIR166a,csi-MIR166b,csi-MIR166c,csi-MIR166d,csi-MIR166e,ctr-MIR166,dpr-MIR166a,dpr-MIR166b,far-MIR166,ghr-MIR166b,gma-MIR166a,gma-MIR166b,gma-MIR166c,gma-MIR166d,gma-MIR166e,gma-MIR166f,gma-MIR166g,gma-MIR166h,gma-MIR166i,gma-MIR166j,gma-MIR166k,gma-MIR166l,gma-MIR166m,gma-MIR166n,gma-MIR166o,gma-MIR166p,gma-MIR166q,gma-MIR166r,gma-MIR166s,gma-MIR166t,gma-MIR166u,gra-MIR166c,gra-MIR166d,hbr-MIR166a,hbr-MIR166b,hpa-MIR166a,hpe-MIR166a,hvu-MIR166a,hvu-MIR166b,hvu-MIR166c,lus-MIR166a,lus-MIR166b,lus-MIR166c,lus-MIR166d,lus-MIR166e,lus-MIR166f,lus-MIR166g,lus-MIR166h,lus-MIR166i,lus-MIR166j,lus-MIR166k,mdm-MIR166a,mdm-MIR166b,mdm-MIR166c,mdm-MIR166d,mdm-MIR166e,mdm-MIR166f,mdm-MIR166g,mdm-MIR166h,mdm-MIR166i,mes-MIR166a,mes-MIR166b,mes-MIR166c,mes-MIR166d,mes-MIR166e,mes-MIR166f,mes-MIR166g,mes-MIR166h,mes-MIR166i,mes-MIR166j,mtr-MIR166a,mtr-MIR166b,mtr-MIR166c,mtr-MIR166d,mtr-MIR166e,mtr-MIR166f,mtr-MIR166g,nta-MIR166a,nta-MIR166b,nta-MIR166c,nta-MIR166d,nta-MIR166e,nta-MIR166f,nta-MIR166g,nta-MIR166h,osa-MIR166a,osa-MIR166b,osa-MIR166c,osa-MIR166d,osa-MIR166e,osa-MIR166f,osa-MIR166g,osa-MIR166h,osa-MIR166i,osa-MIR166j,osa-MIR166k,osa-MIR166l,osa-MIR166m,pab-MIR166a,pab-MIR166b,pde-MIR166a,pde-MIR166b,ppe-MIR166a,ppe-MIR166b,ppe-MIR166c,ppe-MIR166d,ppe-MIR166e,ppt-MIR166a,ppt-MIR166b,ppt-MIR166c,ppt-MIR166d,ppt-MIR166e,ppt-MIR166f,ppt-MIR166g,ppt-MIR166h,ppt-MIR166i,ppt-MIR166j,ppt-MIR166k,ppt-MIR166l,ppt-MIR166m,pta-MIR166a,pta-MIR166b,pta-MIR166c,ptc-MIR166a,ptc-MIR166b,ptc-MIR166c,ptc-MIR166d,ptc-MIR166e,ptc-MIR166f,ptc-MIR166g,ptc-MIR166h,ptc-MIR166i,ptc-MIR166j,ptc-MIR166k,ptc-MIR166l,ptc-MIR166m,ptc-MIR166n,ptc-MIR166o,ptc-MIR166p,ptc-MIR166q,pvu-MIR166a,rco-MIR166a,rco-MIR166b,rco-MIR166c,rco-MIR166d,rco-MIR166e,sbi-MIR166a,sbi-MIR166b,sbi-MIR166c,sbi-MIR166d,sbi-MIR166e,sbi-MIR166f,sbi-MIR166g,sbi-MIR166h,sbi-MIR166i,sbi-MIR166j,sbi-MIR166k,sly-MIR166a,sly-MIR166b,sly-MIR166c,smo-MIR166a,smo-MIR166b,smo-MIR166c,ssl-MIR166a,ssl-MIR166b,ssp-MIR166,stu-MIR166a,stu-MIR166b,stu-MIR166c,stu-MIR166d,tcc-MIR166a,tcc-MIR166b,tcc-MIR166c,tcc-MIR166d,vvi-MIR166a,vvi-MIR166b,vvi-MIR166c,vvi-MIR166d,vvi-MIR166e,vvi-MIR166f,vvi-MIR166g,vvi-MIR166h,zma-MIR166a,zma-MIR166b,zma-MIR166c,zma-MIR166d,zma-MIR166e,zma-MIR166f,zma-MIR166g,zma-MIR166h,zma-MIR166i,zma-MIR166j,zma-MIR166k,zma-MIR166l,zma-MIR166m,zma-MIR166n |
| **MIR166-z** | 1 | aly-MIR166a,aly-MIR166b,aly-MIR166c,aly-MIR166d,aly-MIR166e,aly-MIR166f,aly-MIR166g,aly-MIR166h,aqc-MIR166a,aqc-MIR166b,aqc-MIR166c,aqc-MIR166d,aqc-MIR166e,ata-MIR166a,ata-MIR166b,ata-MIR166c,ata-MIR166d,ata-MIR166e,ath-MIR166a,ath-MIR166b,ath-MIR166c,ath-MIR166d,ath-MIR166e,ath-MIR166f,ath-MIR166g,atr-MIR166a,atr-MIR166b,atr-MIR166c,atr-MIR166d,bdi-MIR166a,bdi-MIR166b,bdi-MIR166c,bdi-MIR166d,bdi-MIR166e,bdi-MIR166f,bdi-MIR166g,bdi-MIR166h,bdi-MIR166i,bdi-MIR166j,bna-MIR166a,bna-MIR166b,bna-MIR166c,bna-MIR166d,bna-MIR166e,bna-MIR166f,cln-MIR166,cme-MIR166a,cme-MIR166b,cme-MIR166c,cme-MIR166d,cme-MIR166e,cme-MIR166f,cme-MIR166g,cme-MIR166h,cme-MIR166i,cpa-MIR166a,cpa-MIR166b,cpa-MIR166c,cpa-MIR166d,cpa-MIR166e,crt-MIR166a,crt-MIR166b,csi-MIR166a,csi-MIR166b,csi-MIR166c,csi-MIR166d,csi-MIR166e,ctr-MIR166,dpr-MIR166a,dpr-MIR166b,far-MIR166,ghr-MIR166b,gma-MIR166a,gma-MIR166b,gma-MIR166c,gma-MIR166d,gma-MIR166e,gma-MIR166f,gma-MIR166g,gma-MIR166h,gma-MIR166i,gma-MIR166j,gma-MIR166k,gma-MIR166l,gma-MIR166m,gma-MIR166n,gma-MIR166o,gma-MIR166p,gma-MIR166q,gma-MIR166r,gma-MIR166s,gma-MIR166t,gma-MIR166u,gra-MIR166c,gra-MIR166d,hbr-MIR166a,hbr-MIR166b,hpa-MIR166a,hpe-MIR166a,hvu-MIR166a,hvu-MIR166b,hvu-MIR166c,lus-MIR166a,lus-MIR166b,lus-MIR166c,lus-MIR166d,lus-MIR166e,lus-MIR166f,lus-MIR166g,lus-MIR166h,lus-MIR166i,lus-MIR166j,lus-MIR166k,mdm-MIR166a,mdm-MIR166b,mdm-MIR166c,mdm-MIR166d,mdm-MIR166e,mdm-MIR166f,mdm-MIR166g,mdm-MIR166h,mdm-MIR166i,mes-MIR166a,mes-MIR166b,mes-MIR166c,mes-MIR166d,mes-MIR166e,mes-MIR166f,mes-MIR166g,mes-MIR166h,mes-MIR166i,mes-MIR166j,mtr-MIR166a,mtr-MIR166b,mtr-MIR166c,mtr-MIR166d,mtr-MIR166e,mtr-MIR166f,mtr-MIR166g,nta-MIR166a,nta-MIR166b,nta-MIR166c,nta-MIR166d,nta-MIR166e,nta-MIR166f,nta-MIR166g,nta-MIR166h,osa-MIR166a,osa-MIR166b,osa-MIR166c,osa-MIR166d,osa-MIR166e,osa-MIR166f,osa-MIR166g,osa-MIR166h,osa-MIR166i,osa-MIR166j,osa-MIR166k,osa-MIR166l,osa-MIR166m,pab-MIR166a,pab-MIR166b,pde-MIR166a,pde-MIR166b,ppe-MIR166a,ppe-MIR166b,ppe-MIR166c,ppe-MIR166d,ppe-MIR166e,ppt-MIR166a,ppt-MIR166b,ppt-MIR166c,ppt-MIR166d,ppt-MIR166e,ppt-MIR166f,ppt-MIR166g,ppt-MIR166h,ppt-MIR166i,ppt-MIR166j,ppt-MIR166k,ppt-MIR166l,ppt-MIR166m,pta-MIR166a,pta-MIR166b,pta-MIR166c,ptc-MIR166a,ptc-MIR166b,ptc-MIR166c,ptc-MIR166d,ptc-MIR166e,ptc-MIR166f,ptc-MIR166g,ptc-MIR166h,ptc-MIR166i,ptc-MIR166j,ptc-MIR166k,ptc-MIR166l,ptc-MIR166m,ptc-MIR166n,ptc-MIR166o,ptc-MIR166p,ptc-MIR166q,pvu-MIR166a,rco-MIR166a,rco-MIR166b,rco-MIR166c,rco-MIR166d,rco-MIR166e,sbi-MIR166a,sbi-MIR166b,sbi-MIR166c,sbi-MIR166d,sbi-MIR166e,sbi-MIR166f,sbi-MIR166g,sbi-MIR166h,sbi-MIR166i,sbi-MIR166j,sbi-MIR166k,sly-MIR166a,sly-MIR166b,sly-MIR166c,smo-MIR166a,smo-MIR166b,smo-MIR166c,ssl-MIR166a,ssl-MIR166b,ssp-MIR166,stu-MIR166a,stu-MIR166b,stu-MIR166c,stu-MIR166d,tcc-MIR166a,tcc-MIR166b,tcc-MIR166c,tcc-MIR166d,vvi-MIR166a,vvi-MIR166b,vvi-MIR166c,vvi-MIR166d,vvi-MIR166e,vvi-MIR166f,vvi-MIR166g,vvi-MIR166h,zma-MIR166a,zma-MIR166b,zma-MIR166c,zma-MIR166d,zma-MIR166e,zma-MIR166f,zma-MIR166g,zma-MIR166h,zma-MIR166i,zma-MIR166j,zma-MIR166k,zma-MIR166l,zma-MIR166m,zma-MIR166n |
| **MIR167-x** | 1 | ahy-MIR167,aly-MIR167a,aly-MIR167b,aly-MIR167c,aly-MIR167d,aqc-MIR167,ata-MIR167a,ata-MIR167b,ata-MIR167c,ata-MIR167d,ata-MIR167e,ata-MIR167f,ath-MIR167a,ath-MIR167b,ath-MIR167c,ath-MIR167d,atr-MIR167,bdi-MIR167a,bdi-MIR167b,bdi-MIR167c,bdi-MIR167d,bdi-MIR167e,bdi-MIR167f,bdi-MIR167g,bna-MIR167a,bna-MIR167b,bna-MIR167c,bna-MIR167d,bra-MIR167a,bra-MIR167b,bra-MIR167c,bra-MIR167d,cca-MIR167,ccl-MIR167a,ccl-MIR167b,cme-MIR167a,cme-MIR167b,cme-MIR167c,cme-MIR167d,cme-MIR167e,cme-MIR167f,cpa-MIR167a,cpa-MIR167b,cpa-MIR167c,cpa-MIR167d,csi-MIR167a,csi-MIR167b,csi-MIR167c,ctr-MIR167,dpr-MIR167a,dpr-MIR167b,dpr-MIR167c,ghr-MIR167a,ghr-MIR167b,gma-MIR167a,gma-MIR167b,gma-MIR167c,gma-MIR167d,gma-MIR167e,gma-MIR167f,gma-MIR167g,gma-MIR167h,gma-MIR167i,gma-MIR167j,gma-MIR167k,gra-MIR167a,gra-MIR167b,gra-MIR167c,gso-MIR167a,lja-MIR167,lja-MIR167a,lja-MIR167b,lja-MIR167c,lus-MIR167a,lus-MIR167b,lus-MIR167c,lus-MIR167d,lus-MIR167e,lus-MIR167f,lus-MIR167g,lus-MIR167h,lus-MIR167i,mdm-MIR167a,mdm-MIR167b,mdm-MIR167c,mdm-MIR167d,mdm-MIR167e,mdm-MIR167f,mdm-MIR167g,mdm-MIR167h,mdm-MIR167i,mdm-MIR167j,mes-MIR167a,mes-MIR167b,mes-MIR167c,mes-MIR167d,mes-MIR167e,mes-MIR167f,mes-MIR167g,mes-MIR167h,mtr-MIR167a,mtr-MIR167b,nta-MIR167a,nta-MIR167b,nta-MIR167c,nta-MIR167d,nta-MIR167e,osa-MIR167a,osa-MIR167b,osa-MIR167c,osa-MIR167d,osa-MIR167e,osa-MIR167f,osa-MIR167g,osa-MIR167h,osa-MIR167i,osa-MIR167j,ppe-MIR167a,ppe-MIR167b,ppe-MIR167c,ppe-MIR167d,ppt-MIR167,ptc-MIR167a,ptc-MIR167b,ptc-MIR167c,ptc-MIR167d,ptc-MIR167e,ptc-MIR167f,ptc-MIR167g,ptc-MIR167h,rco-MIR167a,rco-MIR167b,rco-MIR167c,sbi-MIR167a,sbi-MIR167b,sbi-MIR167c,sbi-MIR167d,sbi-MIR167e,sbi-MIR167f,sbi-MIR167g,sbi-MIR167h,sbi-MIR167i,sly-MIR167a,sly-MIR167b,sof-MIR167a,sof-MIR167b,ssp-MIR167b,stu-MIR167a,stu-MIR167b,stu-MIR167c,stu-MIR167d,tae-MIR167a,tae-MIR167b,tae-MIR167c,tcc-MIR167a,tcc-MIR167b,tcc-MIR167c,vvi-MIR167a,vvi-MIR167b,vvi-MIR167c,vvi-MIR167d,vvi-MIR167e,zma-MIR167a,zma-MIR167b,zma-MIR167c,zma-MIR167d,zma-MIR167e,zma-MIR167f,zma-MIR167g,zma-MIR167h,zma-MIR167i,zma-MIR167j |
| **MIR167-y** | 1 | ahy-MIR167,aly-MIR167a,aly-MIR167b,aly-MIR167c,aly-MIR167d,aqc-MIR167,ata-MIR167a,ata-MIR167b,ata-MIR167c,ata-MIR167d,ata-MIR167e,ata-MIR167f,ath-MIR167a,ath-MIR167b,ath-MIR167c,ath-MIR167d,atr-MIR167,bdi-MIR167a,bdi-MIR167b,bdi-MIR167c,bdi-MIR167d,bdi-MIR167e,bdi-MIR167f,bdi-MIR167g,bna-MIR167a,bna-MIR167b,bna-MIR167c,bna-MIR167d,bra-MIR167a,bra-MIR167b,bra-MIR167c,bra-MIR167d,cca-MIR167,ccl-MIR167a,ccl-MIR167b,cme-MIR167a,cme-MIR167b,cme-MIR167c,cme-MIR167d,cme-MIR167e,cme-MIR167f,cpa-MIR167a,cpa-MIR167b,cpa-MIR167c,cpa-MIR167d,csi-MIR167a,csi-MIR167b,csi-MIR167c,ctr-MIR167,dpr-MIR167a,dpr-MIR167b,dpr-MIR167c,ghr-MIR167a,ghr-MIR167b,gma-MIR167a,gma-MIR167b,gma-MIR167c,gma-MIR167d,gma-MIR167e,gma-MIR167f,gma-MIR167g,gma-MIR167h,gma-MIR167i,gma-MIR167j,gma-MIR167k,gra-MIR167a,gra-MIR167b,gra-MIR167c,gso-MIR167a,lja-MIR167,lja-MIR167a,lja-MIR167b,lja-MIR167c,lus-MIR167a,lus-MIR167b,lus-MIR167c,lus-MIR167d,lus-MIR167e,lus-MIR167f,lus-MIR167g,lus-MIR167h,lus-MIR167i,mdm-MIR167a,mdm-MIR167b,mdm-MIR167c,mdm-MIR167d,mdm-MIR167e,mdm-MIR167f,mdm-MIR167g,mdm-MIR167h,mdm-MIR167i,mdm-MIR167j,mes-MIR167a,mes-MIR167b,mes-MIR167c,mes-MIR167d,mes-MIR167e,mes-MIR167f,mes-MIR167g,mes-MIR167h,mtr-MIR167a,mtr-MIR167b,nta-MIR167a,nta-MIR167b,nta-MIR167c,nta-MIR167d,nta-MIR167e,osa-MIR167a,osa-MIR167b,osa-MIR167c,osa-MIR167d,osa-MIR167e,osa-MIR167f,osa-MIR167g,osa-MIR167h,osa-MIR167i,osa-MIR167j,ppe-MIR167a,ppe-MIR167b,ppe-MIR167c,ppe-MIR167d,ppt-MIR167,ptc-MIR167a,ptc-MIR167b,ptc-MIR167c,ptc-MIR167d,ptc-MIR167e,ptc-MIR167f,ptc-MIR167g,ptc-MIR167h,rco-MIR167a,rco-MIR167b,rco-MIR167c,sbi-MIR167a,sbi-MIR167b,sbi-MIR167c,sbi-MIR167d,sbi-MIR167e,sbi-MIR167f,sbi-MIR167g,sbi-MIR167h,sbi-MIR167i,sly-MIR167a,sly-MIR167b,sof-MIR167a,sof-MIR167b,ssp-MIR167b,stu-MIR167a,stu-MIR167b,stu-MIR167c,stu-MIR167d,tae-MIR167a,tae-MIR167b,tae-MIR167c,tcc-MIR167a,tcc-MIR167b,tcc-MIR167c,vvi-MIR167a,vvi-MIR167b,vvi-MIR167c,vvi-MIR167d,vvi-MIR167e,zma-MIR167a,zma-MIR167b,zma-MIR167c,zma-MIR167d,zma-MIR167e,zma-MIR167f,zma-MIR167g,zma-MIR167h,zma-MIR167i,zma-MIR167j |
| **MIR168-x** | 1 | aau-MIR168,aly-MIR168a,aly-MIR168b,aqc-MIR168,ata-MIR168,ath-MIR168a,ath-MIR168b,atr-MIR168,bdi-MIR168,bna-MIR168a,bna-MIR168b,bra-MIR168a,bra-MIR168b,bra-MIR168c,cca-MIR168a,ccl-MIR168,cme-MIR168,crt-MIR168,gma-MIR168a,gma-MIR168b,hvu-MIR168,lus-MIR168a,lus-MIR168b,mdm-MIR168a,mdm-MIR168b,mes-MIR168a,mtr-MIR168a,mtr-MIR168b,mtr-MIR168c,nta-MIR168a,nta-MIR168b,nta-MIR168c,nta-MIR168d,nta-MIR168e,osa-MIR168a,osa-MIR168b,ppe-MIR168,ptc-MIR168a,ptc-MIR168b,rco-MIR168,sbi-MIR168,sly-MIR168a,sly-MIR168b,sof-MIR168a,sof-MIR168b,ssp-MIR168a,tcc-MIR168,vun-MIR168,vvi-MIR168,zma-MIR168a,zma-MIR168b |
| **MIR168-y** | 1 | aau-MIR168,aly-MIR168a,aly-MIR168b,aqc-MIR168,ata-MIR168,ath-MIR168a,ath-MIR168b,atr-MIR168,bdi-MIR168,bna-MIR168a,bna-MIR168b,bra-MIR168a,bra-MIR168b,bra-MIR168c,cca-MIR168a,ccl-MIR168,cme-MIR168,crt-MIR168,gma-MIR168a,gma-MIR168b,hvu-MIR168,lus-MIR168a,lus-MIR168b,mdm-MIR168a,mdm-MIR168b,mes-MIR168a,mtr-MIR168a,mtr-MIR168b,mtr-MIR168c,nta-MIR168a,nta-MIR168b,nta-MIR168c,nta-MIR168d,nta-MIR168e,osa-MIR168a,osa-MIR168b,ppe-MIR168,ptc-MIR168a,ptc-MIR168b,rco-MIR168,sbi-MIR168,sly-MIR168a,sly-MIR168b,sof-MIR168a,sof-MIR168b,ssp-MIR168a,tcc-MIR168,vun-MIR168,vvi-MIR168,zma-MIR168a,zma-MIR168b |
| **MIR169-x** | 1 | aly-MIR169a,aly-MIR169b,aly-MIR169c,aly-MIR169d,aly-MIR169e,aly-MIR169f,aly-MIR169g,aly-MIR169h,aly-MIR169i,aly-MIR169j,aly-MIR169k,aly-MIR169l,aly-MIR169m,aly-MIR169n,aqc-MIR169a,aqc-MIR169b,aqc-MIR169c,ata-MIR169a,ata-MIR169b,ata-MIR169c,ata-MIR169d,ata-MIR169e,ata-MIR169f,ata-MIR169g,ata-MIR169h,ata-MIR169i,ata-MIR169j,ath-MIR169a,ath-MIR169b,ath-MIR169c,ath-MIR169d,ath-MIR169e,ath-MIR169f,ath-MIR169g,ath-MIR169h,ath-MIR169i,ath-MIR169j,ath-MIR169k,ath-MIR169l,ath-MIR169m,ath-MIR169n,atr-MIR169a,atr-MIR169b,atr-MIR169c,bdi-MIR169a,bdi-MIR169b,bdi-MIR169c,bdi-MIR169d,bdi-MIR169e,bdi-MIR169f,bdi-MIR169g,bdi-MIR169h,bdi-MIR169i,bdi-MIR169j,bdi-MIR169k,bdi-MIR169l,bdi-MIR169m,bdi-MIR169n,bna-MIR169a,bna-MIR169b,bna-MIR169c,bna-MIR169d,bna-MIR169e,bna-MIR169f,bna-MIR169g,bna-MIR169h,bna-MIR169i,bna-MIR169j,bna-MIR169k,bna-MIR169l,bna-MIR169m,bna-MIR169n,cca-MIR169a,cca-MIR169b,cme-MIR169a,cme-MIR169b,cme-MIR169c,cme-MIR169d,cme-MIR169e,cme-MIR169f,cme-MIR169g,cme-MIR169h,cme-MIR169i,cme-MIR169j,cme-MIR169k,cme-MIR169l,cme-MIR169m,cme-MIR169n,cme-MIR169o,cme-MIR169p,cme-MIR169q,cme-MIR169r,cme-MIR169s,cme-MIR169t,cpa-MIR169,csi-MIR169,far-MIR169,ghb-MIR169a,ghr-MIR169a,ghr-MIR169b,gma-MIR169a,gma-MIR169b,gma-MIR169c,gma-MIR169d,gma-MIR169e,gma-MIR169f,gma-MIR169g,gma-MIR169h,gma-MIR169i,gma-MIR169j,gma-MIR169k,gma-MIR169l,gma-MIR169m,gma-MIR169n,gma-MIR169o,gma-MIR169p,gma-MIR169r,gma-MIR169s,gma-MIR169t,gma-MIR169u,gma-MIR169v,hvu-MIR169,lus-MIR169a,lus-MIR169b,lus-MIR169c,lus-MIR169d,lus-MIR169e,lus-MIR169f,lus-MIR169g,lus-MIR169h,lus-MIR169i,lus-MIR169j,lus-MIR169k,lus-MIR169l,mdm-MIR169a,mdm-MIR169b,mdm-MIR169c,mdm-MIR169d,mdm-MIR169e,mdm-MIR169f,mes-MIR169a,mes-MIR169aa,mes-MIR169ab,mes-MIR169ac,mes-MIR169b,mes-MIR169c,mes-MIR169d,mes-MIR169e,mes-MIR169f,mes-MIR169g,mes-MIR169h,mes-MIR169i,mes-MIR169j,mes-MIR169k,mes-MIR169l,mes-MIR169m,mes-MIR169n,mes-MIR169o,mes-MIR169p,mes-MIR169q,mes-MIR169r,mes-MIR169s,mes-MIR169t,mes-MIR169u,mes-MIR169v,mes-MIR169w,mes-MIR169x,mes-MIR169y,mes-MIR169z,mtr-MIR169a,mtr-MIR169b,mtr-MIR169c,mtr-MIR169d,mtr-MIR169e,mtr-MIR169f,mtr-MIR169g,mtr-MIR169h,mtr-MIR169i,mtr-MIR169j,mtr-MIR169k,mtr-MIR169l,nta-MIR169a,nta-MIR169b,nta-MIR169c,nta-MIR169d,nta-MIR169e,nta-MIR169f,nta-MIR169g,nta-MIR169h,nta-MIR169i,nta-MIR169j,nta-MIR169k,nta-MIR169l,nta-MIR169m,nta-MIR169o,nta-MIR169p,nta-MIR169q,nta-MIR169r,nta-MIR169s,nta-MIR169t,osa-MIR169a,osa-MIR169b,osa-MIR169c,osa-MIR169d,osa-MIR169e,osa-MIR169f,osa-MIR169g,osa-MIR169h,osa-MIR169i,osa-MIR169j,osa-MIR169k,osa-MIR169l,osa-MIR169m,osa-MIR169n,osa-MIR169o,osa-MIR169p,osa-MIR169q,osa-MIR169r,pde-MIR169,ppe-MIR169a,ppe-MIR169b,ppe-MIR169c,ppe-MIR169d,ppe-MIR169e,ppe-MIR169f,ppe-MIR169g,ppe-MIR169h,ppe-MIR169i,ppe-MIR169j,ppe-MIR169k,ppe-MIR169l,ptc-MIR169a,ptc-MIR169aa,ptc-MIR169ab,ptc-MIR169ac,ptc-MIR169ad,ptc-MIR169ae,ptc-MIR169af,ptc-MIR169ag,ptc-MIR169b,ptc-MIR169c,ptc-MIR169d,ptc-MIR169e,ptc-MIR169f,ptc-MIR169g,ptc-MIR169h,ptc-MIR169i,ptc-MIR169j,ptc-MIR169k,ptc-MIR169l,ptc-MIR169m,ptc-MIR169n,ptc-MIR169o,ptc-MIR169p,ptc-MIR169q,ptc-MIR169r,ptc-MIR169s,ptc-MIR169t,ptc-MIR169u,ptc-MIR169v,ptc-MIR169w,ptc-MIR169x,ptc-MIR169y,ptc-MIR169z,rco-MIR169a,rco-MIR169b,rco-MIR169c,sbi-MIR169a,sbi-MIR169b,sbi-MIR169c,sbi-MIR169d,sbi-MIR169e,sbi-MIR169f,sbi-MIR169g,sbi-MIR169h,sbi-MIR169i,sbi-MIR169j,sbi-MIR169k,sbi-MIR169l,sbi-MIR169m,sbi-MIR169n,sbi-MIR169o,sbi-MIR169p,sbi-MIR169q,sly-MIR169a,sly-MIR169b,sly-MIR169c,sly-MIR169d,sly-MIR169e,ssl-MIR169,ssp-MIR169,stu-MIR169a,stu-MIR169b,stu-MIR169c,stu-MIR169d,stu-MIR169e,stu-MIR169f,stu-MIR169g,stu-MIR169h,tae-MIR169,tcc-MIR169a,tcc-MIR169b,tcc-MIR169c,tcc-MIR169d,tcc-MIR169e,tcc-MIR169f,tcc-MIR169g,tcc-MIR169h,tcc-MIR169i,tcc-MIR169j,tcc-MIR169k,tcc-MIR169l,tcc-MIR169m,tcc-MIR169n,vun-MIR169,vvi-MIR169a,vvi-MIR169b,vvi-MIR169c,vvi-MIR169d,vvi-MIR169e,vvi-MIR169f,vvi-MIR169g,vvi-MIR169h,vvi-MIR169i,vvi-MIR169j,vvi-MIR169k,vvi-MIR169l,vvi-MIR169m,vvi-MIR169n,vvi-MIR169o,vvi-MIR169p,vvi-MIR169q,vvi-MIR169r,vvi-MIR169s,vvi-MIR169t,vvi-MIR169u,vvi-MIR169v,vvi-MIR169w,vvi-MIR169x,vvi-MIR169y,zma-MIR169a,zma-MIR169b,zma-MIR169c,zma-MIR169d,zma-MIR169e,zma-MIR169f,zma-MIR169g,zma-MIR169h,zma-MIR169i,zma-MIR169j,zma-MIR169k,zma-MIR169l,zma-MIR169m,zma-MIR169n,zma-MIR169o,zma-MIR169p,zma-MIR169q,zma-MIR169r |
| **MIR169-y** | 1 | aly-MIR169a,aly-MIR169b,aly-MIR169c,aly-MIR169d,aly-MIR169e,aly-MIR169f,aly-MIR169g,aly-MIR169h,aly-MIR169i,aly-MIR169j,aly-MIR169k,aly-MIR169l,aly-MIR169m,aly-MIR169n,aqc-MIR169a,aqc-MIR169b,aqc-MIR169c,ata-MIR169a,ata-MIR169b,ata-MIR169c,ata-MIR169d,ata-MIR169e,ata-MIR169f,ata-MIR169g,ata-MIR169h,ata-MIR169i,ata-MIR169j,ath-MIR169a,ath-MIR169b,ath-MIR169c,ath-MIR169d,ath-MIR169e,ath-MIR169f,ath-MIR169g,ath-MIR169h,ath-MIR169i,ath-MIR169j,ath-MIR169k,ath-MIR169l,ath-MIR169m,ath-MIR169n,atr-MIR169a,atr-MIR169b,atr-MIR169c,bdi-MIR169a,bdi-MIR169b,bdi-MIR169c,bdi-MIR169d,bdi-MIR169e,bdi-MIR169f,bdi-MIR169g,bdi-MIR169h,bdi-MIR169i,bdi-MIR169j,bdi-MIR169k,bdi-MIR169l,bdi-MIR169m,bdi-MIR169n,bna-MIR169a,bna-MIR169b,bna-MIR169c,bna-MIR169d,bna-MIR169e,bna-MIR169f,bna-MIR169g,bna-MIR169h,bna-MIR169i,bna-MIR169j,bna-MIR169k,bna-MIR169l,bna-MIR169m,bna-MIR169n,cca-MIR169a,cca-MIR169b,cme-MIR169a,cme-MIR169b,cme-MIR169c,cme-MIR169d,cme-MIR169e,cme-MIR169f,cme-MIR169g,cme-MIR169h,cme-MIR169i,cme-MIR169j,cme-MIR169k,cme-MIR169l,cme-MIR169m,cme-MIR169n,cme-MIR169o,cme-MIR169p,cme-MIR169q,cme-MIR169r,cme-MIR169s,cme-MIR169t,cpa-MIR169,csi-MIR169,far-MIR169,ghb-MIR169a,ghr-MIR169a,ghr-MIR169b,gma-MIR169a,gma-MIR169b,gma-MIR169c,gma-MIR169d,gma-MIR169e,gma-MIR169f,gma-MIR169g,gma-MIR169h,gma-MIR169i,gma-MIR169j,gma-MIR169k,gma-MIR169l,gma-MIR169m,gma-MIR169n,gma-MIR169o,gma-MIR169p,gma-MIR169r,gma-MIR169s,gma-MIR169t,gma-MIR169u,gma-MIR169v,hvu-MIR169,lus-MIR169a,lus-MIR169b,lus-MIR169c,lus-MIR169d,lus-MIR169e,lus-MIR169f,lus-MIR169g,lus-MIR169h,lus-MIR169i,lus-MIR169j,lus-MIR169k,lus-MIR169l,mdm-MIR169a,mdm-MIR169b,mdm-MIR169c,mdm-MIR169d,mdm-MIR169e,mdm-MIR169f,mes-MIR169a,mes-MIR169aa,mes-MIR169ab,mes-MIR169ac,mes-MIR169b,mes-MIR169c,mes-MIR169d,mes-MIR169e,mes-MIR169f,mes-MIR169g,mes-MIR169h,mes-MIR169i,mes-MIR169j,mes-MIR169k,mes-MIR169l,mes-MIR169m,mes-MIR169n,mes-MIR169o,mes-MIR169p,mes-MIR169q,mes-MIR169r,mes-MIR169s,mes-MIR169t,mes-MIR169u,mes-MIR169v,mes-MIR169w,mes-MIR169x,mes-MIR169y,mes-MIR169z,mtr-MIR169a,mtr-MIR169b,mtr-MIR169c,mtr-MIR169d,mtr-MIR169e,mtr-MIR169f,mtr-MIR169g,mtr-MIR169h,mtr-MIR169i,mtr-MIR169j,mtr-MIR169k,mtr-MIR169l,nta-MIR169a,nta-MIR169b,nta-MIR169c,nta-MIR169d,nta-MIR169e,nta-MIR169f,nta-MIR169g,nta-MIR169h,nta-MIR169i,nta-MIR169j,nta-MIR169k,nta-MIR169l,nta-MIR169m,nta-MIR169o,nta-MIR169p,nta-MIR169q,nta-MIR169r,nta-MIR169s,nta-MIR169t,osa-MIR169a,osa-MIR169b,osa-MIR169c,osa-MIR169d,osa-MIR169e,osa-MIR169f,osa-MIR169g,osa-MIR169h,osa-MIR169i,osa-MIR169j,osa-MIR169k,osa-MIR169l,osa-MIR169m,osa-MIR169n,osa-MIR169o,osa-MIR169p,osa-MIR169q,osa-MIR169r,pde-MIR169,ppe-MIR169a,ppe-MIR169b,ppe-MIR169c,ppe-MIR169d,ppe-MIR169e,ppe-MIR169f,ppe-MIR169g,ppe-MIR169h,ppe-MIR169i,ppe-MIR169j,ppe-MIR169k,ppe-MIR169l,ptc-MIR169a,ptc-MIR169aa,ptc-MIR169ab,ptc-MIR169ac,ptc-MIR169ad,ptc-MIR169ae,ptc-MIR169af,ptc-MIR169ag,ptc-MIR169b,ptc-MIR169c,ptc-MIR169d,ptc-MIR169e,ptc-MIR169f,ptc-MIR169g,ptc-MIR169h,ptc-MIR169i,ptc-MIR169j,ptc-MIR169k,ptc-MIR169l,ptc-MIR169m,ptc-MIR169n,ptc-MIR169o,ptc-MIR169p,ptc-MIR169q,ptc-MIR169r,ptc-MIR169s,ptc-MIR169t,ptc-MIR169u,ptc-MIR169v,ptc-MIR169w,ptc-MIR169x,ptc-MIR169y,ptc-MIR169z,rco-MIR169a,rco-MIR169b,rco-MIR169c,sbi-MIR169a,sbi-MIR169b,sbi-MIR169c,sbi-MIR169d,sbi-MIR169e,sbi-MIR169f,sbi-MIR169g,sbi-MIR169h,sbi-MIR169i,sbi-MIR169j,sbi-MIR169k,sbi-MIR169l,sbi-MIR169m,sbi-MIR169n,sbi-MIR169o,sbi-MIR169p,sbi-MIR169q,sly-MIR169a,sly-MIR169b,sly-MIR169c,sly-MIR169d,sly-MIR169e,ssl-MIR169,ssp-MIR169,stu-MIR169a,stu-MIR169b,stu-MIR169c,stu-MIR169d,stu-MIR169e,stu-MIR169f,stu-MIR169g,stu-MIR169h,tae-MIR169,tcc-MIR169a,tcc-MIR169b,tcc-MIR169c,tcc-MIR169d,tcc-MIR169e,tcc-MIR169f,tcc-MIR169g,tcc-MIR169h,tcc-MIR169i,tcc-MIR169j,tcc-MIR169k,tcc-MIR169l,tcc-MIR169m,tcc-MIR169n,vun-MIR169,vvi-MIR169a,vvi-MIR169b,vvi-MIR169c,vvi-MIR169d,vvi-MIR169e,vvi-MIR169f,vvi-MIR169g,vvi-MIR169h,vvi-MIR169i,vvi-MIR169j,vvi-MIR169k,vvi-MIR169l,vvi-MIR169m,vvi-MIR169n,vvi-MIR169o,vvi-MIR169p,vvi-MIR169q,vvi-MIR169r,vvi-MIR169s,vvi-MIR169t,vvi-MIR169u,vvi-MIR169v,vvi-MIR169w,vvi-MIR169x,vvi-MIR169y,zma-MIR169a,zma-MIR169b,zma-MIR169c,zma-MIR169d,zma-MIR169e,zma-MIR169f,zma-MIR169g,zma-MIR169h,zma-MIR169i,zma-MIR169j,zma-MIR169k,zma-MIR169l,zma-MIR169m,zma-MIR169n,zma-MIR169o,zma-MIR169p,zma-MIR169q,zma-MIR169r |
| **MIR170-x** | 1 | aly-MIR170,ath-MIR170 |
| **MIR171-x** | 1 | aly-MIR171a,aly-MIR171b,aly-MIR171c,aqc-MIR171a,aqc-MIR171b,aqc-MIR171c,aqc-MIR171d,aqc-MIR171e,aqc-MIR171f,ata-MIR171a,ata-MIR171b,ata-MIR171c,ata-MIR171d,ath-MIR171a,ath-MIR171b,ath-MIR171c,atr-MIR171a,atr-MIR171b,atr-MIR171c,bdi-MIR171a,bdi-MIR171b,bdi-MIR171c,bdi-MIR171d,bdi-MIR171e,bdi-MIR171f,bna-MIR171a,bna-MIR171b,bna-MIR171c,bna-MIR171d,bna-MIR171e,bna-MIR171f,bna-MIR171g,bol-MIR171a,bra-MIR171a,bra-MIR171b,bra-MIR171c,bra-MIR171d,bra-MIR171e,cca-MIR171,ccl-MIR171,cme-MIR171a,cme-MIR171b,cme-MIR171c,cme-MIR171d,cme-MIR171e,cme-MIR171f,cme-MIR171g,cme-MIR171h,cme-MIR171i,cpa-MIR171a,cpa-MIR171b,cpa-MIR171c,cpa-MIR171d,crt-MIR171,csi-MIR171a,csi-MIR171b,ctr-MIR171,far-MIR171,gma-MIR171a,gma-MIR171b,gma-MIR171c,gma-MIR171d,gma-MIR171e,gma-MIR171f,gma-MIR171g,gma-MIR171h,gma-MIR171i,gma-MIR171j,gma-MIR171k,gma-MIR171l,gma-MIR171m,gma-MIR171n,gma-MIR171o,gma-MIR171p,gma-MIR171q,gma-MIR171r,gma-MIR171s,gma-MIR171t,gma-MIR171u,hpa-MIR171a,htu-MIR171a,htu-MIR171b,htu-MIR171c,hvu-MIR171,lja-MIR171a,lja-MIR171b,lja-MIR171c,lja-MIR171d,lus-MIR171a,lus-MIR171b,lus-MIR171c,lus-MIR171d,lus-MIR171e,lus-MIR171f,lus-MIR171g,lus-MIR171h,lus-MIR171i,lus-MIR171j,mdm-MIR171a,mdm-MIR171b,mdm-MIR171c,mdm-MIR171d,mdm-MIR171e,mdm-MIR171f,mdm-MIR171g,mdm-MIR171h,mdm-MIR171i,mdm-MIR171j,mdm-MIR171k,mdm-MIR171l,mdm-MIR171m,mdm-MIR171n,mdm-MIR171o,mes-MIR171a,mes-MIR171b,mes-MIR171c,mes-MIR171d,mes-MIR171e,mes-MIR171f,mes-MIR171g,mes-MIR171h,mes-MIR171i,mes-MIR171j,mes-MIR171k,mtr-MIR171a,mtr-MIR171b,mtr-MIR171c,mtr-MIR171d,mtr-MIR171e,mtr-MIR171f,mtr-MIR171g,mtr-MIR171h,nta-MIR171a,nta-MIR171b,nta-MIR171c,osa-MIR171a,osa-MIR171b,osa-MIR171c,osa-MIR171d,osa-MIR171e,osa-MIR171f,osa-MIR171g,osa-MIR171h,osa-MIR171i,pde-MIR171,ppe-MIR171a,ppe-MIR171b,ppe-MIR171c,ppe-MIR171d,ppe-MIR171e,ppe-MIR171f,ppe-MIR171g,ppe-MIR171h,ppt-MIR171a,ppt-MIR171b,pta-MIR171,ptc-MIR171a,ptc-MIR171b,ptc-MIR171c,ptc-MIR171d,ptc-MIR171e,ptc-MIR171f,ptc-MIR171g,ptc-MIR171h,ptc-MIR171i,ptc-MIR171j,ptc-MIR171k,ptc-MIR171l,ptc-MIR171m,rco-MIR171a,rco-MIR171b,rco-MIR171c,rco-MIR171d,rco-MIR171e,rco-MIR171f,rco-MIR171g,sbi-MIR171a,sbi-MIR171b,sbi-MIR171c,sbi-MIR171d,sbi-MIR171e,sbi-MIR171f,sbi-MIR171g,sbi-MIR171h,sbi-MIR171i,sbi-MIR171j,sbi-MIR171k,sly-MIR171a,sly-MIR171b,sly-MIR171c,sly-MIR171d,sly-MIR171e,smo-MIR171a,smo-MIR171b,smo-MIR171c,smo-MIR171d,ssl-MIR171a,ssl-MIR171b,stu-MIR171a,stu-MIR171b,stu-MIR171c,stu-MIR171d,stu-MIR171e,tae-MIR171a,tae-MIR171b,tcc-MIR171a,tcc-MIR171b,tcc-MIR171c,tcc-MIR171d,tcc-MIR171e,tcc-MIR171f,tcc-MIR171g,tcc-MIR171h,vvi-MIR171a,vvi-MIR171b,vvi-MIR171c,vvi-MIR171d,vvi-MIR171e,vvi-MIR171f,vvi-MIR171g,vvi-MIR171h,vvi-MIR171i,vvi-MIR171j,zma-MIR171a,zma-MIR171b,zma-MIR171c,zma-MIR171d,zma-MIR171e,zma-MIR171f,zma-MIR171g,zma-MIR171h,zma-MIR171i,zma-MIR171j,zma-MIR171k,zma-MIR171l,zma-MIR171m,zma-MIR171n |
| **MIR171-y** | 1 | aly-MIR171a,aly-MIR171b,aly-MIR171c,aqc-MIR171a,aqc-MIR171b,aqc-MIR171c,aqc-MIR171d,aqc-MIR171e,aqc-MIR171f,ata-MIR171a,ata-MIR171b,ata-MIR171c,ata-MIR171d,ath-MIR171a,ath-MIR171b,ath-MIR171c,atr-MIR171a,atr-MIR171b,atr-MIR171c,bdi-MIR171a,bdi-MIR171b,bdi-MIR171c,bdi-MIR171d,bdi-MIR171e,bdi-MIR171f,bna-MIR171a,bna-MIR171b,bna-MIR171c,bna-MIR171d,bna-MIR171e,bna-MIR171f,bna-MIR171g,bol-MIR171a,bra-MIR171a,bra-MIR171b,bra-MIR171c,bra-MIR171d,bra-MIR171e,cca-MIR171,ccl-MIR171,cme-MIR171a,cme-MIR171b,cme-MIR171c,cme-MIR171d,cme-MIR171e,cme-MIR171f,cme-MIR171g,cme-MIR171h,cme-MIR171i,cpa-MIR171a,cpa-MIR171b,cpa-MIR171c,cpa-MIR171d,crt-MIR171,csi-MIR171a,csi-MIR171b,ctr-MIR171,far-MIR171,gma-MIR171a,gma-MIR171b,gma-MIR171c,gma-MIR171d,gma-MIR171e,gma-MIR171f,gma-MIR171g,gma-MIR171h,gma-MIR171i,gma-MIR171j,gma-MIR171k,gma-MIR171l,gma-MIR171m,gma-MIR171n,gma-MIR171o,gma-MIR171p,gma-MIR171q,gma-MIR171r,gma-MIR171s,gma-MIR171t,gma-MIR171u,hpa-MIR171a,htu-MIR171a,htu-MIR171b,htu-MIR171c,hvu-MIR171,lja-MIR171a,lja-MIR171b,lja-MIR171c,lja-MIR171d,lus-MIR171a,lus-MIR171b,lus-MIR171c,lus-MIR171d,lus-MIR171e,lus-MIR171f,lus-MIR171g,lus-MIR171h,lus-MIR171i,lus-MIR171j,mdm-MIR171a,mdm-MIR171b,mdm-MIR171c,mdm-MIR171d,mdm-MIR171e,mdm-MIR171f,mdm-MIR171g,mdm-MIR171h,mdm-MIR171i,mdm-MIR171j,mdm-MIR171k,mdm-MIR171l,mdm-MIR171m,mdm-MIR171n,mdm-MIR171o,mes-MIR171a,mes-MIR171b,mes-MIR171c,mes-MIR171d,mes-MIR171e,mes-MIR171f,mes-MIR171g,mes-MIR171h,mes-MIR171i,mes-MIR171j,mes-MIR171k,mtr-MIR171a,mtr-MIR171b,mtr-MIR171c,mtr-MIR171d,mtr-MIR171e,mtr-MIR171f,mtr-MIR171g,mtr-MIR171h,nta-MIR171a,nta-MIR171b,nta-MIR171c,osa-MIR171a,osa-MIR171b,osa-MIR171c,osa-MIR171d,osa-MIR171e,osa-MIR171f,osa-MIR171g,osa-MIR171h,osa-MIR171i,pde-MIR171,ppe-MIR171a,ppe-MIR171b,ppe-MIR171c,ppe-MIR171d,ppe-MIR171e,ppe-MIR171f,ppe-MIR171g,ppe-MIR171h,ppt-MIR171a,ppt-MIR171b,pta-MIR171,ptc-MIR171a,ptc-MIR171b,ptc-MIR171c,ptc-MIR171d,ptc-MIR171e,ptc-MIR171f,ptc-MIR171g,ptc-MIR171h,ptc-MIR171i,ptc-MIR171j,ptc-MIR171k,ptc-MIR171l,ptc-MIR171m,rco-MIR171a,rco-MIR171b,rco-MIR171c,rco-MIR171d,rco-MIR171e,rco-MIR171f,rco-MIR171g,sbi-MIR171a,sbi-MIR171b,sbi-MIR171c,sbi-MIR171d,sbi-MIR171e,sbi-MIR171f,sbi-MIR171g,sbi-MIR171h,sbi-MIR171i,sbi-MIR171j,sbi-MIR171k,sly-MIR171a,sly-MIR171b,sly-MIR171c,sly-MIR171d,sly-MIR171e,smo-MIR171a,smo-MIR171b,smo-MIR171c,smo-MIR171d,ssl-MIR171a,ssl-MIR171b,stu-MIR171a,stu-MIR171b,stu-MIR171c,stu-MIR171d,stu-MIR171e,tae-MIR171a,tae-MIR171b,tcc-MIR171a,tcc-MIR171b,tcc-MIR171c,tcc-MIR171d,tcc-MIR171e,tcc-MIR171f,tcc-MIR171g,tcc-MIR171h,vvi-MIR171a,vvi-MIR171b,vvi-MIR171c,vvi-MIR171d,vvi-MIR171e,vvi-MIR171f,vvi-MIR171g,vvi-MIR171h,vvi-MIR171i,vvi-MIR171j,zma-MIR171a,zma-MIR171b,zma-MIR171c,zma-MIR171d,zma-MIR171e,zma-MIR171f,zma-MIR171g,zma-MIR171h,zma-MIR171i,zma-MIR171j,zma-MIR171k,zma-MIR171l,zma-MIR171m,zma-MIR171n |
| **MIR171-z** | 1 | aly-MIR171a,aly-MIR171b,aly-MIR171c,aqc-MIR171a,aqc-MIR171b,aqc-MIR171c,aqc-MIR171d,aqc-MIR171e,aqc-MIR171f,ata-MIR171a,ata-MIR171b,ata-MIR171c,ata-MIR171d,ath-MIR171a,ath-MIR171b,ath-MIR171c,atr-MIR171a,atr-MIR171b,atr-MIR171c,bdi-MIR171a,bdi-MIR171b,bdi-MIR171c,bdi-MIR171d,bdi-MIR171e,bdi-MIR171f,bna-MIR171a,bna-MIR171b,bna-MIR171c,bna-MIR171d,bna-MIR171e,bna-MIR171f,bna-MIR171g,bol-MIR171a,bra-MIR171a,bra-MIR171b,bra-MIR171c,bra-MIR171d,bra-MIR171e,cca-MIR171,ccl-MIR171,cme-MIR171a,cme-MIR171b,cme-MIR171c,cme-MIR171d,cme-MIR171e,cme-MIR171f,cme-MIR171g,cme-MIR171h,cme-MIR171i,cpa-MIR171a,cpa-MIR171b,cpa-MIR171c,cpa-MIR171d,crt-MIR171,csi-MIR171a,csi-MIR171b,ctr-MIR171,far-MIR171,gma-MIR171a,gma-MIR171b,gma-MIR171c,gma-MIR171d,gma-MIR171e,gma-MIR171f,gma-MIR171g,gma-MIR171h,gma-MIR171i,gma-MIR171j,gma-MIR171k,gma-MIR171l,gma-MIR171m,gma-MIR171n,gma-MIR171o,gma-MIR171p,gma-MIR171q,gma-MIR171r,gma-MIR171s,gma-MIR171t,gma-MIR171u,hpa-MIR171a,htu-MIR171a,htu-MIR171b,htu-MIR171c,hvu-MIR171,lja-MIR171a,lja-MIR171b,lja-MIR171c,lja-MIR171d,lus-MIR171a,lus-MIR171b,lus-MIR171c,lus-MIR171d,lus-MIR171e,lus-MIR171f,lus-MIR171g,lus-MIR171h,lus-MIR171i,lus-MIR171j,mdm-MIR171a,mdm-MIR171b,mdm-MIR171c,mdm-MIR171d,mdm-MIR171e,mdm-MIR171f,mdm-MIR171g,mdm-MIR171h,mdm-MIR171i,mdm-MIR171j,mdm-MIR171k,mdm-MIR171l,mdm-MIR171m,mdm-MIR171n,mdm-MIR171o,mes-MIR171a,mes-MIR171b,mes-MIR171c,mes-MIR171d,mes-MIR171e,mes-MIR171f,mes-MIR171g,mes-MIR171h,mes-MIR171i,mes-MIR171j,mes-MIR171k,mtr-MIR171a,mtr-MIR171b,mtr-MIR171c,mtr-MIR171d,mtr-MIR171e,mtr-MIR171f,mtr-MIR171g,mtr-MIR171h,nta-MIR171a,nta-MIR171b,nta-MIR171c,osa-MIR171a,osa-MIR171b,osa-MIR171c,osa-MIR171d,osa-MIR171e,osa-MIR171f,osa-MIR171g,osa-MIR171h,osa-MIR171i,pde-MIR171,ppe-MIR171a,ppe-MIR171b,ppe-MIR171c,ppe-MIR171d,ppe-MIR171e,ppe-MIR171f,ppe-MIR171g,ppe-MIR171h,ppt-MIR171a,ppt-MIR171b,pta-MIR171,ptc-MIR171a,ptc-MIR171b,ptc-MIR171c,ptc-MIR171d,ptc-MIR171e,ptc-MIR171f,ptc-MIR171g,ptc-MIR171h,ptc-MIR171i,ptc-MIR171j,ptc-MIR171k,ptc-MIR171l,ptc-MIR171m,rco-MIR171a,rco-MIR171b,rco-MIR171c,rco-MIR171d,rco-MIR171e,rco-MIR171f,rco-MIR171g,sbi-MIR171a,sbi-MIR171b,sbi-MIR171c,sbi-MIR171d,sbi-MIR171e,sbi-MIR171f,sbi-MIR171g,sbi-MIR171h,sbi-MIR171i,sbi-MIR171j,sbi-MIR171k,sly-MIR171a,sly-MIR171b,sly-MIR171c,sly-MIR171d,sly-MIR171e,smo-MIR171a,smo-MIR171b,smo-MIR171c,smo-MIR171d,ssl-MIR171a,ssl-MIR171b,stu-MIR171a,stu-MIR171b,stu-MIR171c,stu-MIR171d,stu-MIR171e,tae-MIR171a,tae-MIR171b,tcc-MIR171a,tcc-MIR171b,tcc-MIR171c,tcc-MIR171d,tcc-MIR171e,tcc-MIR171f,tcc-MIR171g,tcc-MIR171h,vvi-MIR171a,vvi-MIR171b,vvi-MIR171c,vvi-MIR171d,vvi-MIR171e,vvi-MIR171f,vvi-MIR171g,vvi-MIR171h,vvi-MIR171i,vvi-MIR171j,zma-MIR171a,zma-MIR171b,zma-MIR171c,zma-MIR171d,zma-MIR171e,zma-MIR171f,zma-MIR171g,zma-MIR171h,zma-MIR171i,zma-MIR171j,zma-MIR171k,zma-MIR171l,zma-MIR171m,zma-MIR171n |
| **MIR172-y** | 1 | aau-MIR172,aly-MIR172a,aly-MIR172b,aly-MIR172c,aly-MIR172d,aly-MIR172e,aly-MIR172f,aqc-MIR172a,aqc-MIR172b,ata-MIR172a,ata-MIR172b,ata-MIR172c,ath-MIR172a,ath-MIR172b,ath-MIR172c,ath-MIR172d,ath-MIR172e,atr-MIR172,bdi-MIR172a,bdi-MIR172b,bdi-MIR172d,bna-MIR172a,bna-MIR172b,bna-MIR172c,bna-MIR172d,bol-MIR172a,bol-MIR172b,bra-MIR172a,bra-MIR172b,bra-MIR172c,bra-MIR172d,cca-MIR172,cme-MIR172a,cme-MIR172b,cme-MIR172c,cme-MIR172d,cme-MIR172e,cme-MIR172f,cpa-MIR172a,cpa-MIR172b,csi-MIR172a,csi-MIR172b,csi-MIR172c,dpr-MIR172a,dpr-MIR172b,egu-MIR172a,egu-MIR172b,egu-MIR172c,egu-MIR172d,egu-MIR172e,egu-MIR172f,ghr-MIR172,gma-MIR172a,gma-MIR172b,gma-MIR172c,gma-MIR172d,gma-MIR172e,gma-MIR172f,gma-MIR172g,gma-MIR172h,gma-MIR172i,gma-MIR172j,gma-MIR172k,gma-MIR172l,gra-MIR172a,gra-MIR172b,lja-MIR172a,lja-MIR172b,lja-MIR172c,lus-MIR172a,lus-MIR172b,lus-MIR172c,lus-MIR172d,lus-MIR172e,lus-MIR172f,lus-MIR172g,lus-MIR172h,lus-MIR172i,lus-MIR172j,mdm-MIR172a,mdm-MIR172b,mdm-MIR172c,mdm-MIR172d,mdm-MIR172e,mdm-MIR172f,mdm-MIR172g,mdm-MIR172h,mdm-MIR172i,mdm-MIR172j,mdm-MIR172k,mdm-MIR172l,mdm-MIR172m,mdm-MIR172n,mdm-MIR172o,mes-MIR172a,mes-MIR172b,mes-MIR172c,mes-MIR172d,mes-MIR172e,mes-MIR172f,mtr-MIR172a,mtr-MIR172b,mtr-MIR172c,mtr-MIR172d,nta-MIR172a,nta-MIR172b,nta-MIR172c,nta-MIR172d,nta-MIR172e,nta-MIR172f,nta-MIR172g,nta-MIR172h,nta-MIR172i,nta-MIR172j,osa-MIR172a,osa-MIR172b,osa-MIR172c,osa-MIR172d,ppe-MIR172a,ppe-MIR172b,ppe-MIR172c,ppe-MIR172d,ptc-MIR172a,ptc-MIR172b,ptc-MIR172c,ptc-MIR172d,ptc-MIR172e,ptc-MIR172f,ptc-MIR172g,ptc-MIR172h,ptc-MIR172i,rco-MIR172,sbi-MIR172a,sbi-MIR172b,sbi-MIR172c,sbi-MIR172d,sbi-MIR172e,sbi-MIR172f,sly-MIR172a,sly-MIR172b,ssl-MIR172,stu-MIR172a,stu-MIR172b,stu-MIR172c,stu-MIR172d,stu-MIR172e,tcc-MIR172a,tcc-MIR172b,tcc-MIR172c,tcc-MIR172d,tcc-MIR172e,vun-MIR172,vvi-MIR172a,vvi-MIR172b,vvi-MIR172c,vvi-MIR172d,zma-MIR172a,zma-MIR172b,zma-MIR172c,zma-MIR172d,zma-MIR172e |
| **MIR1863-y** | 1 | cme-MIR1863,osa-MIR1863a,osa-MIR1863b,osa-MIR1863c,pab-MIR1863 |
| **MIR1871-y** | 1 | osa-MIR1871 |
| **MIR1873-x** | 1 | osa-MIR1873 |
| **MIR1882-y** | 1 | osa-MIR1882a,osa-MIR1882b,osa-MIR1882c,osa-MIR1882d,osa-MIR1882e,osa-MIR1882f,osa-MIR1882g,osa-MIR1882h |
| **MIR2092-y** | 1 | osa-MIR2092 |
| **MIR2118-y** | 1 | ata-MIR2118a,ata-MIR2118b,ata-MIR2118c,ata-MIR2118d,bdi-MIR2118a,bdi-MIR2118b,gma-MIR2118a,gma-MIR2118b,hbr-MIR2118,mdm-MIR2118a,mdm-MIR2118b,mdm-MIR2118c,mtr-MIR2118,osa-MIR2118a,osa-MIR2118b,osa-MIR2118c,osa-MIR2118d,osa-MIR2118e,osa-MIR2118f,osa-MIR2118g,osa-MIR2118h,osa-MIR2118i,osa-MIR2118j,osa-MIR2118k,osa-MIR2118l,osa-MIR2118m,osa-MIR2118n,osa-MIR2118o,osa-MIR2118p,osa-MIR2118q,osa-MIR2118r,pgi-MIR2118,pvu-MIR2118,sbi-MIR2118,vun-MIR2118,zma-MIR2118a,zma-MIR2118b,zma-MIR2118c,zma-MIR2118d,zma-MIR2118e,zma-MIR2118f,zma-MIR2118g |
| **MIR2119-y** | 1 | gma-MIR2119,mtr-MIR2119,pvu-MIR2119 |
| **MIR2275-x** | 1 | ata-MIR2275a,ata-MIR2275b,ata-MIR2275c,bdi-MIR2275a,bdi-MIR2275b,bdi-MIR2275c,mes-MIR2275,osa-MIR2275a,osa-MIR2275b,osa-MIR2275c,osa-MIR2275d,tae-MIR2275,zma-MIR2275a,zma-MIR2275b,zma-MIR2275c,zma-MIR2275d |
| **MIR2624-x** | 1 | mtr-MIR2624 |
| **MIR2655-x** | 1 | mtr-MIR2655a,mtr-MIR2655b,mtr-MIR2655c,mtr-MIR2655d,mtr-MIR2655e,mtr-MIR2655f,mtr-MIR2655g,mtr-MIR2655h,mtr-MIR2655i,mtr-MIR2655j,mtr-MIR2655k,mtr-MIR2655l,mtr-MIR2655m,mtr-MIR2655n,mtr-MIR2655o |
| **MIR2916-x** | 1 | peu-MIR2916 |
| **MIR2938-y** | 1 | ath-MIR2938 |
| **MIR319-y** | 1 | aau-MIR319,aly-MIR319a,aly-MIR319b,aly-MIR319c,aly-MIR319d,amg-MIR319,aqc-MIR319,ata-MIR319,ath-MIR319a,ath-MIR319b,ath-MIR319c,atr-MIR319a,atr-MIR319b,atr-MIR319c,atr-MIR319d,atr-MIR319e,bdi-MIR319a,bdi-MIR319b,bra-MIR319,cca-MIR319,cme-MIR319a,cme-MIR319b,cme-MIR319c,cme-MIR319d,cpa-MIR319,csi-MIR319,ctr-MIR319,gma-MIR319a,gma-MIR319b,gma-MIR319c,gma-MIR319d,gma-MIR319e,gma-MIR319f,gma-MIR319g,gma-MIR319h,gma-MIR319i,gma-MIR319j,gma-MIR319k,gma-MIR319l,gma-MIR319m,gma-MIR319n,gma-MIR319o,gma-MIR319p,gma-MIR319q,hbr-MIR319,lus-MIR319a,lus-MIR319b,mdm-MIR319a,mdm-MIR319b,mdm-MIR319c,mes-MIR319a,mes-MIR319b,mes-MIR319c,mes-MIR319d,mes-MIR319e,mes-MIR319f,mes-MIR319g,mes-MIR319h,mtr-MIR319a,mtr-MIR319b,mtr-MIR319c,mtr-MIR319d,nta-MIR319a,nta-MIR319b,osa-MIR319a,osa-MIR319b,ppe-MIR319a,ppe-MIR319b,ppt-MIR319a,ppt-MIR319b,ppt-MIR319c,ppt-MIR319d,ppt-MIR319e,pta-MIR319,ptc-MIR319a,ptc-MIR319b,ptc-MIR319c,ptc-MIR319d,ptc-MIR319e,ptc-MIR319f,ptc-MIR319g,ptc-MIR319h,ptc-MIR319i,pvu-MIR319c,rco-MIR319a,rco-MIR319b,rco-MIR319c,rco-MIR319d,sbi-MIR319a,sbi-MIR319b,sly-MIR319a,sly-MIR319b,sly-MIR319c,smo-MIR319,stu-MIR319,stu-MIR319a,stu-MIR319b,tae-MIR319,tcc-MIR319,vun-MIR319a,vun-MIR319b,vvi-MIR319b,vvi-MIR319c,vvi-MIR319e,vvi-MIR319f,vvi-MIR319g,zma-MIR319a,zma-MIR319b,zma-MIR319c,zma-MIR319d |
| **MIR3627-x** | 1 | mdm-MIR3627a,mdm-MIR3627b,mdm-MIR3627c,ppe-MIR3627,ptc-MIR3627a,ptc-MIR3627b,stu-MIR3627,vvi-MIR3627 |
| **MIR3630-x** | 1 | han-MIR3630,vvi-MIR3630 |
| **MIR3630-y** | 1 | han-MIR3630,vvi-MIR3630 |
| **MIR384-x** | 1 | stu-MIR384 |
| **MIR390-x** | 1 | aly-MIR390a,aly-MIR390b,ata-MIR390,ath-MIR390a,ath-MIR390b,atr-MIR390,bdi-MIR390a,bna-MIR390a,bna-MIR390b,bna-MIR390c,bra-MIR390,cca-MIR390,cme-MIR390a,cme-MIR390b,cme-MIR390c,cme-MIR390d,cpa-MIR390a,cpa-MIR390b,csi-MIR390,ghr-MIR390a,ghr-MIR390b,ghr-MIR390c,gma-MIR390a,gma-MIR390b,gma-MIR390c,gma-MIR390d,gma-MIR390e,gma-MIR390f,gma-MIR390g,hex-MIR390a,hex-MIR390b,lja-MIR390a,lja-MIR390b,lus-MIR390a,lus-MIR390b,lus-MIR390c,lus-MIR390d,mdm-MIR390a,mdm-MIR390b,mdm-MIR390c,mdm-MIR390d,mdm-MIR390e,mdm-MIR390f,mes-MIR390,mtr-MIR390,nta-MIR390a,nta-MIR390b,nta-MIR390c,osa-MIR390,pde-MIR390,ppe-MIR390,ppt-MIR390a,ppt-MIR390b,ppt-MIR390c,pta-MIR390,ptc-MIR390a,ptc-MIR390b,ptc-MIR390c,ptc-MIR390d,rco-MIR390a,rco-MIR390b,sbi-MIR390,sly-MIR390a,sly-MIR390b,stu-MIR390,tcc-MIR390a,tcc-MIR390b,vvi-MIR390,zma-MIR390a,zma-MIR390b |
| **MIR390-y** | 1 | aly-MIR390a,aly-MIR390b,ata-MIR390,ath-MIR390a,ath-MIR390b,atr-MIR390,bdi-MIR390a,bna-MIR390a,bna-MIR390b,bna-MIR390c,bra-MIR390,cca-MIR390,cme-MIR390a,cme-MIR390b,cme-MIR390c,cme-MIR390d,cpa-MIR390a,cpa-MIR390b,csi-MIR390,ghr-MIR390a,ghr-MIR390b,ghr-MIR390c,gma-MIR390a,gma-MIR390b,gma-MIR390c,gma-MIR390d,gma-MIR390e,gma-MIR390f,gma-MIR390g,hex-MIR390a,hex-MIR390b,lja-MIR390a,lja-MIR390b,lus-MIR390a,lus-MIR390b,lus-MIR390c,lus-MIR390d,mdm-MIR390a,mdm-MIR390b,mdm-MIR390c,mdm-MIR390d,mdm-MIR390e,mdm-MIR390f,mes-MIR390,mtr-MIR390,nta-MIR390a,nta-MIR390b,nta-MIR390c,osa-MIR390,pde-MIR390,ppe-MIR390,ppt-MIR390a,ppt-MIR390b,ppt-MIR390c,pta-MIR390,ptc-MIR390a,ptc-MIR390b,ptc-MIR390c,ptc-MIR390d,rco-MIR390a,rco-MIR390b,sbi-MIR390,sly-MIR390a,sly-MIR390b,stu-MIR390,tcc-MIR390a,tcc-MIR390b,vvi-MIR390,zma-MIR390a,zma-MIR390b |
| **MIR393-x** | 1 | aly-MIR393a,aly-MIR393b,ata-MIR393,ath-MIR393a,ath-MIR393b,atr-MIR393,bdi-MIR393a,bdi-MIR393b,bna-MIR393,cca-MIR393,cme-MIR393a,cme-MIR393b,cme-MIR393c,cpa-MIR393,csi-MIR393,ghr-MIR393,gma-MIR393a,gma-MIR393b,gma-MIR393c,gma-MIR393d,gma-MIR393e,gma-MIR393f,gma-MIR393g,gma-MIR393h,gma-MIR393i,gma-MIR393j,gma-MIR393k,htu-MIR393a,htu-MIR393b,htu-MIR393c,lus-MIR393a,lus-MIR393b,lus-MIR393c,lus-MIR393d,mdm-MIR393a,mdm-MIR393b,mdm-MIR393c,mdm-MIR393d,mdm-MIR393e,mdm-MIR393f,mes-MIR393a,mes-MIR393b,mes-MIR393c,mes-MIR393d,mtr-MIR393a,mtr-MIR393b,osa-MIR393a,osa-MIR393b,ppe-MIR393a,ppe-MIR393b,ptc-MIR393a,ptc-MIR393b,ptc-MIR393c,rco-MIR393,sbi-MIR393a,sbi-MIR393b,stu-MIR393,tcc-MIR393a,tcc-MIR393b,vvi-MIR393a,vvi-MIR393b,zma-MIR393a,zma-MIR393b,zma-MIR393c |
| **MIR393-y** | 1 | aly-MIR393a,aly-MIR393b,ata-MIR393,ath-MIR393a,ath-MIR393b,atr-MIR393,bdi-MIR393a,bdi-MIR393b,bna-MIR393,cca-MIR393,cme-MIR393a,cme-MIR393b,cme-MIR393c,cpa-MIR393,csi-MIR393,ghr-MIR393,gma-MIR393a,gma-MIR393b,gma-MIR393c,gma-MIR393d,gma-MIR393e,gma-MIR393f,gma-MIR393g,gma-MIR393h,gma-MIR393i,gma-MIR393j,gma-MIR393k,htu-MIR393a,htu-MIR393b,htu-MIR393c,lus-MIR393a,lus-MIR393b,lus-MIR393c,lus-MIR393d,mdm-MIR393a,mdm-MIR393b,mdm-MIR393c,mdm-MIR393d,mdm-MIR393e,mdm-MIR393f,mes-MIR393a,mes-MIR393b,mes-MIR393c,mes-MIR393d,mtr-MIR393a,mtr-MIR393b,osa-MIR393a,osa-MIR393b,ppe-MIR393a,ppe-MIR393b,ptc-MIR393a,ptc-MIR393b,ptc-MIR393c,rco-MIR393,sbi-MIR393a,sbi-MIR393b,stu-MIR393,tcc-MIR393a,tcc-MIR393b,vvi-MIR393a,vvi-MIR393b,zma-MIR393a,zma-MIR393b,zma-MIR393c |
| **MIR3932-x** | 1 | ath-MIR3932a,ath-MIR3932b |
| **MIR394-x** | 1 | ahy-MIR394,aly-MIR394a,aly-MIR394b,ata-MIR394,ath-MIR394a,ath-MIR394b,atr-MIR394,bdi-MIR394,bna-MIR394a,bna-MIR394b,cca-MIR394,cme-MIR394a,cme-MIR394b,cpa-MIR394a,cpa-MIR394b,csi-MIR394,ghr-MIR394a,ghr-MIR394b,gma-MIR394a,gma-MIR394b,gma-MIR394c,gma-MIR394d,gma-MIR394e,gma-MIR394f,gma-MIR394g,lus-MIR394a,lus-MIR394b,mdm-MIR394a,mdm-MIR394b,mes-MIR394a,mes-MIR394b,mes-MIR394c,nta-MIR394,osa-MIR394,ppe-MIR394a,ppe-MIR394b,ptc-MIR394a,ptc-MIR394b,sbi-MIR394a,sbi-MIR394b,sly-MIR394,ssl-MIR394,tcc-MIR394a,tcc-MIR394b,vvi-MIR394a,vvi-MIR394b,vvi-MIR394c,zma-MIR394a,zma-MIR394b |
| **MIR394-y** | 1 | ahy-MIR394,aly-MIR394a,aly-MIR394b,ata-MIR394,ath-MIR394a,ath-MIR394b,atr-MIR394,bdi-MIR394,bna-MIR394a,bna-MIR394b,cca-MIR394,cme-MIR394a,cme-MIR394b,cpa-MIR394a,cpa-MIR394b,csi-MIR394,ghr-MIR394a,ghr-MIR394b,gma-MIR394a,gma-MIR394b,gma-MIR394c,gma-MIR394d,gma-MIR394e,gma-MIR394f,gma-MIR394g,lus-MIR394a,lus-MIR394b,mdm-MIR394a,mdm-MIR394b,mes-MIR394a,mes-MIR394b,mes-MIR394c,nta-MIR394,osa-MIR394,ppe-MIR394a,ppe-MIR394b,ptc-MIR394a,ptc-MIR394b,sbi-MIR394a,sbi-MIR394b,sly-MIR394,ssl-MIR394,tcc-MIR394a,tcc-MIR394b,vvi-MIR394a,vvi-MIR394b,vvi-MIR394c,zma-MIR394a,zma-MIR394b |
| **MIR395-x** | 1 | aly-MIR395b,aly-MIR395c,aly-MIR395d,aly-MIR395e,aly-MIR395f,aly-MIR395g,aly-MIR395h,aly-MIR395i,aqc-MIR395a,aqc-MIR395b,ata-MIR395a,ata-MIR395b,ata-MIR395c,ata-MIR395d,ata-MIR395e,ata-MIR395f,ath-MIR395a,ath-MIR395b,ath-MIR395c,ath-MIR395d,ath-MIR395e,ath-MIR395f,atr-MIR395,bdi-MIR395a,bdi-MIR395b,bdi-MIR395c,bdi-MIR395d,bdi-MIR395e,bdi-MIR395f,bdi-MIR395g,bdi-MIR395h,bdi-MIR395j,bdi-MIR395k,bdi-MIR395l,bdi-MIR395m,bdi-MIR395n,bdi-MIR395o,bdi-MIR395p,bdi-MIR395q,bna-MIR395a,bna-MIR395b,bna-MIR395c,bna-MIR395d,bna-MIR395e,bna-MIR395f,bra-MIR395a,bra-MIR395b,bra-MIR395c,bra-MIR395d,cca-MIR395a,cca-MIR395b,cca-MIR395c,cme-MIR395a,cme-MIR395b,cme-MIR395c,cme-MIR395d,cme-MIR395e,cme-MIR395f,cpa-MIR395a,cpa-MIR395b,cpa-MIR395c,cpa-MIR395d,cpa-MIR395e,csi-MIR395,gma-MIR395a,gma-MIR395b,gma-MIR395c,gma-MIR395d,gma-MIR395e,gma-MIR395f,gma-MIR395g,gma-MIR395h,gma-MIR395i,gma-MIR395j,gma-MIR395k,gma-MIR395l,gma-MIR395m,lus-MIR395a,lus-MIR395b,lus-MIR395c,lus-MIR395d,lus-MIR395e,mdm-MIR395a,mdm-MIR395b,mdm-MIR395c,mdm-MIR395d,mdm-MIR395e,mdm-MIR395f,mdm-MIR395g,mdm-MIR395h,mdm-MIR395i,mes-MIR395a,mes-MIR395b,mes-MIR395c,mes-MIR395d,mes-MIR395e,mtr-MIR395a,mtr-MIR395b,mtr-MIR395c,mtr-MIR395d,mtr-MIR395e,mtr-MIR395f,mtr-MIR395g,mtr-MIR395h,mtr-MIR395i,mtr-MIR395j,mtr-MIR395k,mtr-MIR395l,mtr-MIR395m,mtr-MIR395n,mtr-MIR395o,nta-MIR395a,nta-MIR395b,nta-MIR395c,osa-MIR395a,osa-MIR395b,osa-MIR395c,osa-MIR395d,osa-MIR395e,osa-MIR395f,osa-MIR395g,osa-MIR395h,osa-MIR395i,osa-MIR395j,osa-MIR395k,osa-MIR395l,osa-MIR395m,osa-MIR395n,osa-MIR395o,osa-MIR395p,osa-MIR395q,osa-MIR395r,osa-MIR395s,osa-MIR395t,osa-MIR395u,osa-MIR395v,osa-MIR395w,osa-MIR395x,osa-MIR395y,pab-MIR395,ppe-MIR395a,ppe-MIR395b,ppe-MIR395c,ppe-MIR395d,ppe-MIR395e,ppe-MIR395f,ppe-MIR395g,ppe-MIR395h,ppe-MIR395i,ppe-MIR395j,ppe-MIR395k,ppe-MIR395l,ppe-MIR395m,ppe-MIR395n,ppe-MIR395o,ppt-MIR395,ptc-MIR395a,ptc-MIR395b,ptc-MIR395c,ptc-MIR395d,ptc-MIR395e,ptc-MIR395f,ptc-MIR395g,ptc-MIR395h,ptc-MIR395i,ptc-MIR395j,ptc-MIR395k,rco-MIR395a,rco-MIR395b,rco-MIR395c,rco-MIR395d,rco-MIR395e,sbi-MIR395a,sbi-MIR395b,sbi-MIR395c,sbi-MIR395d,sbi-MIR395e,sbi-MIR395f,sbi-MIR395g,sbi-MIR395h,sbi-MIR395i,sbi-MIR395j,sbi-MIR395k,sbi-MIR395l,sly-MIR395a,sly-MIR395b,ssl-MIR395,stu-MIR395a,stu-MIR395b,stu-MIR395c,stu-MIR395d,stu-MIR395e,stu-MIR395f,stu-MIR395g,stu-MIR395h,stu-MIR395i,stu-MIR395j,tae-MIR395a,tae-MIR395b,tcc-MIR395a,tcc-MIR395b,vun-MIR395,vvi-MIR395a,vvi-MIR395b,vvi-MIR395c,vvi-MIR395d,vvi-MIR395e,vvi-MIR395f,vvi-MIR395g,vvi-MIR395h,vvi-MIR395i,vvi-MIR395j,vvi-MIR395k,vvi-MIR395l,vvi-MIR395m,vvi-MIR395n,zma-MIR395a,zma-MIR395b,zma-MIR395c,zma-MIR395d,zma-MIR395e,zma-MIR395f,zma-MIR395g,zma-MIR395h,zma-MIR395i,zma-MIR395j,zma-MIR395k,zma-MIR395l,zma-MIR395m,zma-MIR395n,zma-MIR395o,zma-MIR395p |
| **MIR395-y** | 1 | aly-MIR395b,aly-MIR395c,aly-MIR395d,aly-MIR395e,aly-MIR395f,aly-MIR395g,aly-MIR395h,aly-MIR395i,aqc-MIR395a,aqc-MIR395b,ata-MIR395a,ata-MIR395b,ata-MIR395c,ata-MIR395d,ata-MIR395e,ata-MIR395f,ath-MIR395a,ath-MIR395b,ath-MIR395c,ath-MIR395d,ath-MIR395e,ath-MIR395f,atr-MIR395,bdi-MIR395a,bdi-MIR395b,bdi-MIR395c,bdi-MIR395d,bdi-MIR395e,bdi-MIR395f,bdi-MIR395g,bdi-MIR395h,bdi-MIR395j,bdi-MIR395k,bdi-MIR395l,bdi-MIR395m,bdi-MIR395n,bdi-MIR395o,bdi-MIR395p,bdi-MIR395q,bna-MIR395a,bna-MIR395b,bna-MIR395c,bna-MIR395d,bna-MIR395e,bna-MIR395f,bra-MIR395a,bra-MIR395b,bra-MIR395c,bra-MIR395d,cca-MIR395a,cca-MIR395b,cca-MIR395c,cme-MIR395a,cme-MIR395b,cme-MIR395c,cme-MIR395d,cme-MIR395e,cme-MIR395f,cpa-MIR395a,cpa-MIR395b,cpa-MIR395c,cpa-MIR395d,cpa-MIR395e,csi-MIR395,gma-MIR395a,gma-MIR395b,gma-MIR395c,gma-MIR395d,gma-MIR395e,gma-MIR395f,gma-MIR395g,gma-MIR395h,gma-MIR395i,gma-MIR395j,gma-MIR395k,gma-MIR395l,gma-MIR395m,lus-MIR395a,lus-MIR395b,lus-MIR395c,lus-MIR395d,lus-MIR395e,mdm-MIR395a,mdm-MIR395b,mdm-MIR395c,mdm-MIR395d,mdm-MIR395e,mdm-MIR395f,mdm-MIR395g,mdm-MIR395h,mdm-MIR395i,mes-MIR395a,mes-MIR395b,mes-MIR395c,mes-MIR395d,mes-MIR395e,mtr-MIR395a,mtr-MIR395b,mtr-MIR395c,mtr-MIR395d,mtr-MIR395e,mtr-MIR395f,mtr-MIR395g,mtr-MIR395h,mtr-MIR395i,mtr-MIR395j,mtr-MIR395k,mtr-MIR395l,mtr-MIR395m,mtr-MIR395n,mtr-MIR395o,nta-MIR395a,nta-MIR395b,nta-MIR395c,osa-MIR395a,osa-MIR395b,osa-MIR395c,osa-MIR395d,osa-MIR395e,osa-MIR395f,osa-MIR395g,osa-MIR395h,osa-MIR395i,osa-MIR395j,osa-MIR395k,osa-MIR395l,osa-MIR395m,osa-MIR395n,osa-MIR395o,osa-MIR395p,osa-MIR395q,osa-MIR395r,osa-MIR395s,osa-MIR395t,osa-MIR395u,osa-MIR395v,osa-MIR395w,osa-MIR395x,osa-MIR395y,pab-MIR395,ppe-MIR395a,ppe-MIR395b,ppe-MIR395c,ppe-MIR395d,ppe-MIR395e,ppe-MIR395f,ppe-MIR395g,ppe-MIR395h,ppe-MIR395i,ppe-MIR395j,ppe-MIR395k,ppe-MIR395l,ppe-MIR395m,ppe-MIR395n,ppe-MIR395o,ppt-MIR395,ptc-MIR395a,ptc-MIR395b,ptc-MIR395c,ptc-MIR395d,ptc-MIR395e,ptc-MIR395f,ptc-MIR395g,ptc-MIR395h,ptc-MIR395i,ptc-MIR395j,ptc-MIR395k,rco-MIR395a,rco-MIR395b,rco-MIR395c,rco-MIR395d,rco-MIR395e,sbi-MIR395a,sbi-MIR395b,sbi-MIR395c,sbi-MIR395d,sbi-MIR395e,sbi-MIR395f,sbi-MIR395g,sbi-MIR395h,sbi-MIR395i,sbi-MIR395j,sbi-MIR395k,sbi-MIR395l,sly-MIR395a,sly-MIR395b,ssl-MIR395,stu-MIR395a,stu-MIR395b,stu-MIR395c,stu-MIR395d,stu-MIR395e,stu-MIR395f,stu-MIR395g,stu-MIR395h,stu-MIR395i,stu-MIR395j,tae-MIR395a,tae-MIR395b,tcc-MIR395a,tcc-MIR395b,vun-MIR395,vvi-MIR395a,vvi-MIR395b,vvi-MIR395c,vvi-MIR395d,vvi-MIR395e,vvi-MIR395f,vvi-MIR395g,vvi-MIR395h,vvi-MIR395i,vvi-MIR395j,vvi-MIR395k,vvi-MIR395l,vvi-MIR395m,vvi-MIR395n,zma-MIR395a,zma-MIR395b,zma-MIR395c,zma-MIR395d,zma-MIR395e,zma-MIR395f,zma-MIR395g,zma-MIR395h,zma-MIR395i,zma-MIR395j,zma-MIR395k,zma-MIR395l,zma-MIR395m,zma-MIR395n,zma-MIR395o,zma-MIR395p |
| **MIR396-x** | 1 | aau-MIR396,aly-MIR396a,aly-MIR396b,ama-MIR396,amg-MIR396,aqc-MIR396a,aqc-MIR396b,ata-MIR396a,ata-MIR396b,ata-MIR396c,ata-MIR396d,ata-MIR396e,ath-MIR396a,ath-MIR396b,atr-MIR396a,atr-MIR396b,atr-MIR396c,atr-MIR396d,atr-MIR396e,bcy-MIR396a,bcy-MIR396b,bdi-MIR396a,bdi-MIR396b,bdi-MIR396c,bdi-MIR396d,bdi-MIR396e,bgy-MIR396a,bgy-MIR396b,bna-MIR396a,bra-MIR396,cca-MIR396a,cca-MIR396b,cca-MIR396c,ccl-MIR396,cme-MIR396a,cme-MIR396b,cme-MIR396c,cme-MIR396d,cme-MIR396e,cpa-MIR396,csi-MIR396a,csi-MIR396b,csi-MIR396c,dpr-MIR396,far-MIR396,ghr-MIR396a,ghr-MIR396b,gma-MIR396a,gma-MIR396b,gma-MIR396c,gma-MIR396d,gma-MIR396e,gma-MIR396f,gma-MIR396g,gma-MIR396h,gma-MIR396i,gma-MIR396j,gma-MIR396k,hbr-MIR396a,hbr-MIR396b,lja-MIR396,lus-MIR396a,lus-MIR396b,lus-MIR396c,lus-MIR396d,lus-MIR396e,mdm-MIR396a,mdm-MIR396b,mdm-MIR396c,mdm-MIR396d,mdm-MIR396e,mdm-MIR396f,mdm-MIR396g,mes-MIR396a,mes-MIR396b,mes-MIR396c,mes-MIR396d,mes-MIR396e,mes-MIR396f,mtr-MIR396a,mtr-MIR396b,mtr-MIR396c,nta-MIR396a,nta-MIR396b,nta-MIR396c,osa-MIR396a,osa-MIR396b,osa-MIR396c,osa-MIR396d,osa-MIR396e,osa-MIR396f,osa-MIR396g,osa-MIR396h,pab-MIR396a,pab-MIR396b,pab-MIR396c,pde-MIR396,ppe-MIR396a,ppe-MIR396b,pta-MIR396,ptc-MIR396a,ptc-MIR396b,ptc-MIR396c,ptc-MIR396d,ptc-MIR396e,ptc-MIR396f,ptc-MIR396g,rco-MIR396,sbi-MIR396a,sbi-MIR396b,sbi-MIR396c,sbi-MIR396d,sbi-MIR396e,sly-MIR396a,sly-MIR396b,smo-MIR396,sof-MIR396,ssl-MIR396,ssp-MIR396,stu-MIR396,tae-MIR396,tcc-MIR396a,tcc-MIR396b,tcc-MIR396c,tcc-MIR396d,tcc-MIR396e,vvi-MIR396a,vvi-MIR396b,vvi-MIR396c,vvi-MIR396d,zma-MIR396a,zma-MIR396b,zma-MIR396c,zma-MIR396d,zma-MIR396e,zma-MIR396f,zma-MIR396g,zma-MIR396h |
| **MIR396-y** | 1 | aau-MIR396,aly-MIR396a,aly-MIR396b,ama-MIR396,amg-MIR396,aqc-MIR396a,aqc-MIR396b,ata-MIR396a,ata-MIR396b,ata-MIR396c,ata-MIR396d,ata-MIR396e,ath-MIR396a,ath-MIR396b,atr-MIR396a,atr-MIR396b,atr-MIR396c,atr-MIR396d,atr-MIR396e,bcy-MIR396a,bcy-MIR396b,bdi-MIR396a,bdi-MIR396b,bdi-MIR396c,bdi-MIR396d,bdi-MIR396e,bgy-MIR396a,bgy-MIR396b,bna-MIR396a,bra-MIR396,cca-MIR396a,cca-MIR396b,cca-MIR396c,ccl-MIR396,cme-MIR396a,cme-MIR396b,cme-MIR396c,cme-MIR396d,cme-MIR396e,cpa-MIR396,csi-MIR396a,csi-MIR396b,csi-MIR396c,dpr-MIR396,far-MIR396,ghr-MIR396a,ghr-MIR396b,gma-MIR396a,gma-MIR396b,gma-MIR396c,gma-MIR396d,gma-MIR396e,gma-MIR396f,gma-MIR396g,gma-MIR396h,gma-MIR396i,gma-MIR396j,gma-MIR396k,hbr-MIR396a,hbr-MIR396b,lja-MIR396,lus-MIR396a,lus-MIR396b,lus-MIR396c,lus-MIR396d,lus-MIR396e,mdm-MIR396a,mdm-MIR396b,mdm-MIR396c,mdm-MIR396d,mdm-MIR396e,mdm-MIR396f,mdm-MIR396g,mes-MIR396a,mes-MIR396b,mes-MIR396c,mes-MIR396d,mes-MIR396e,mes-MIR396f,mtr-MIR396a,mtr-MIR396b,mtr-MIR396c,nta-MIR396a,nta-MIR396b,nta-MIR396c,osa-MIR396a,osa-MIR396b,osa-MIR396c,osa-MIR396d,osa-MIR396e,osa-MIR396f,osa-MIR396g,osa-MIR396h,pab-MIR396a,pab-MIR396b,pab-MIR396c,pde-MIR396,ppe-MIR396a,ppe-MIR396b,pta-MIR396,ptc-MIR396a,ptc-MIR396b,ptc-MIR396c,ptc-MIR396d,ptc-MIR396e,ptc-MIR396f,ptc-MIR396g,rco-MIR396,sbi-MIR396a,sbi-MIR396b,sbi-MIR396c,sbi-MIR396d,sbi-MIR396e,sly-MIR396a,sly-MIR396b,smo-MIR396,sof-MIR396,ssl-MIR396,ssp-MIR396,stu-MIR396,tae-MIR396,tcc-MIR396a,tcc-MIR396b,tcc-MIR396c,tcc-MIR396d,tcc-MIR396e,vvi-MIR396a,vvi-MIR396b,vvi-MIR396c,vvi-MIR396d,zma-MIR396a,zma-MIR396b,zma-MIR396c,zma-MIR396d,zma-MIR396e,zma-MIR396f,zma-MIR396g,zma-MIR396h |
| **MIR397-x** | 1 | aly-MIR397a,aly-MIR397b,ath-MIR397a,ath-MIR397b,atr-MIR397a,atr-MIR397b,bdi-MIR397a,bdi-MIR397b,bna-MIR397a,bna-MIR397b,cme-MIR397,csi-MIR397,dpr-MIR397,gma-MIR397a,gma-MIR397b,hvu-MIR397a,hvu-MIR397b,lja-MIR397,lus-MIR397a,lus-MIR397b,mdm-MIR397a,mdm-MIR397b,mes-MIR397,mtr-MIR397,nta-MIR397,osa-MIR397a,osa-MIR397b,pab-MIR397,ppe-MIR397,ptc-MIR397a,ptc-MIR397b,ptc-MIR397c,rco-MIR397,sbi-MIR397,sly-MIR397,ssl-MIR397,stu-MIR397,tae-MIR397,tcc-MIR397,vvi-MIR397a,zma-MIR397a,zma-MIR397b |
| **MIR398-x** | 1 | ahy-MIR398,aly-MIR398a,aly-MIR398b,aly-MIR398c,aqc-MIR398a,aqc-MIR398b,ata-MIR398f,ata-MIR398g,ath-MIR398a,ath-MIR398b,ath-MIR398c,atr-MIR398,bdi-MIR398a,bdi-MIR398b,bol-MIR398a,bra-MIR398,cca-MIR398,cme-MIR398a,cme-MIR398b,cpa-MIR398,csi-MIR398,ghr-MIR398,gma-MIR398a,gma-MIR398b,gma-MIR398c,gma-MIR398d,gra-MIR398,hbr-MIR398,lus-MIR398a,lus-MIR398b,lus-MIR398c,lus-MIR398d,lus-MIR398e,lus-MIR398f,mdm-MIR398a,mdm-MIR398b,mdm-MIR398c,mtr-MIR398a,mtr-MIR398b,mtr-MIR398c,nta-MIR398,osa-MIR398a,osa-MIR398b,ppe-MIR398a,ppe-MIR398b,pta-MIR398,ptc-MIR398a,ptc-MIR398b,ptc-MIR398c,rco-MIR398a,rco-MIR398b,sbi-MIR398,ssl-MIR398,stu-MIR398a,stu-MIR398b,tae-MIR398,tcc-MIR398a,tcc-MIR398b,vvi-MIR398a,vvi-MIR398b,vvi-MIR398c,zma-MIR398a,zma-MIR398b |
| **MIR398-y** | 1 | ahy-MIR398,aly-MIR398a,aly-MIR398b,aly-MIR398c,aqc-MIR398a,aqc-MIR398b,ata-MIR398f,ata-MIR398g,ath-MIR398a,ath-MIR398b,ath-MIR398c,atr-MIR398,bdi-MIR398a,bdi-MIR398b,bol-MIR398a,bra-MIR398,cca-MIR398,cme-MIR398a,cme-MIR398b,cpa-MIR398,csi-MIR398,ghr-MIR398,gma-MIR398a,gma-MIR398b,gma-MIR398c,gma-MIR398d,gra-MIR398,hbr-MIR398,lus-MIR398a,lus-MIR398b,lus-MIR398c,lus-MIR398d,lus-MIR398e,lus-MIR398f,mdm-MIR398a,mdm-MIR398b,mdm-MIR398c,mtr-MIR398a,mtr-MIR398b,mtr-MIR398c,nta-MIR398,osa-MIR398a,osa-MIR398b,ppe-MIR398a,ppe-MIR398b,pta-MIR398,ptc-MIR398a,ptc-MIR398b,ptc-MIR398c,rco-MIR398a,rco-MIR398b,sbi-MIR398,ssl-MIR398,stu-MIR398a,stu-MIR398b,tae-MIR398,tcc-MIR398a,tcc-MIR398b,vvi-MIR398a,vvi-MIR398b,vvi-MIR398c,zma-MIR398a,zma-MIR398b |
| **MIR398-z** | 1 | ahy-MIR398,aly-MIR398a,aly-MIR398b,aly-MIR398c,aqc-MIR398a,aqc-MIR398b,ata-MIR398f,ata-MIR398g,ath-MIR398a,ath-MIR398b,ath-MIR398c,atr-MIR398,bdi-MIR398a,bdi-MIR398b,bol-MIR398a,bra-MIR398,cca-MIR398,cme-MIR398a,cme-MIR398b,cpa-MIR398,csi-MIR398,ghr-MIR398,gma-MIR398a,gma-MIR398b,gma-MIR398c,gma-MIR398d,gra-MIR398,hbr-MIR398,lus-MIR398a,lus-MIR398b,lus-MIR398c,lus-MIR398d,lus-MIR398e,lus-MIR398f,mdm-MIR398a,mdm-MIR398b,mdm-MIR398c,mtr-MIR398a,mtr-MIR398b,mtr-MIR398c,nta-MIR398,osa-MIR398a,osa-MIR398b,ppe-MIR398a,ppe-MIR398b,pta-MIR398,ptc-MIR398a,ptc-MIR398b,ptc-MIR398c,rco-MIR398a,rco-MIR398b,sbi-MIR398,ssl-MIR398,stu-MIR398a,stu-MIR398b,tae-MIR398,tcc-MIR398a,tcc-MIR398b,vvi-MIR398a,vvi-MIR398b,vvi-MIR398c,zma-MIR398a,zma-MIR398b |
| **MIR399-y** | 1 | aly-MIR399a,aly-MIR399b,aly-MIR399c,aly-MIR399d,aly-MIR399e,aly-MIR399f,aly-MIR399g,aly-MIR399h,aly-MIR399i,aqc-MIR399,ata-MIR399a,ata-MIR399b,ath-MIR399a,ath-MIR399b,ath-MIR399c,ath-MIR399d,ath-MIR399e,ath-MIR399f,bdi-MIR399a,bdi-MIR399b,bdi-MIR399c,bdi-MIR399d,bna-MIR399a,bna-MIR399b,bna-MIR399c,cca-MIR399,cme-MIR399a,cme-MIR399b,cme-MIR399c,cme-MIR399d,cme-MIR399e,cme-MIR399f,cme-MIR399g,csi-MIR399a,csi-MIR399b,csi-MIR399c,csi-MIR399d,csi-MIR399e,ghr-MIR399a,ghr-MIR399b,ghr-MIR399c,ghr-MIR399d,ghr-MIR399e,gma-MIR399a,gma-MIR399b,gma-MIR399c,gma-MIR399d,gma-MIR399e,gma-MIR399f,gma-MIR399g,gma-MIR399h,gma-MIR399i,gra-MIR399,hvu-MIR399,lus-MIR399a,lus-MIR399b,lus-MIR399c,lus-MIR399d,lus-MIR399e,lus-MIR399f,lus-MIR399g,mdm-MIR399a,mdm-MIR399b,mdm-MIR399c,mdm-MIR399d,mdm-MIR399e,mdm-MIR399f,mdm-MIR399g,mdm-MIR399h,mdm-MIR399i,mdm-MIR399j,mes-MIR399a,mes-MIR399b,mes-MIR399c,mes-MIR399d,mes-MIR399e,mes-MIR399f,mes-MIR399g,mtr-MIR399a,mtr-MIR399b,mtr-MIR399c,mtr-MIR399d,mtr-MIR399e,mtr-MIR399f,mtr-MIR399g,mtr-MIR399h,mtr-MIR399i,mtr-MIR399j,mtr-MIR399k,mtr-MIR399l,mtr-MIR399m,mtr-MIR399n,mtr-MIR399o,mtr-MIR399p,mtr-MIR399q,mtr-MIR399r,mtr-MIR399s,mtr-MIR399t,nta-MIR399a,nta-MIR399b,nta-MIR399c,nta-MIR399d,nta-MIR399e,nta-MIR399f,nta-MIR399g,osa-MIR399a,osa-MIR399b,osa-MIR399c,osa-MIR399d,osa-MIR399e,osa-MIR399f,osa-MIR399g,osa-MIR399h,osa-MIR399i,osa-MIR399j,osa-MIR399k,ppe-MIR399a,ppe-MIR399b,ppe-MIR399c,ppe-MIR399d,ppe-MIR399e,ppe-MIR399f,ppe-MIR399g,ppe-MIR399h,ppe-MIR399i,ppe-MIR399j,ppe-MIR399k,ppe-MIR399l,ppe-MIR399m,ppe-MIR399n,ptc-MIR399a,ptc-MIR399b,ptc-MIR399c,ptc-MIR399d,ptc-MIR399e,ptc-MIR399f,ptc-MIR399g,ptc-MIR399h,ptc-MIR399i,ptc-MIR399j,pvu-MIR399a,rco-MIR399a,rco-MIR399b,rco-MIR399c,rco-MIR399d,rco-MIR399e,rco-MIR399f,sbi-MIR399a,sbi-MIR399b,sbi-MIR399c,sbi-MIR399d,sbi-MIR399e,sbi-MIR399f,sbi-MIR399g,sbi-MIR399h,sbi-MIR399i,sbi-MIR399j,sbi-MIR399k,sly-MIR399,ssl-MIR399,stu-MIR399a,stu-MIR399b,stu-MIR399c,stu-MIR399d,stu-MIR399e,stu-MIR399f,stu-MIR399g,stu-MIR399h,stu-MIR399i,stu-MIR399j,stu-MIR399k,stu-MIR399l,stu-MIR399m,stu-MIR399n,stu-MIR399o,tae-MIR399,tcc-MIR399a,tcc-MIR399b,tcc-MIR399c,tcc-MIR399d,tcc-MIR399e,tcc-MIR399f,tcc-MIR399g,tcc-MIR399h,tcc-MIR399i,vun-MIR399a,vun-MIR399b,vvi-MIR399a,vvi-MIR399b,vvi-MIR399c,vvi-MIR399d,vvi-MIR399e,vvi-MIR399f,vvi-MIR399g,vvi-MIR399h,vvi-MIR399i,zma-MIR399a,zma-MIR399b,zma-MIR399c,zma-MIR399d,zma-MIR399e,zma-MIR399f,zma-MIR399g,zma-MIR399h,zma-MIR399i,zma-MIR399j |
| **MIR403-y** | 1 | aly-MIR403a,aly-MIR403b,ath-MIR403,bna-MIR403,bra-MIR403,csi-MIR403,gma-MIR403a,gma-MIR403b,har-MIR403a,hpe-MIR403a,htu-MIR403a,htu-MIR403b,htu-MIR403c,htu-MIR403d,htu-MIR403e,mdm-MIR403a,mdm-MIR403b,mes-MIR403a,mes-MIR403b,ppe-MIR403,ptc-MIR403a,ptc-MIR403b,ptc-MIR403c,ptc-MIR403d,rco-MIR403a,rco-MIR403b,sly-MIR403,tcc-MIR403a,tcc-MIR403b,vvi-MIR403a,vvi-MIR403b,vvi-MIR403c,vvi-MIR403d,vvi-MIR403e,vvi-MIR403f |
| **MIR408-x** | 1 | ahy-MIR408,aly-MIR408,aqc-MIR408,ata-MIR408,ath-MIR408,bdi-MIR408,bra-MIR408,cca-MIR408,cme-MIR408,cpa-MIR408,csi-MIR408,dpr-MIR408,gma-MIR408a,gma-MIR408b,gma-MIR408c,gma-MIR408d,hbr-MIR408a,hbr-MIR408b,lja-MIR408,lus-MIR408a,mdm-MIR408a,mdm-MIR408b,mdm-MIR408c,mdm-MIR408d,mes-MIR408,mtr-MIR408,nta-MIR408,osa-MIR408,ppt-MIR408a,ppt-MIR408b,pta-MIR408,ptc-MIR408,rco-MIR408,sbi-MIR408,smo-MIR408,sof-MIR408a,sof-MIR408b,sof-MIR408c,sof-MIR408d,sof-MIR408e,ssp-MIR408a,ssp-MIR408d,stu-MIR408a,stu-MIR408b,tae-MIR408,vun-MIR408,vvi-MIR408,zma-MIR408a,zma-MIR408b |
| **MIR408-y** | 1 | ahy-MIR408,aly-MIR408,aqc-MIR408,ata-MIR408,ath-MIR408,bdi-MIR408,bra-MIR408,cca-MIR408,cme-MIR408,cpa-MIR408,csi-MIR408,dpr-MIR408,gma-MIR408a,gma-MIR408b,gma-MIR408c,gma-MIR408d,hbr-MIR408a,hbr-MIR408b,lja-MIR408,lus-MIR408a,mdm-MIR408a,mdm-MIR408b,mdm-MIR408c,mdm-MIR408d,mes-MIR408,mtr-MIR408,nta-MIR408,osa-MIR408,ppt-MIR408a,ppt-MIR408b,pta-MIR408,ptc-MIR408,rco-MIR408,sbi-MIR408,smo-MIR408,sof-MIR408a,sof-MIR408b,sof-MIR408c,sof-MIR408d,sof-MIR408e,ssp-MIR408a,ssp-MIR408d,stu-MIR408a,stu-MIR408b,tae-MIR408,vun-MIR408,vvi-MIR408,zma-MIR408a,zma-MIR408b |
| **MIR4243-x** | 1 | aly-MIR4243,ath-MIR4243 |
| **MIR4414-x** | 1 | gma-MIR4414,mtr-MIR4414a,mtr-MIR4414b |
| **MIR472-y** | 1 | aly-MIR472,ath-MIR472,csi-MIR472,ptc-MIR472a,ptc-MIR472b |
| **MIR473-x** | 1 | csi-MIR473,ptc-MIR473a,ptc-MIR473b |
| **MIR474-x** | 1 | ptc-MIR474a,ptc-MIR474b,ptc-MIR474c |
| **MIR477-x** | 1 | aqc-MIR477a,aqc-MIR477b,aqc-MIR477c,aqc-MIR477d,aqc-MIR477e,aqc-MIR477f,aqc-MIR477g,atr-MIR477,cme-MIR477a,cme-MIR477b,cpa-MIR477,csi-MIR477a,csi-MIR477b,csi-MIR477c,gra-MIR477,mdm-MIR477a,mdm-MIR477b,mes-MIR477a,mes-MIR477b,mes-MIR477c,mes-MIR477d,mes-MIR477e,mes-MIR477f,mes-MIR477g,mes-MIR477h,mes-MIR477i,nta-MIR477a,nta-MIR477b,ppe-MIR477,ppe-MIR477a,ppe-MIR477b,ppt-MIR477a,ppt-MIR477b,ppt-MIR477c,ppt-MIR477d,ppt-MIR477e,ppt-MIR477f,ppt-MIR477g,ppt-MIR477h,ptc-MIR477a,ptc-MIR477b,ptc-MIR477c,ptc-MIR477d,sly-MIR477,stu-MIR477a,stu-MIR477b,vvi-MIR477a,vvi-MIR477b |
| **MIR479-x** | 1 | csi-MIR479,ghr-MIR479,nta-MIR479a,nta-MIR479b,stu-MIR479,vvi-MIR479 |
| **MIR482-x** | 1 | aqc-MIR482a,aqc-MIR482b,aqc-MIR482c,csi-MIR482a,csi-MIR482b,csi-MIR482c,ghr-MIR482a,ghr-MIR482b,gma-MIR482a,gma-MIR482b,gma-MIR482c,gma-MIR482d,gma-MIR482e,gra-MIR482,gra-MIR482c,gra-MIR482d,gso-MIR482a,gso-MIR482b,hbr-MIR482a,hbr-MIR482b,mdm-MIR482a,mdm-MIR482b,mdm-MIR482c,mdm-MIR482d,mes-MIR482,mtr-MIR482,nta-MIR482a,nta-MIR482b,nta-MIR482c,nta-MIR482d,pab-MIR482a,pab-MIR482b,pab-MIR482c,pab-MIR482d,pde-MIR482a,pde-MIR482b,pde-MIR482c,pde-MIR482d,pgi-MIR482a,pgi-MIR482b,ppe-MIR482a,ppe-MIR482b,ppe-MIR482c,ppe-MIR482d,ppe-MIR482e,ppe-MIR482f,pta-MIR482a,pta-MIR482b,pta-MIR482c,pta-MIR482d,ptc-MIR482a,ptc-MIR482b,ptc-MIR482c,ptc-MIR482d,pvu-MIR482,sly-MIR482a,sly-MIR482b,sly-MIR482c,sly-MIR482d,sly-MIR482e,stu-MIR482a,stu-MIR482b,stu-MIR482c,stu-MIR482d,stu-MIR482e,vun-MIR482,vvi-MIR482,zma-MIR482 |
| **MIR482-y** | 1 | aqc-MIR482a,aqc-MIR482b,aqc-MIR482c,csi-MIR482a,csi-MIR482b,csi-MIR482c,ghr-MIR482a,ghr-MIR482b,gma-MIR482a,gma-MIR482b,gma-MIR482c,gma-MIR482d,gma-MIR482e,gra-MIR482,gra-MIR482c,gra-MIR482d,gso-MIR482a,gso-MIR482b,hbr-MIR482a,hbr-MIR482b,mdm-MIR482a,mdm-MIR482b,mdm-MIR482c,mdm-MIR482d,mes-MIR482,mtr-MIR482,nta-MIR482a,nta-MIR482b,nta-MIR482c,nta-MIR482d,pab-MIR482a,pab-MIR482b,pab-MIR482c,pab-MIR482d,pde-MIR482a,pde-MIR482b,pde-MIR482c,pde-MIR482d,pgi-MIR482a,pgi-MIR482b,ppe-MIR482a,ppe-MIR482b,ppe-MIR482c,ppe-MIR482d,ppe-MIR482e,ppe-MIR482f,pta-MIR482a,pta-MIR482b,pta-MIR482c,pta-MIR482d,ptc-MIR482a,ptc-MIR482b,ptc-MIR482c,ptc-MIR482d,pvu-MIR482,sly-MIR482a,sly-MIR482b,sly-MIR482c,sly-MIR482d,sly-MIR482e,stu-MIR482a,stu-MIR482b,stu-MIR482c,stu-MIR482d,stu-MIR482e,vun-MIR482,vvi-MIR482,zma-MIR482 |
| **MIR482-z** | 1 | aqc-MIR482a,aqc-MIR482b,aqc-MIR482c,csi-MIR482a,csi-MIR482b,csi-MIR482c,ghr-MIR482a,ghr-MIR482b,gma-MIR482a,gma-MIR482b,gma-MIR482c,gma-MIR482d,gma-MIR482e,gra-MIR482,gra-MIR482c,gra-MIR482d,gso-MIR482a,gso-MIR482b,hbr-MIR482a,hbr-MIR482b,mdm-MIR482a,mdm-MIR482b,mdm-MIR482c,mdm-MIR482d,mes-MIR482,mtr-MIR482,nta-MIR482a,nta-MIR482b,nta-MIR482c,nta-MIR482d,pab-MIR482a,pab-MIR482b,pab-MIR482c,pab-MIR482d,pde-MIR482a,pde-MIR482b,pde-MIR482c,pde-MIR482d,pgi-MIR482a,pgi-MIR482b,ppe-MIR482a,ppe-MIR482b,ppe-MIR482c,ppe-MIR482d,ppe-MIR482e,ppe-MIR482f,pta-MIR482a,pta-MIR482b,pta-MIR482c,pta-MIR482d,ptc-MIR482a,ptc-MIR482b,ptc-MIR482c,ptc-MIR482d,pvu-MIR482,sly-MIR482a,sly-MIR482b,sly-MIR482c,sly-MIR482d,sly-MIR482e,stu-MIR482a,stu-MIR482b,stu-MIR482c,stu-MIR482d,stu-MIR482e,vun-MIR482,vvi-MIR482,zma-MIR482 |
| **MIR4995-x** | 1 | gma-MIR4995 |
| **MIR5015-x** | 1 | ath-MIR5015 |
| **MIR5026-y** | 1 | ath-MIR5026 |
| **MIR5054-y** | 1 | bdi-MIR5054 |
| **MIR5059-x** | 1 | bdi-MIR5059 |
| **MIR5072-x** | 1 | osa-MIR5072 |
| **MIR5077-x** | 1 | osa-MIR5077 |
| **MIR5083-y** | 1 | osa-MIR5083 |
| **MIR5139-x** | 1 | rgl-MIR5139 |
| **MIR5147-y** | 1 | osa-MIR5147 |
| **MIR5168-y** | 1 | ata-MIR5168 |
| **MIR5185-y** | 1 | bdi-MIR5185a,bdi-MIR5185b,bdi-MIR5185c,bdi-MIR5185d,bdi-MIR5185e,bdi-MIR5185f,bdi-MIR5185g,bdi-MIR5185h,bdi-MIR5185i,bdi-MIR5185j,bdi-MIR5185k,bdi-MIR5185l,bdi-MIR5185m |
| **MIR5205-x** | 1 | mtr-MIR5205a,mtr-MIR5205b,mtr-MIR5205c,mtr-MIR5205d |
| **MIR5211-x** | 1 | cpa-MIR5211,mtr-MIR5211 |
| **MIR5225-x** | 1 | gma-MIR5225,mdm-MIR5225a,mdm-MIR5225b,mdm-MIR5225c,mtr-MIR5225a,mtr-MIR5225b,mtr-MIR5225c,ppe-MIR5225 |
| **MIR5274-y** | 1 | mtr-MIR5274a,mtr-MIR5274b |
| **MIR5281-y** | 1 | bdi-MIR5281a,bdi-MIR5281b,mtr-MIR5281a,mtr-MIR5281b,mtr-MIR5281c,mtr-MIR5281d,mtr-MIR5281e,mtr-MIR5281f |
| **MIR530-x** | 1 | aqc-MIR530,bdi-MIR530a,bdi-MIR530b,cme-MIR530a,cme-MIR530b,csi-MIR530a,csi-MIR530b,gma-MIR530a,gma-MIR530b,gma-MIR530c,gma-MIR530d,gma-MIR530e,gra-MIR530a,gra-MIR530b,htu-MIR530,lus-MIR530a,lus-MIR530b,mes-MIR530a,mes-MIR530b,mtr-MIR530,osa-MIR530,ppe-MIR530,ptc-MIR530a,ptc-MIR530b,stu-MIR530,tae-MIR530,tcc-MIR530a,tcc-MIR530b |
| **MIR535-x** | 1 | aqc-MIR535,atr-MIR535,cpa-MIR535,csi-MIR535,mdm-MIR535a,mdm-MIR535b,mdm-MIR535c,mdm-MIR535d,mes-MIR535a,mes-MIR535b,osa-MIR535,pab-MIR535,ppe-MIR535a,ppe-MIR535b,ppt-MIR535a,ppt-MIR535b,ppt-MIR535c,ppt-MIR535d,rco-MIR535,tcc-MIR535,vvi-MIR535a,vvi-MIR535b,vvi-MIR535c |
| **MIR535-y** | 1 | aqc-MIR535,atr-MIR535,cpa-MIR535,csi-MIR535,mdm-MIR535a,mdm-MIR535b,mdm-MIR535c,mdm-MIR535d,mes-MIR535a,mes-MIR535b,osa-MIR535,pab-MIR535,ppe-MIR535a,ppe-MIR535b,ppt-MIR535a,ppt-MIR535b,ppt-MIR535c,ppt-MIR535d,rco-MIR535,tcc-MIR535,vvi-MIR535a,vvi-MIR535b,vvi-MIR535c |
| **MIR535-z** | 1 | aqc-MIR535,atr-MIR535,cpa-MIR535,csi-MIR535,mdm-MIR535a,mdm-MIR535b,mdm-MIR535c,mdm-MIR535d,mes-MIR535a,mes-MIR535b,osa-MIR535,pab-MIR535,ppe-MIR535a,ppe-MIR535b,ppt-MIR535a,ppt-MIR535b,ppt-MIR535c,ppt-MIR535d,rco-MIR535,tcc-MIR535,vvi-MIR535a,vvi-MIR535b,vvi-MIR535c |
| **MIR5368-y** | 1 | gma-MIR5368 |
| **MIR5530-x** | 1 | osa-MIR5530 |
| **MIR5538-x** | 1 | osa-MIR5538 |
| **MIR5563-x** | 1 | mtr-MIR5563 |
| **MIR5628-x** | 1 | ath-MIR5628 |
| **MIR5632-y** | 1 | ath-MIR5632 |
| **MIR5641-y** | 1 | ath-MIR5641 |
| **MIR5776-x** | 1 | gma-MIR5776 |
| **MIR6034-y** | 1 | bna-MIR6034 |
| **MIR6106-y** | 1 | cca-MIR6106 |
| **MIR6135-x** | 1 | pgi-MIR6135a,pgi-MIR6135b,pgi-MIR6135c,pgi-MIR6135d,pgi-MIR6135e,pgi-MIR6135f,pgi-MIR6135g,pgi-MIR6135h,pgi-MIR6135i,pgi-MIR6135j,pgi-MIR6135k |
| **MIR6138-x** | 1 | pgi-MIR6138 |
| **MIR6173-y** | 1 | hbr-MIR6173 |
| **MIR6277-y** | 1 | ppe-MIR6277 |
| **MIR6284-y** | 1 | ppe-MIR6284 |
| **MIR6293-x** | 1 | ppe-MIR6293 |
| **MIR6300-y** | 1 | gma-MIR6300 |
| **MIR6445-x** | 1 | ptc-MIR6445a,ptc-MIR6445b |
| **MIR6478-x** | 1 | ptc-MIR6478 |
| **MIR6480-y** | 1 | ptc-MIR6480 |
| **MIR6483-y** | 1 | hbr-MIR6483 |
| **MIR6484-y** | 1 | hbr-MIR6484 |
| **MIR6485-x** | 1 | hbr-MIR6485 |
| **MIR7508-x** | 1 | ghr-MIR7508 |
| **MIR7534-x** | 1 | lja-MIR7534 |
| **MIR7693-y** | 1 | osa-MIR7693 |
| **MIR7726-x** | 1 | bdi-MIR7726a,bdi-MIR7726b |
| **MIR7732-x** | 1 | bdi-MIR7732 |
| **MIR7741-y** | 1 | bdi-MIR7741 |
| **MIR7747-x** | 1 | bdi-MIR7747 |
| **MIR7752-y** | 1 | bdi-MIR7752 |
| **MIR7767-x** | 1 | bdi-MIR7767 |
| **MIR7779-y** | 1 | bdi-MIR7779 |
| **MIR7782-y** | 1 | bdi-MIR7782 |
| **MIR7783-y** | 1 | bdi-MIR7783 |
| **MIR7814-x** | 1 | ptc-MIR7814 |
| **MIR8005-x** | 1 | stu-MIR8005a,stu-MIR8005b,stu-MIR8005c |
| **MIR8009-y** | 1 | stu-MIR8009 |
| **MIR8022-x** | 1 | stu-MIR8022 |
| **MIR8026-x** | 1 | stu-MIR8026 |
| **MIR8032-y** | 1 | stu-MIR8032a,stu-MIR8032b,stu-MIR8032c,stu-MIR8032d,stu-MIR8032e,stu-MIR8032f,stu-MIR8032g |
| **MIR8044-x** | 1 | stu-MIR8044 |
| **MIR8155-y** | 1 | cpa-MIR8155 |
| **MIR8175-y** | 1 | ath-MIR8175 |
| **MIR8177-x** | 1 | ath-MIR8177 |
| **MIR8180-y** | 1 | ath-MIR8180 |
| **MIR827-x** | 1 | aly-MIR827,ath-MIR827,bdi-MIR827,csi-MIR827,ghr-MIR827a,ghr-MIR827b,ghr-MIR827c,gra-MIR827b,mdm-MIR827,mes-MIR827,nta-MIR827,osa-MIR827,ppe-MIR827,ptc-MIR827,ssp-MIR827,stu-MIR827,tcc-MIR827,zma-MIR827 |
| **MIR827-y** | 1 | aly-MIR827,ath-MIR827,bdi-MIR827,csi-MIR827,ghr-MIR827a,ghr-MIR827b,ghr-MIR827c,gra-MIR827b,mdm-MIR827,mes-MIR827,nta-MIR827,osa-MIR827,ppe-MIR827,ptc-MIR827,ssp-MIR827,stu-MIR827,tcc-MIR827,zma-MIR827 |
| **MIR829-x** | 1 | aly-MIR829,ath-MIR829 |
| **MIR845-x** | 1 | aly-MIR845a,aly-MIR845b,ath-MIR845a,ath-MIR845b,bdi-MIR845,cme-MIR845,vvi-MIR845a,vvi-MIR845b,vvi-MIR845c,vvi-MIR845d,vvi-MIR845e |
| **MIR845-y** | 1 | aly-MIR845a,aly-MIR845b,ath-MIR845a,ath-MIR845b,bdi-MIR845,cme-MIR845,vvi-MIR845a,vvi-MIR845b,vvi-MIR845c,vvi-MIR845d,vvi-MIR845e |
| **MIR845-z** | 1 | aly-MIR845a,aly-MIR845b,ath-MIR845a,ath-MIR845b,bdi-MIR845,cme-MIR845,vvi-MIR845a,vvi-MIR845b,vvi-MIR845c,vvi-MIR845d,vvi-MIR845e |
| **MIR846-x** | 1 | aly-MIR846,ath-MIR846 |
| **MIR847-x** | 1 | aly-MIR847,ath-MIR847 |
| **MIR8551-y** | 1 | atr-MIR8551 |
| **MIR8565-x** | 1 | atr-MIR8565a,atr-MIR8565b,atr-MIR8565c,atr-MIR8565d,atr-MIR8565e,atr-MIR8565f,atr-MIR8565g,atr-MIR8565h |
| **MIR8569-y** | 1 | atr-MIR8569 |
| **MIR858-x** | 1 | aly-MIR858,ath-MIR858a,ath-MIR858b,cme-MIR858,mdm-MIR858,ppe-MIR858 |
| **MIR858-y** | 1 | aly-MIR858,ath-MIR858a,ath-MIR858b,cme-MIR858,mdm-MIR858,ppe-MIR858 |
| **MIR859-x** | 1 | aly-MIR859,ath-MIR859 |
| **MIR863-x** | 1 | ath-MIR863 |
| **MIR8643-x** | 1 | gra-MIR8643a,gra-MIR8643b |
| **MIR8663-x** | 1 | gra-MIR8663a,gra-MIR8663b |
| **MIR8762-x** | 1 | gra-MIR8762a,gra-MIR8762b,gra-MIR8762c,gra-MIR8762d,gra-MIR8762e |
| **MIR894-x** | 1 | ppt-MIR894 |
| **MIR9408-y** | 1 | bol-MIR9408,bra-MIR9408 |
| **MIR9654-y** | 1 | tae-MIR9654a,tae-MIR9654b |
| **MIR9726-y** | 1 | gma-MIR9726 |
| **MIR9773-y** | 1 | tae-MIR9773 |
| **MIR9897-y** | 1 | cre-MIR9897 |
