# Supplementary material for: MicroRNAome Profile of Euphorbia kansui in Response to Methyl Jasmonate
Source: Int J Mol Sci. 2019 Mar 13;20(6):1267. doi: 10.3390/ijms20061267 (PMC6471261; doi:10.3390/ijms20061267)
Supplement: Supplementary file 1 [file ijms-20-01267-s001.zip › ijms-432617-SI-final/Table S5.doc]

**Additional File5 Table S5: The pairs of miRNA and their target mRNA in response to MeJA treatment of *Euphorbia kansui***

| **Pairwise** | **RNA_id** | **MIR_id** | **Correlation** | **RNA_exp**  **CK T1 T2 T3** | **MIR_exp**  **CK T1 T2 T3** | **Symbol** |
| --- | --- | --- | --- | --- | --- | --- |
| **RNA_profile0-vs-MIR_profile5** | Unigene087713 | novel-m0039-3p | -0.8 | 79.16,21.19,14.225,10.03 | 2.6338,0.0100,4.0629,16.9817 | Unigene087713_08 |
| **RNA_profile0-vs-MIR_profile5** | Unigene083856 | MIR9726-y | -0.6 | 5.09,3.995,2.54,1.91 | 0.9243,0.0100,2.8970,1.2745 | Unigene083856_04 |
| **RNA_profile0-vs-MIR_profile5** | Unigene090070 | novel-m0593-5p | -0.8 | 1.38,0.86,0.195,0.11 | 0.9243,0.0100,1.2062,4.6026 | Unigene090070_01 |
| **RNA_profile0-vs-MIR_profile5** | Unigene090070 | novel-m0123-5p | -0.8 | 1.38,0.86,0.195,0.11 | 0.9243,0.0100,1.2062,4.6026 | Unigene090070_01 |
| **RNA_profile0-vs-MIR_profile5** | Unigene090070 | novel-m0395-5p | -0.8 | 1.38,0.86,0.195,0.11 | 0.9243,0.0100,1.2062,4.6026 | Unigene090070_01 |
| **RNA_profile0-vs-MIR_profile5** | Unigene090070 | novel-m0027-5p | -0.8 | 1.38,0.86,0.195,0.11 | 0.9243,0.0100,1.2062,4.6026 | Unigene090070_01 |
| **RNA_profile0-vs-MIR_profile5** | Unigene081611 | MIR8175-y | -1 | 5.09,5.165,3.545,2.295 | 3.5531,0.0100,5.0926,6.7054 | Unigene081611_02 |
| **RNA_profile0-vs-MIR_profile6** | Unigene076354 | novel-m0234-3p | -0.8 | 1.12,0.35,0.33,0.07 | 86.0970,21.2492,96.0264,210.7218 | Unigene076354_01 |
| **RNA_profile0-vs-MIR_profile12** | Unigene087713 | novel-m0628-3p | -0.948683298 | 79.16,21.19,14.225,10.03 | 0.0100,0.0100,2.3317,8.4113 | Unigene087713_08 |
| **RNA_profile0-vs-MIR_profile12** | Unigene087713 | novel-m0626-3p | -0.948683298 | 79.16,21.19,14.225,10.03 | 0.0100,0.0100,2.3317,8.4113 | Unigene087713_08 |
| **RNA_profile1-vs-MIR_profile9** | Unigene093718 | novel-m0088-5p | -0.8 | 1.8,0.96,0.405,0.995 | 2.6338,3.5756,4.1335,0.0100 | Unigene093718_03 |
| **RNA_profile1-vs-MIR_profile16** | Unigene075280 | novel-m0210-5p | -0.737864787 | 2.095,1.135,0.47,1.4 | 0.0100,28.4254,1.2062,0.0100 | Unigene075280_01 |
| **RNA_profile1-vs-MIR_profile16** | Unigene048028 | MIR7783-y | -0.632455532 | 27.19,4.135,1.02,2.93 | 0.0100,2.3854,2.8970,0.0100 | Unigene048028_02 |
| **RNA_profile2-vs-MIR_profile14** | Unigene046417 | novel-m0312-3p | -0.774596669 | 591.605,94.345,96.555,120.02 | 0.0100,10.6421,0.0100,0.0100 | Unigene046417_04 |
| **RNA_profile2-vs-MIR_profile15** | Unigene029798 | novel-m0221-5p | -0.6 | 1.11,0.095,0.32,0.225 | 5.2676,9.4170,4.6282,10.9111 | Unigene029798_01 |
| **RNA_profile2-vs-MIR_profile15** | Unigene059048 | novel-m0376-3p | -1 | 1.185,0.49,0.595,0.56 | 27.2641,88.6374,30.4538,80.5043 | Unigene059048_02 |
| **RNA_profile2-vs-MIR_profile15** | Unigene059800 | novel-m0221-5p | -0.6 | 0.86,0.285,0.495,0.445 | 5.2676,9.4170,4.6282,10.9111 | Unigene059800_01 |
| **RNA_profile2-vs-MIR_profile15** | Unigene025534 | novel-m0376-3p | -0.8 | 16.745,4.5,6.13,7.655 | 27.2641,88.6374,30.4538,80.5043 | Unigene025534_04 |
| **RNA_profile2-vs-MIR_profile15** | Unigene030741 | novel-m0139-3p | -0.948683298 | 2.56,0.705,1.16,1.03 | 0.0100,3.5756,0.0100,2.9361 | Unigene030741_01 |
| **RNA_profile2-vs-MIR_profile15** | Unigene028043 | novel-m0376-3p | -0.8 | 74.145,35.9,44.48,46.115 | 27.2641,88.6374,30.4538,80.5043 | Unigene028043_02 |
| **RNA_profile2-vs-MIR_profile17** | Unigene041856 | novel-m0072-5p | -0.8 | 65.785,22.405,30,31.905 | 0.0100,35.4271,8.6205,11.1489 | Unigene041856_04 |
| **RNA_profile2-vs-MIR_profile17** | Unigene041856 | novel-m0073-5p | -0.8 | 65.785,22.405,30,31.905 | 0.0100,35.4271,8.6205,11.1489 | Unigene041856_04 |
| **RNA_profile2-vs-MIR_profile17** | Unigene041856 | novel-m0071-5p | -0.8 | 65.785,22.405,30,31.905 | 0.0100,35.4271,8.6205,11.1489 | Unigene041856_04 |
| **RNA_profile2-vs-MIR_profile17** | Unigene046251 | novel-m0072-5p | -1 | 6.24,2.355,3.685,3.43 | 0.0100,35.4271,8.6205,11.1489 | Unigene046251_02 |
| **RNA_profile2-vs-MIR_profile17** | Unigene046251 | novel-m0073-5p | -1 | 6.24,2.355,3.685,3.43 | 0.0100,35.4271,8.6205,11.1489 | Unigene046251_02 |
| **RNA_profile2-vs-MIR_profile17** | Unigene046251 | novel-m0071-5p | -1 | 6.24,2.355,3.685,3.43 | 0.0100,35.4271,8.6205,11.1489 | Unigene046251_02 |
| **RNA_profile3-vs-MIR_profile9** | Unigene008348 | novel-m0152-3p | -1 | 1.205,0.57,0.47,1.855 | 2.6338,4.7658,6.5005,0.0100 | Unigene008348_01 |
| **RNA_profile3-vs-MIR_profile9** | Unigene008348 | MIR6480-y | -1 | 1.205,0.57,0.47,1.855 | 1.8436,2.3854,3.5329,0.0100 | Unigene008348_01 |
| **RNA_profile3-vs-MIR_profile9** | Unigene093115 | novel-m0152-3p | -0.8 | 1.465,0.565,0.425,1.07 | 2.6338,4.7658,6.5005,0.0100 | Unigene093115_02 |
| **RNA_profile3-vs-MIR_profile9** | Unigene058149 | novel-m0180-5p | -0.6 | 1.09,0.26,0.255,0.62 | 1.8436,2.3854,2.3317,0.0100 | Unigene058149_01 |
| **RNA_profile3-vs-MIR_profile9** | Unigene043451 | novel-m0225-5p | -0.737864787 | 0.51,0.000500,0.000500,0.485 | 0.9243,3.5756,4.0982,0.0100 | Unigene043451_01 |
| **RNA_profile3-vs-MIR_profile9** | Unigene053700 | novel-m0225-5p | -0.6 | 193.955,45.69,53.09,131.62 | 0.9243,3.5756,4.0982,0.0100 | Unigene053700_06 |
| **RNA_profile3-vs-MIR_profile9** | Unigene033384 | novel-m0152-3p | -0.6 | 4.675,1.045,1.39,2.95 | 2.6338,4.7658,6.5005,0.0100 | Unigene033384_02 |
| **RNA_profile3-vs-MIR_profile11** | Unigene012777 | MIR2916-x | -0.6 | 814.7,374.745,383.36,569.19 | 652.4482,1059.0375,1967.9084,595.6163 | Unigene012777_11 |
| **RNA_profile3-vs-MIR_profile14** | Unigene033384 | novel-m0187-3p | -0.8 | 4.675,1.045,1.39,2.95 | 16.4302,35.4271,20.0668,14.8789 | Unigene033384_02 |
| **RNA_profile3-vs-MIR_profile14** | Unigene003453 | novel-m0434-3p | -0.774596669 | 4.585,0.925,1.42,2.285 | 0.0100,47.3988,0.0100,0.0100 | Unigene003453_01 |
| **RNA_profile3-vs-MIR_profile14** | Unigene093115 | novel-m0187-3p | -0.6 | 1.465,0.565,0.425,1.07 | 16.4302,35.4271,20.0668,14.8789 | Unigene093115_02 |
| **RNA_profile3-vs-MIR_profile14** | Unigene008348 | novel-m0187-3p | -0.8 | 1.205,0.57,0.47,1.855 | 16.4302,35.4271,20.0668,14.8789 | Unigene008348_01 |
| **RNA_profile3-vs-MIR_profile14** | Unigene068557 | novel-m0274-5p | -0.8 | 1.755,0.755,0.76,1.24 | 36.7926,93.3284,43.4548,44.6759 | Unigene068557_01 |
| **RNA_profile3-vs-MIR_profile16** | Unigene044885 | MIR7783-y | -0.948683298 | 9.545,5.335,4.405,8.99 | 0.0100,2.3854,2.8970,0.0100 | Unigene044885_02 |
| **RNA_profile3-vs-MIR_profile16** | Unigene002450 | MIR7783-y | -0.948683298 | 0.98,0.145,0.05,0.875 | 0.0100,2.3854,2.8970,0.0100 | Unigene002450_01 |
| **RNA_profile3-vs-MIR_profile16** | Unigene000271 | MIR7783-y | -0.948683298 | 10.12,5.67,5.03,10.035 | 0.0100,2.3854,2.8970,0.0100 | Unigene000271_04 |
| **RNA_profile3-vs-MIR_profile16** | Unigene090068 | novel-m0210-5p | -0.737864787 | 1.55,1.055,0.725,1.51 | 0.0100,28.4254,1.2062,0.0100 | Unigene090068_01 |
| **RNA_profile3-vs-MIR_profile16** | Unigene041728 | MIR7783-y | -0.948683298 | 0.9,0.41,0.3,1.195 | 0.0100,2.3854,2.8970,0.0100 | Unigene041728_03 |
| **RNA_profile3-vs-MIR_profile16** | Unigene003219 | novel-m0205-5p | -0.737864787 | 36.93,25.175,17.155,45.73 | 0.0100,9.4518,1.2062,0.0100 | Unigene003219_03 |
| **RNA_profile4-vs-MIR_profile14** | Unigene012518 | MIR479-x | -0.8 | 1.35,0.04,0.25,0.035 | 143.3551,476.6568,233.8490,272.7371 | Unigene012518_01 |
| **RNA_profile4-vs-MIR_profile14** | Unigene014261 | novel-m0294-5p | -0.8 | 1.08,0.11,1.135,0.115 | 1.2599,99.2446,3.4976,6.2691 | Unigene014261_01 |
| **RNA_profile4-vs-MIR_profile14** | Unigene028383 | MIR397-x | -0.774596669 | 11.94,1.935,11.17,3.43 | 0.0100,7.0715,0.0100,0.0100 | Unigene028383_04 |
| **RNA_profile4-vs-MIR_profile14** | Unigene009790 | MIR398-y | -1 | 3.185,0.27,3.94,0.305 | 6137.4225,47283.6161,5071.3429,11697.7448 | Unigene009790_01 |
| **RNA_profile4-vs-MIR_profile14** | Unigene048510 | MIR397-x | -0.774596669 | 10.845,4.895,9.36,6.73 | 0.0100,7.0715,0.0100,0.0100 | Unigene048510_07 |
| **RNA_profile4-vs-MIR_profile14** | Unigene064667 | MIR858-x | -0.8 | 0.835,0.065,0.465,0.03 | 646.9534,2321.2490,678.8600,882.4793 | Unigene064667_01 |
| **RNA_profile4-vs-MIR_profile14** | Unigene011249 | novel-m0294-5p | -0.8 | 2.815,1.27,4.46,1.78 | 1.2599,99.2446,3.4976,6.2691 | Unigene011249_02 |
| **RNA_profile4-vs-MIR_profile14** | Unigene036516 | MIR397-x | -0.544331054 | 4.025,0.000500,0.095,0.000500 | 0.0100,7.0715,0.0100,0.0100 | Unigene036516_01 |
| **RNA_profile4-vs-MIR_profile15** | Unigene014261 | novel-m0528-3p | -0.948683298 | 1.08,0.11,1.135,0.115 | 0.0100,96.8642,0.0100,1.2745 | Unigene014261_01 |
| **RNA_profile4-vs-MIR_profile15** | Unigene083387 | novel-m0595-3p | -0.8 | 8.71,2.29,6.045,3.505 | 18.4802,65.0077,36.2126,99.4690 | Unigene083387_03 |
| **RNA_profile4-vs-MIR_profile15** | Unigene053745 | novel-m0139-3p | -0.737864787 | 2.9,0.59,2.285,0.24 | 0.0100,3.5756,0.0100,2.9361 | Unigene053745_01 |
| **RNA_profile4-vs-MIR_profile15** | Unigene093117 | novel-m0376-3p | -1 | 3.23,1,2.6,1.705 | 27.2641,88.6374,30.4538,80.5043 | Unigene093117_03 |
| **RNA_profile4-vs-MIR_profile15** | Unigene011249 | novel-m0528-3p | -0.948683298 | 2.815,1.27,4.46,1.78 | 0.0100,96.8642,0.0100,1.2745 | Unigene011249_02 |
| **RNA_profile4-vs-MIR_profile15** | Unigene093885 | novel-m0595-3p | -0.6 | 3.985,1.845,5.62,2.54 | 18.4802,65.0077,36.2126,99.4690 | Unigene093885_02 |
| **RNA_profile4-vs-MIR_profile15** | Unigene020217 | novel-m0247-5p | -0.8 | 4.225,1.525,9.965,2.48 | 53.1721,229.8227,75.7104,169.0999 | Unigene020217_03 |
| **RNA_profile4-vs-MIR_profile15** | Unigene053756 | novel-m0139-3p | -0.888888889 | 0.47,0.000500,1.71,0.000500 | 0.0100,3.5756,0.0100,2.9361 | Unigene053756_01 |
| **RNA_profile4-vs-MIR_profile17** | Unigene028383 | novel-m0065-5p | -1 | 11.94,1.935,11.17,3.43 | 0.0100,14.1430,5.9352,7.1418 | Unigene028383_04 |
| **RNA_profile4-vs-MIR_profile17** | Unigene028383 | novel-m0067-5p | -1 | 11.94,1.935,11.17,3.43 | 0.0100,14.1430,5.9352,7.1418 | Unigene028383_04 |
| **RNA_profile4-vs-MIR_profile17** | Unigene011841 | novel-m0073-5p | -1 | 1.32,0.085,0.56,0.275 | 0.0100,35.4271,8.6205,11.1489 | Unigene011841_01 |
| **RNA_profile4-vs-MIR_profile17** | Unigene011841 | novel-m0071-5p | -1 | 1.32,0.085,0.56,0.275 | 0.0100,35.4271,8.6205,11.1489 | Unigene011841_01 |
| **RNA_profile4-vs-MIR_profile17** | Unigene011841 | novel-m0072-5p | -1 | 1.32,0.085,0.56,0.275 | 0.0100,35.4271,8.6205,11.1489 | Unigene011841_01 |
| **RNA_profile4-vs-MIR_profile17** | Unigene038894 | novel-m0330-3p | -1 | 46.755,16.83,35.13,22.12 | 0.0100,9.4170,1.8067,1.9093 | Unigene038894_04 |
| **RNA_profile4-vs-MIR_profile17** | Unigene084995 | novel-m0415-3p | -0.8 | 1.645,0.88,1.435,0.48 | 0.0100,2.3854,4.7341,6.6711 | Unigene084995_01 |
| **RNA_profile5-vs-MIR_profile8** | Unigene056427 | novel-m0164-3p | -0.8 | 1.535,0.405,1.99,1.435 | 3.1421,4.6961,0.0100,9.4037 | Unigene056427_02 |
| **RNA_profile5-vs-MIR_profile8** | Unigene080131 | novel-m0031-5p | -0.8 | 4.65,0.000500,13.09,4.47 | 1.2599,4.6961,0.0100,6.5069 | Unigene080131_02 |
| **RNA_profile5-vs-MIR_profile14** | Unigene018012 | novel-m0245-3p | -0.774596669 | 1.255,0.08,0.91,1.33 | 0.0100,17.6438,0.0100,0.0100 | Unigene018012_01 |
| **RNA_profile5-vs-MIR_profile14** | Unigene092130 | novel-m0434-3p | -0.774596669 | 0.04,0.000500,0.61,0.16 | 0.0100,47.3988,0.0100,0.0100 | Unigene092130_01 |
| **RNA_profile5-vs-MIR_profile14** | Unigene072124 | novel-m0210-3p | -0.774596669 | 11.19,4.67,12.565,8.64 | 0.0100,31.9610,0.0100,0.0100 | Unigene072124_10 |
| **RNA_profile5-vs-MIR_profile14** | Unigene009025 | novel-m0210-3p | -0.774596669 | 4.84,1.895,11.9,5.915 | 0.0100,31.9610,0.0100,0.0100 | Unigene009025_04 |
| **RNA_profile5-vs-MIR_profile14** | Unigene087036 | novel-m0666-3p | -0.774596669 | 4.795,0.000500,10.695,4.62 | 0.0100,796.8899,0.0100,0.0100 | Unigene087036_01 |
| **RNA_profile5-vs-MIR_profile14** | Unigene037819 | novel-m0245-3p | -0.774596669 | 7.75,0.02,1.515,1.69 | 0.0100,17.6438,0.0100,0.0100 | Unigene037819_01 |
| **RNA_profile5-vs-MIR_profile14** | Unigene082178 | novel-m0394-5p | -0.8 | 0.265,0.000500,2.135,1.08 | 3.2225,100.2953,2.8970,6.0707 | Unigene082178_01 |
| **RNA_profile5-vs-MIR_profile14** | Unigene000269 | novel-m0187-3p | -0.8 | 2.53,0.000500,0.7,1.765 | 16.4302,35.4271,20.0668,14.8789 | Unigene000269_01 |
| **RNA_profile5-vs-MIR_profile14** | Unigene088936 | novel-m0394-5p | -0.8 | 1.265,0.605,1.6,1.46 | 3.2225,100.2953,2.8970,6.0707 | Unigene088936_08 |
| **RNA_profile5-vs-MIR_profile14** | Unigene034355 | novel-m0173-5p | -0.774596669 | 5.45,0.000500,3.28,2.815 | 0.0100,37.9469,0.0100,0.0100 | Unigene034355_01 |
| **RNA_profile5-vs-MIR_profile14** | Unigene037281 | novel-m0210-3p | -0.774596669 | 1.59,0.000500,0.33,2.615 | 0.0100,31.9610,0.0100,0.0100 | Unigene037281_01 |
| **RNA_profile5-vs-MIR_profile14** | Unigene069432 | novel-m0245-3p | -0.774596669 | 1.18,0.000500,4.51,2.245 | 0.0100,17.6438,0.0100,0.0100 | Unigene069432_01 |
| **RNA_profile5-vs-MIR_profile14** | Unigene093303 | novel-m0434-3p | -0.774596669 | 3.03,0.455,5.415,2.345 | 0.0100,47.3988,0.0100,0.0100 | Unigene093303_02 |
| **RNA_profile5-vs-MIR_profile14** | Unigene056443 | novel-m0394-5p | -0.8 | 5.57,1.405,3.43,2.62 | 3.2225,100.2953,2.8970,6.0707 | Unigene056443_01 |
| **RNA_profile5-vs-MIR_profile14** | Unigene039634 | novel-m0394-5p | -0.8 | 1.015,0.135,0.975,0.455 | 3.2225,100.2953,2.8970,6.0707 | Unigene039634_02 |
| **RNA_profile5-vs-MIR_profile14** | Unigene024138 | novel-m0531-3p | -0.774596669 | 4.72,0.835,6.82,6.905 | 0.0100,14.1081,0.0100,0.0100 | Unigene024138_01 |
| **RNA_profile5-vs-MIR_profile14** | Unigene071367 | novel-m0639-5p | -0.8 | 12.7,0.035,6.57,9.785 | 5.2676,105.3002,8.2670,2.9361 | Unigene071367_01 |
| **RNA_profile5-vs-MIR_profile14** | Unigene088510 | novel-m0582-3p | -0.774596669 | 0.425,0.000500,0.88,0.51 | 0.0100,40.0833,0.0100,0.0100 | Unigene088510_01 |
| **RNA_profile5-vs-MIR_profile14** | Unigene044787 | novel-m0245-3p | -0.774596669 | 8.055,3.93,11.495,9.09 | 0.0100,17.6438,0.0100,0.0100 | Unigene044787_09 |
| **RNA_profile5-vs-MIR_profile14** | Unigene052415 | novel-m0394-5p | -0.8 | 6.255,3.83,8.6,7.555 | 3.2225,100.2953,2.8970,6.0707 | Unigene052415_04 |
| **RNA_profile5-vs-MIR_profile14** | Unigene019800 | novel-m0187-3p | -0.8 | 1.89,0.19,2.155,2.265 | 16.4302,35.4271,20.0668,14.8789 | Unigene019800_02 |
| **RNA_profile5-vs-MIR_profile14** | Unigene094096 | MIR397-x | -0.774596669 | 2.045,0.000500,1.255,2.155 | 0.0100,7.0715,0.0100,0.0100 | Unigene094096_01 |
| **RNA_profile5-vs-MIR_profile14** | Unigene007200 | novel-m0394-5p | -1 | 13.55,4.85,14.625,13.17 | 3.2225,100.2953,2.8970,6.0707 | Unigene007200_05 |
| **RNA_profile5-vs-MIR_profile14** | Unigene069023 | novel-m0245-3p | -0.774596669 | 1.05,0.285,2.45,0.865 | 0.0100,17.6438,0.0100,0.0100 | Unigene069023_01 |
| **RNA_profile5-vs-MIR_profile14** | Unigene053878 | novel-m0394-5p | -0.8 | 1.06,0.25,1.97,1.095 | 3.2225,100.2953,2.8970,6.0707 | Unigene053878_01 |
| **RNA_profile5-vs-MIR_profile14** | Unigene058308 | novel-m0210-3p | -0.774596669 | 0.415,0.000500,1.72,1.11 | 0.0100,31.9610,0.0100,0.0100 | Unigene058308_01 |
| **RNA_profile5-vs-MIR_profile14** | Unigene039812 | novel-m0666-3p | -0.774596669 | 43.17,14.185,64.48,36.245 | 0.0100,796.8899,0.0100,0.0100 | Unigene039812_03 |
| **RNA_profile5-vs-MIR_profile14** | Unigene087545 | novel-m0666-3p | -0.774596669 | 0.665,0.000500,0.86,0.4 | 0.0100,796.8899,0.0100,0.0100 | Unigene087545_01 |
| **RNA_profile5-vs-MIR_profile14** | Unigene038023 | novel-m0245-3p | -0.774596669 | 3.18,0.915,2.245,2.97 | 0.0100,17.6438,0.0100,0.0100 | Unigene038023_04 |
| **RNA_profile5-vs-MIR_profile14** | Unigene042866 | novel-m0213-5p | -0.8 | 0.91,0.205,2.85,1.485 | 120.2487,253.9055,100.7583,120.3792 | Unigene042866_02 |
| **RNA_profile5-vs-MIR_profile14** | Unigene034383 | novel-m0294-5p | -0.8 | 0.68,0.000500,1.895,0.465 | 1.2599,99.2446,3.4976,6.2691 | Unigene034383_01 |
| **RNA_profile5-vs-MIR_profile14** | Unigene052757 | MIR858-x | -0.8 | 12.105,3.085,10.72,10.985 | 646.9534,2321.2490,678.8600,882.4793 | Unigene052757_08 |
| **RNA_profile5-vs-MIR_profile14** | Unigene018152 | novel-m0245-3p | -0.774596669 | 3.195,0.325,1.18,3.475 | 0.0100,17.6438,0.0100,0.0100 | Unigene018152_02 |
| **RNA_profile5-vs-MIR_profile14** | Unigene067768 | novel-m0294-5p | -0.8 | 146.415,54.365,117.225,125.755 | 1.2599,99.2446,3.4976,6.2691 | Unigene067768_03 |
| **RNA_profile5-vs-MIR_profile14** | Unigene045110 | novel-m0306-5p | -1 | 3.475,0.105,5.24,1.1 | 13.8767,161.8719,4.5273,17.6164 | Unigene045110_01 |
| **RNA_profile5-vs-MIR_profile14** | Unigene075873 | novel-m0666-3p | -0.774596669 | 5.97,4.065,9.83,6.985 | 0.0100,796.8899,0.0100,0.0100 | Unigene075873_02 |
| **RNA_profile5-vs-MIR_profile14** | Unigene039688 | novel-m0288-3p | -1 | 9.095,0.68,7.87,6.885 | 11.9945,102.9197,24.5538,25.2338 | Unigene039688_01 |
| **RNA_profile5-vs-MIR_profile14** | Unigene045160 | novel-m0294-5p | -0.8 | 7.52,0.33,11.745,4.985 | 1.2599,99.2446,3.4976,6.2691 | Unigene045160_05 |
| **RNA_profile5-vs-MIR_profile15** | Unigene047746 | novel-m0247-5p | -0.8 | 16.64,0.000500,16.79,14.185 | 53.1721,229.8227,75.7104,169.0999 | Unigene047746_08 |
| **RNA_profile5-vs-MIR_profile15** | Unigene009390 | novel-m0528-3p | -0.632455532 | 1.2,0.000500,3.645,1.47 | 0.0100,96.8642,0.0100,1.2745 | Unigene009390_01 |
| **RNA_profile5-vs-MIR_profile15** | Unigene067240 | novel-m0361-5p | -0.8 | 1.12,0.000500,0.15,0.17 | 131.6526,464.4361,210.9854,280.8675 | Unigene067240_01 |
| **RNA_profile5-vs-MIR_profile15** | Unigene056443 | novel-m0361-5p | -1 | 5.57,1.405,3.43,2.62 | 131.6526,464.4361,210.9854,280.8675 | Unigene056443_01 |
| **RNA_profile5-vs-MIR_profile15** | Unigene019450 | novel-m0361-5p | -0.8 | 0.81,0.06,0.29,0.395 | 131.6526,464.4361,210.9854,280.8675 | Unigene019450_01 |
| **RNA_profile5-vs-MIR_profile15** | Unigene056955 | novel-m0376-3p | -0.8 | 0.675,0.000500,0.72,0.27 | 27.2641,88.6374,30.4538,80.5043 | Unigene056955_01 |
| **RNA_profile5-vs-MIR_profile15** | Unigene050230 | MIR172-y | -0.6 | 28.83,14.32,25.705,21.705 | 113.3978,184.4507,85.3913,235.8376 | Unigene050230_01 |
| **RNA_profile5-vs-MIR_profile15** | Unigene022856 | novel-m0493-3p | -0.6 | 2.65,0.72,4.39,2.545 | 12.4174,33.0467,14.9798,51.8982 | Unigene022856_01 |
| **RNA_profile5-vs-MIR_profile15** | Unigene006634 | novel-m0528-3p | -0.632455532 | 0.2,0.000500,0.96,0.505 | 0.0100,96.8642,0.0100,1.2745 | Unigene006634_01 |
| **RNA_profile5-vs-MIR_profile15** | Unigene024313 | novel-m0361-5p | -0.8 | 37.84,10.51,22.195,27.365 | 131.6526,464.4361,210.9854,280.8675 | Unigene024313_02 |
| **RNA_profile5-vs-MIR_profile15** | Unigene038023 | novel-m0361-5p | -0.8 | 3.18,0.915,2.245,2.97 | 131.6526,464.4361,210.9854,280.8675 | Unigene038023_04 |
| **RNA_profile5-vs-MIR_profile15** | Unigene040160 | novel-m0528-3p | -0.632455532 | 0.245,0.000500,0.955,0.49 | 0.0100,96.8642,0.0100,1.2745 | Unigene040160_01 |
| **RNA_profile5-vs-MIR_profile15** | Unigene028042 | novel-m0376-3p | -0.8 | 1.855,0.205,0.69,0.8 | 27.2641,88.6374,30.4538,80.5043 | Unigene028042_01 |
| **RNA_profile5-vs-MIR_profile15** | Unigene056311 | novel-m0556-3p | -1 | 2.48,0.000500,2.455,1.04 | 29.8105,106.2812,36.2831,59.6746 | Unigene056311_01 |
| **RNA_profile5-vs-MIR_profile15** | Unigene053755 | novel-m0139-3p | -0.632455532 | 1.085,0.000500,0.105,0.735 | 0.0100,3.5756,0.0100,2.9361 | Unigene053755_01 |
| **RNA_profile5-vs-MIR_profile15** | Unigene067768 | novel-m0528-3p | -0.632455532 | 146.415,54.365,117.225,125.755 | 0.0100,96.8642,0.0100,1.2745 | Unigene067768_03 |
| **RNA_profile5-vs-MIR_profile15** | Unigene034383 | novel-m0528-3p | -0.948683298 | 0.68,0.000500,1.895,0.465 | 0.0100,96.8642,0.0100,1.2745 | Unigene034383_01 |
| **RNA_profile5-vs-MIR_profile15** | Unigene045160 | novel-m0528-3p | -0.948683298 | 7.52,0.33,11.745,4.985 | 0.0100,96.8642,0.0100,1.2745 | Unigene045160_05 |
| **RNA_profile5-vs-MIR_profile15** | Unigene047056 | novel-m0139-3p | -0.948683298 | 8.595,0.025,11.835,7.91 | 0.0100,3.5756,0.0100,2.9361 | Unigene047056_02 |
| **RNA_profile5-vs-MIR_profile15** | Unigene057348 | novel-m0527-5p | -0.8 | 3.55,0.62,1.925,2.18 | 2.8015,27.1306,5.7587,14.0456 | Unigene057348_01 |
| **RNA_profile5-vs-MIR_profile15** | Unigene089804 | novel-m0446-5p | -1 | 3.53,0.000500,0.72,0.365 | 13.6286,96.5852,15.4392,45.7864 | Unigene089804_01 |
| **RNA_profile5-vs-MIR_profile15** | Unigene087760 | novel-m0528-3p | -0.632455532 | 39.135,12.065,50.115,43.945 | 0.0100,96.8642,0.0100,1.2745 | Unigene087760_03 |
| **RNA_profile5-vs-MIR_profile15** | Unigene053752 | novel-m0139-3p | -0.632455532 | 5.685,0.865,3.72,3.825 | 0.0100,3.5756,0.0100,2.9361 | Unigene053752_01 |
| **RNA_profile5-vs-MIR_profile17** | Unigene011730 | novel-m0071-5p | -1 | 1.29,0.000500,1.18,0.855 | 0.0100,35.4271,8.6205,11.1489 | Unigene011730_01 |
| **RNA_profile5-vs-MIR_profile17** | Unigene011730 | novel-m0073-5p | -1 | 1.29,0.000500,1.18,0.855 | 0.0100,35.4271,8.6205,11.1489 | Unigene011730_01 |
| **RNA_profile5-vs-MIR_profile17** | Unigene011730 | novel-m0072-5p | -1 | 1.29,0.000500,1.18,0.855 | 0.0100,35.4271,8.6205,11.1489 | Unigene011730_01 |
| **RNA_profile5-vs-MIR_profile17** | Unigene066825 | novel-m0071-5p | -1 | 3.725,0.76,3.6,2.785 | 0.0100,35.4271,8.6205,11.1489 | Unigene066825_08 |
| **RNA_profile5-vs-MIR_profile17** | Unigene066825 | novel-m0073-5p | -1 | 3.725,0.76,3.6,2.785 | 0.0100,35.4271,8.6205,11.1489 | Unigene066825_08 |
| **RNA_profile5-vs-MIR_profile17** | Unigene066825 | novel-m0072-5p | -1 | 3.725,0.76,3.6,2.785 | 0.0100,35.4271,8.6205,11.1489 | Unigene066825_08 |
| **RNA_profile5-vs-MIR_profile17** | Unigene052487 | novel-m0071-5p | -0.8 | 1.225,0.000500,0.25,0.405 | 0.0100,35.4271,8.6205,11.1489 | Unigene052487_01 |
| **RNA_profile5-vs-MIR_profile17** | Unigene052487 | novel-m0073-5p | -0.8 | 1.225,0.000500,0.25,0.405 | 0.0100,35.4271,8.6205,11.1489 | Unigene052487_01 |
| **RNA_profile5-vs-MIR_profile17** | Unigene052487 | novel-m0072-5p | -0.8 | 1.225,0.000500,0.25,0.405 | 0.0100,35.4271,8.6205,11.1489 | Unigene052487_01 |
| **RNA_profile5-vs-MIR_profile17** | Unigene094425 | novel-m0071-5p | -0.8 | 3.975,2.35,5.515,3.78 | 0.0100,35.4271,8.6205,11.1489 | Unigene094425_02 |
| **RNA_profile5-vs-MIR_profile17** | Unigene094425 | novel-m0073-5p | -0.8 | 3.975,2.35,5.515,3.78 | 0.0100,35.4271,8.6205,11.1489 | Unigene094425_02 |
| **RNA_profile5-vs-MIR_profile17** | Unigene094425 | novel-m0072-5p | -0.8 | 3.975,2.35,5.515,3.78 | 0.0100,35.4271,8.6205,11.1489 | Unigene094425_02 |
| **RNA_profile5-vs-MIR_profile17** | Unigene043342 | novel-m0330-3p | -0.8 | 26.895,3.445,41.325,17.255 | 0.0100,9.4170,1.8067,1.9093 | Unigene043342_10 |
| **RNA_profile5-vs-MIR_profile17** | Unigene050329 | novel-m0071-5p | -0.8 | 1.85,0.000500,4.145,1.305 | 0.0100,35.4271,8.6205,11.1489 | Unigene050329_02 |
| **RNA_profile5-vs-MIR_profile17** | Unigene050329 | novel-m0073-5p | -0.8 | 1.85,0.000500,4.145,1.305 | 0.0100,35.4271,8.6205,11.1489 | Unigene050329_02 |
| **RNA_profile5-vs-MIR_profile17** | Unigene050329 | novel-m0072-5p | -0.8 | 1.85,0.000500,4.145,1.305 | 0.0100,35.4271,8.6205,11.1489 | Unigene050329_02 |
| **RNA_profile5-vs-MIR_profile17** | Unigene091895 | novel-m0071-5p | -0.8 | 2.39,0.000500,0.94,2.33 | 0.0100,35.4271,8.6205,11.1489 | Unigene091895_02 |
| **RNA_profile5-vs-MIR_profile17** | Unigene091895 | novel-m0073-5p | -0.8 | 2.39,0.000500,0.94,2.33 | 0.0100,35.4271,8.6205,11.1489 | Unigene091895_02 |
| **RNA_profile5-vs-MIR_profile17** | Unigene091895 | novel-m0072-5p | -0.8 | 2.39,0.000500,0.94,2.33 | 0.0100,35.4271,8.6205,11.1489 | Unigene091895_02 |
| **RNA_profile5-vs-MIR_profile17** | Unigene007391 | novel-m0071-5p | -1 | 10.05,4.18,7.39,6.325 | 0.0100,35.4271,8.6205,11.1489 | Unigene007391_08 |
| **RNA_profile5-vs-MIR_profile17** | Unigene007391 | novel-m0073-5p | -1 | 10.05,4.18,7.39,6.325 | 0.0100,35.4271,8.6205,11.1489 | Unigene007391_08 |
| **RNA_profile5-vs-MIR_profile17** | Unigene007391 | novel-m0072-5p | -1 | 10.05,4.18,7.39,6.325 | 0.0100,35.4271,8.6205,11.1489 | Unigene007391_08 |
| **RNA_profile5-vs-MIR_profile17** | Unigene050330 | novel-m0071-5p | -0.8 | 10.865,0.000500,15.29,4.3 | 0.0100,35.4271,8.6205,11.1489 | Unigene050330_02 |
| **RNA_profile5-vs-MIR_profile17** | Unigene050330 | novel-m0073-5p | -0.8 | 10.865,0.000500,15.29,4.3 | 0.0100,35.4271,8.6205,11.1489 | Unigene050330_02 |
| **RNA_profile5-vs-MIR_profile17** | Unigene050330 | novel-m0072-5p | -0.8 | 10.865,0.000500,15.29,4.3 | 0.0100,35.4271,8.6205,11.1489 | Unigene050330_02 |
| **RNA_profile5-vs-MIR_profile17** | Unigene052310 | novel-m0360-5p | -0.6 | 0.73,0.000500,0.825,0.59 | 0.0100,2.3854,1.2062,5.6737 | Unigene052310_01 |
| **RNA_profile5-vs-MIR_profile17** | Unigene062011 | novel-m0071-5p | -0.8 | 3.685,0.000500,8.355,0.325 | 0.0100,35.4271,8.6205,11.1489 | Unigene062011_01 |
| **RNA_profile5-vs-MIR_profile17** | Unigene062011 | novel-m0073-5p | -0.8 | 3.685,0.000500,8.355,0.325 | 0.0100,35.4271,8.6205,11.1489 | Unigene062011_01 |
| **RNA_profile5-vs-MIR_profile17** | Unigene062011 | novel-m0072-5p | -0.8 | 3.685,0.000500,8.355,0.325 | 0.0100,35.4271,8.6205,11.1489 | Unigene062011_01 |
| **RNA_profile6-vs-MIR_profile9** | Unigene050738 | novel-m0180-5p | -0.8 | 9.76,5.755,10.99,14.415 | 1.8436,2.3854,2.3317,0.0100 | Unigene050738_02 |
| **RNA_profile6-vs-MIR_profile13** | Unigene052904 | novel-m0574-5p | -1 | 9.685,1.275,5.59,17.055 | 0.9243,68.4737,1.7008,0.0100 | Unigene052904_02 |
| **RNA_profile7-vs-MIR_profile5** | Unigene079960 | novel-m0157-3p | -0.8 | 15.005,19.485,4.62,8.94 | 1.2599,0.0100,3.6085,6.7054 | Unigene079960_03 |
| **RNA_profile7-vs-MIR_profile5** | Unigene087731 | MIR5139-x | -0.8 | 1.77,2.76,0.35,0.63 | 6.7706,0.0100,6.9952,2.9361 | Unigene087731_02 |
| **RNA_profile7-vs-MIR_profile5** | Unigene079546 | MIR5139-x | -0.8 | 20.515,39.26,3.76,6.05 | 6.7706,0.0100,6.9952,2.9361 | Unigene079546_01 |
| **RNA_profile7-vs-MIR_profile5** | Unigene004240 | MIR535-z | -0.632455532 | 0.025,1.125,0.000500,0.000500 | 8.4047,0.0100,9.8569,6.7448 | Unigene004240_01 |
| **RNA_profile7-vs-MIR_profile5** | Unigene044675 | novel-m0307-3p | -0.948683298 | 0.26,1.695,0.000500,0.000500 | 2.6338,0.0100,4.7341,10.2762 | Unigene044675_01 |
| **RNA_profile7-vs-MIR_profile5** | Unigene044675 | novel-m0259-3p | -0.948683298 | 0.26,1.695,0.000500,0.000500 | 2.6338,0.0100,4.7341,10.2762 | Unigene044675_01 |
| **RNA_profile7-vs-MIR_profile5** | Unigene031595 | novel-m0603-3p | -0.948683298 | 0.37,4.235,0.000500,0.000500 | 3.1421,0.0100,3.4976,3.7694 | Unigene031595_01 |
| **RNA_profile7-vs-MIR_profile5** | Unigene026175 | MIR535-z | -0.8 | 4.14,26.94,0.25,1.615 | 8.4047,0.0100,9.8569,6.7448 | Unigene026175_01 |
| **RNA_profile7-vs-MIR_profile5** | Unigene034994 | novel-m0618-5p | -0.8 | 0.585,1.165,0.19,0.39 | 5.1435,0.0100,5.2993,1.6715 | Unigene034994_01 |
| **RNA_profile7-vs-MIR_profile5** | Unigene010208 | novel-m0313-5p | -0.6 | 1.43,1.245,0.565,0.84 | 2.1742,0.0100,2.4074,7.3402 | Unigene010208_01 |
| **RNA_profile7-vs-MIR_profile5** | Unigene033892 | MIR5139-x | -0.8 | 6.345,6.81,3.36,4.18 | 6.7706,0.0100,6.9952,2.9361 | Unigene033892_09 |
| **RNA_profile7-vs-MIR_profile5** | Unigene084102 | MIR9726-y | -1 | 1.4,1.41,0.645,0.65 | 0.9243,0.0100,2.8970,1.2745 | Unigene084102_01 |
| **RNA_profile7-vs-MIR_profile5** | Unigene095700 | MIR8175-y | -0.6 | 10.46,5.135,1.37,1.87 | 3.5531,0.0100,5.0926,6.7054 | Unigene095700_03 |
| **RNA_profile7-vs-MIR_profile5** | Unigene075410 | novel-m0533-5p | -1 | 2.15,2.155,0.55,0.78 | 42.3083,0.0100,122.5579,97.7220 | Unigene075410_01 |
| **RNA_profile7-vs-MIR_profile5** | Unigene006609 | novel-m0606-3p | -1 | 66.29,117.085,13.38,36.45 | 76.0144,30.6663,118.3522,111.8482 | Unigene006609_01 |
| **RNA_profile7-vs-MIR_profile5** | Unigene040273 | MIR8175-y | -0.8 | 12.22,15.005,4.565,7.65 | 3.5531,0.0100,5.0926,6.7054 | Unigene040273_06 |
| **RNA_profile7-vs-MIR_profile5** | Unigene047925 | novel-m0335-5p | -0.8 | 3.575,3.935,1.925,2.135 | 3.0935,0.0100,7.6311,9.4430 | Unigene047925_01 |
| **RNA_profile7-vs-MIR_profile5** | Unigene080010 | novel-m0316-5p | -0.8 | 0.9,1.335,0.155,0.28 | 0.9243,0.0100,2.8315,3.5709 | Unigene080010_04 |
| **RNA_profile7-vs-MIR_profile5** | Unigene080010 | novel-m0644-5p | -0.8 | 0.9,1.335,0.155,0.28 | 0.9243,0.0100,2.8315,3.5709 | Unigene080010_04 |
| **RNA_profile7-vs-MIR_profile5** | Unigene075041 | novel-m0041-3p | -0.8 | 3.315,10.535,0.89,1.44 | 11.3740,0.0100,12.7893,33.2499 | Unigene075041_02 |
| **RNA_profile7-vs-MIR_profile5** | Unigene045205 | novel-m0412-5p | -1 | 2.11,3.68,0.94,1.25 | 138.3795,40.1530,413.5979,327.0849 | Unigene045205_02 |
| **RNA_profile7-vs-MIR_profile5** | Unigene095205 | MIR9726-y | -0.6 | 0.465,0.395,0.16,0.115 | 0.9243,0.0100,2.8970,1.2745 | Unigene095205_01 |
| **RNA_profile7-vs-MIR_profile5** | Unigene004120 | MIR8762-x | -0.632455532 | 0.38,0.68,0.000500,0.000500 | 2.8015,0.0100,2.3317,3.5709 | Unigene004120_01 |
| **RNA_profile7-vs-MIR_profile5** | Unigene070303 | novel-m0442-3p | -0.8 | 4.325,5.93,2.125,2.255 | 10.4111,0.0100,11.6587,44.4381 | Unigene070303_01 |
| **RNA_profile7-vs-MIR_profile5** | Unigene056441 | novel-m0593-5p | -0.8 | 1.62,1.06,0.195,0.11 | 0.9243,0.0100,1.2062,4.6026 | Unigene056441_02 |
| **RNA_profile7-vs-MIR_profile5** | Unigene056441 | novel-m0027-5p | -0.8 | 1.62,1.06,0.195,0.11 | 0.9243,0.0100,1.2062,4.6026 | Unigene056441_02 |
| **RNA_profile7-vs-MIR_profile5** | Unigene056441 | novel-m0395-5p | -0.8 | 1.62,1.06,0.195,0.11 | 0.9243,0.0100,1.2062,4.6026 | Unigene056441_02 |
| **RNA_profile7-vs-MIR_profile5** | Unigene056441 | novel-m0123-5p | -0.8 | 1.62,1.06,0.195,0.11 | 0.9243,0.0100,1.2062,4.6026 | Unigene056441_02 |
| **RNA_profile7-vs-MIR_profile5** | Unigene085936 | novel-m0593-5p | -0.6 | 29.325,26.66,13.85,18.5 | 0.9243,0.0100,1.2062,4.6026 | Unigene085936_01 |
| **RNA_profile7-vs-MIR_profile5** | Unigene085936 | novel-m0027-5p | -0.6 | 29.325,26.66,13.85,18.5 | 0.9243,0.0100,1.2062,4.6026 | Unigene085936_01 |
| **RNA_profile7-vs-MIR_profile5** | Unigene085936 | novel-m0395-5p | -0.6 | 29.325,26.66,13.85,18.5 | 0.9243,0.0100,1.2062,4.6026 | Unigene085936_01 |
| **RNA_profile7-vs-MIR_profile5** | Unigene085936 | novel-m0123-5p | -0.6 | 29.325,26.66,13.85,18.5 | 0.9243,0.0100,1.2062,4.6026 | Unigene085936_01 |
| **RNA_profile7-vs-MIR_profile5** | Unigene015827 | MIR535-z | -0.8 | 1.985,3.52,0.46,1.08 | 8.4047,0.0100,9.8569,6.7448 | Unigene015827_01 |
| **RNA_profile7-vs-MIR_profile6** | Unigene087731 | novel-m0444-3p | -0.8 | 1.77,2.76,0.35,0.63 | 25.6300,7.1462,44.6565,71.3385 | Unigene087731_02 |
| **RNA_profile7-vs-MIR_profile12** | Unigene047724 | novel-m0432-3p | -0.737864787 | 0.735,0.655,0.16,0.335 | 0.0100,0.0100,5.8293,6.3085 | Unigene047724_01 |
| **RNA_profile7-vs-MIR_profile12** | Unigene047724 | novel-m0623-3p | -0.737864787 | 0.735,0.655,0.16,0.335 | 0.0100,0.0100,5.8293,6.3085 | Unigene047724_01 |
| **RNA_profile7-vs-MIR_profile12** | Unigene047724 | novel-m0323-3p | -0.737864787 | 0.735,0.655,0.16,0.335 | 0.0100,0.0100,5.8293,6.3085 | Unigene047724_01 |
| **RNA_profile7-vs-MIR_profile12** | Unigene047724 | novel-m0492-5p | -0.737864787 | 0.735,0.655,0.16,0.335 | 0.0100,0.0100,5.8293,6.3085 | Unigene047724_01 |
| **RNA_profile7-vs-MIR_profile12** | Unigene052735 | MIR5054-y | -0.737864787 | 4.08,3.55,1.37,2.045 | 0.0100,0.0100,1.2062,3.7694 | Unigene052735_01 |
| **RNA_profile7-vs-MIR_profile12** | Unigene028173 | novel-m0628-3p | -0.737864787 | 2.72,3.325,1.195,1.435 | 0.0100,0.0100,2.3317,8.4113 | Unigene028173_03 |
| **RNA_profile7-vs-MIR_profile12** | Unigene028173 | novel-m0626-3p | -0.737864787 | 2.72,3.325,1.195,1.435 | 0.0100,0.0100,2.3317,8.4113 | Unigene028173_03 |
| **RNA_profile7-vs-MIR_profile12** | Unigene026351 | novel-m0263-3p | -0.888888889 | 0.085,1.715,0.000500,0.000500 | 0.0100,0.0100,4.1335,3.5709 | Unigene026351_01 |
| **RNA_profile7-vs-MIR_profile12** | Unigene008554 | novel-m0432-3p | -0.737864787 | 1.42,0.995,0.235,0.32 | 0.0100,0.0100,5.8293,6.3085 | Unigene008554_03 |
| **RNA_profile7-vs-MIR_profile12** | Unigene008554 | novel-m0623-3p | -0.737864787 | 1.42,0.995,0.235,0.32 | 0.0100,0.0100,5.8293,6.3085 | Unigene008554_03 |
| **RNA_profile7-vs-MIR_profile12** | Unigene008554 | novel-m0323-3p | -0.737864787 | 1.42,0.995,0.235,0.32 | 0.0100,0.0100,5.8293,6.3085 | Unigene008554_03 |
| **RNA_profile7-vs-MIR_profile12** | Unigene008554 | novel-m0492-5p | -0.737864787 | 1.42,0.995,0.235,0.32 | 0.0100,0.0100,5.8293,6.3085 | Unigene008554_03 |
| **RNA_profile8-vs-MIR_profile5** | Unigene012732 | novel-m0134-3p | -0.8 | 0.475,0.975,0.275,0.565 | 6.3984,0.0100,2.3317,1.2745 | Unigene012732_01 |
| **RNA_profile8-vs-MIR_profile5** | Unigene012732 | novel-m0374-5p | -0.8 | 0.475,0.975,0.275,0.565 | 2.6338,0.0100,2.4074,1.2745 | Unigene012732_01 |
| **RNA_profile8-vs-MIR_profile5** | Unigene038043 | MIR8565-x | -0.8 | 190.305,224.525,86.685,176.145 | 60.5771,0.0100,69.8093,24.1628 | Unigene038043_04 |
| **RNA_profile8-vs-MIR_profile5** | Unigene076680 | MIR5139-x | -0.8 | 6.555,8.185,1.895,4.6 | 6.7706,0.0100,6.9952,2.9361 | Unigene076680_04 |
| **RNA_profile8-vs-MIR_profile5** | Unigene075024 | novel-m0316-5p | -0.8 | 1.56,6.55,0.235,1.15 | 0.9243,0.0100,2.8315,3.5709 | Unigene075024_02 |
| **RNA_profile8-vs-MIR_profile5** | Unigene075024 | novel-m0644-5p | -0.8 | 1.56,6.55,0.235,1.15 | 0.9243,0.0100,2.8315,3.5709 | Unigene075024_02 |
| **RNA_profile8-vs-MIR_profile5** | Unigene075573 | novel-m0039-3p | -0.8 | 0.885,2.135,0.25,0.685 | 2.6338,0.0100,4.0629,16.9817 | Unigene075573_04 |
| **RNA_profile8-vs-MIR_profile5** | Unigene030535 | novel-m0371-3p | -0.8 | 16.645,24.055,10.935,16.695 | 6.3546,0.0100,3.0079,2.5441 | Unigene030535_02 |
| **RNA_profile8-vs-MIR_profile5** | Unigene030535 | novel-m0102-3p | -1 | 16.645,24.055,10.935,16.695 | 10.2433,0.0100,13.6016,10.0778 | Unigene030535_02 |
| **RNA_profile8-vs-MIR_profile5** | Unigene022173 | novel-m0335-5p | -0.8 | 1.085,2.11,0.12,0.72 | 3.0935,0.0100,7.6311,9.4430 | Unigene022173_01 |
| **RNA_profile8-vs-MIR_profile5** | Unigene023388 | novel-m0134-3p | -0.8 | 0.6,2,0.115,0.76 | 6.3984,0.0100,2.3317,1.2745 | Unigene023388_02 |
| **RNA_profile8-vs-MIR_profile5** | Unigene095200 | MIR535-z | -0.8 | 0.6,0.905,0.05,0.475 | 8.4047,0.0100,9.8569,6.7448 | Unigene095200_01 |
| **RNA_profile8-vs-MIR_profile5** | Unigene094959 | MIR8155-y | -0.6 | 0.12,3.34,0.000500,4.95 | 4.9320,0.0100,3.4623,2.9361 | Unigene094959_01 |
| **RNA_profile8-vs-MIR_profile5** | Unigene094959 | MIR845-z | -0.6 | 0.12,3.34,0.000500,4.95 | 337.6773,52.0549,234.1641,191.9574 | Unigene094959_01 |
| **RNA_profile8-vs-MIR_profile5** | Unigene094959 | MIR5139-x | -0.8 | 0.12,3.34,0.000500,4.95 | 6.7706,0.0100,6.9952,2.9361 | Unigene094959_01 |
| **RNA_profile8-vs-MIR_profile5** | Unigene036331 | novel-m0039-3p | -0.6 | 6.95,4.31,1,4.16 | 2.6338,0.0100,4.0629,16.9817 | Unigene036331_03 |
| **RNA_profile8-vs-MIR_profile5** | Unigene036331 | novel-m0027-5p | -0.6 | 6.95,4.31,1,4.16 | 0.9243,0.0100,1.2062,4.6026 | Unigene036331_03 |
| **RNA_profile8-vs-MIR_profile5** | Unigene036331 | novel-m0123-5p | -0.6 | 6.95,4.31,1,4.16 | 0.9243,0.0100,1.2062,4.6026 | Unigene036331_03 |
| **RNA_profile8-vs-MIR_profile5** | Unigene036331 | novel-m0259-3p | -0.6 | 6.95,4.31,1,4.16 | 2.6338,0.0100,4.7341,10.2762 | Unigene036331_03 |
| **RNA_profile8-vs-MIR_profile5** | Unigene036331 | novel-m0395-5p | -0.6 | 6.95,4.31,1,4.16 | 0.9243,0.0100,1.2062,4.6026 | Unigene036331_03 |
| **RNA_profile8-vs-MIR_profile5** | Unigene036331 | novel-m0307-3p | -0.6 | 6.95,4.31,1,4.16 | 2.6338,0.0100,4.7341,10.2762 | Unigene036331_03 |
| **RNA_profile8-vs-MIR_profile5** | Unigene036331 | novel-m0593-5p | -0.6 | 6.95,4.31,1,4.16 | 0.9243,0.0100,1.2062,4.6026 | Unigene036331_03 |
| **RNA_profile8-vs-MIR_profile5** | Unigene074956 | MIR535-z | -0.8 | 8.435,10.61,5.28,7.78 | 8.4047,0.0100,9.8569,6.7448 | Unigene074956_05 |
| **RNA_profile8-vs-MIR_profile5** | Unigene085471 | MIR5139-x | -0.8 | 2.01,2.34,0.715,1.505 | 6.7706,0.0100,6.9952,2.9361 | Unigene085471_02 |
| **RNA_profile8-vs-MIR_profile5** | Unigene095967 | novel-m0259-3p | -0.6 | 10.11,9.65,4.785,9.39 | 2.6338,0.0100,4.7341,10.2762 | Unigene095967_01 |
| **RNA_profile8-vs-MIR_profile5** | Unigene095967 | novel-m0307-3p | -0.6 | 10.11,9.65,4.785,9.39 | 2.6338,0.0100,4.7341,10.2762 | Unigene095967_01 |
| **RNA_profile8-vs-MIR_profile5** | Unigene052164 | novel-m0313-5p | -0.8 | 1.165,1.28,0.185,0.975 | 2.1742,0.0100,2.4074,7.3402 | Unigene052164_01 |
| **RNA_profile8-vs-MIR_profile5** | Unigene004565 | MIR9726-y | -0.8 | 2.555,2.165,1.19,2.145 | 0.9243,0.0100,2.8970,1.2745 | Unigene004565_01 |
| **RNA_profile8-vs-MIR_profile5** | Unigene064840 | MIR9726-y | -1 | 0.435,20.29,0.000500,0.21 | 0.9243,0.0100,2.8970,1.2745 | Unigene064840_02 |
| **RNA_profile8-vs-MIR_profile5** | Unigene046807 | novel-m0027-5p | -0.8 | 37.265,54.39,5.385,28.95 | 0.9243,0.0100,1.2062,4.6026 | Unigene046807_03 |
| **RNA_profile8-vs-MIR_profile5** | Unigene046807 | novel-m0123-5p | -0.8 | 37.265,54.39,5.385,28.95 | 0.9243,0.0100,1.2062,4.6026 | Unigene046807_03 |
| **RNA_profile8-vs-MIR_profile5** | Unigene046807 | novel-m0395-5p | -0.8 | 37.265,54.39,5.385,28.95 | 0.9243,0.0100,1.2062,4.6026 | Unigene046807_03 |
| **RNA_profile8-vs-MIR_profile5** | Unigene046807 | novel-m0593-5p | -0.8 | 37.265,54.39,5.385,28.95 | 0.9243,0.0100,1.2062,4.6026 | Unigene046807_03 |
| **RNA_profile8-vs-MIR_profile5** | Unigene095803 | MIR9726-y | -0.8 | 0.365,1.945,0.000500,0.39 | 0.9243,0.0100,2.8970,1.2745 | Unigene095803_01 |
| **RNA_profile8-vs-MIR_profile5** | Unigene061236 | MIR5139-x | -0.8 | 36.225,48.225,20.18,32.81 | 6.7706,0.0100,6.9952,2.9361 | Unigene061236_06 |
| **RNA_profile8-vs-MIR_profile5** | Unigene059563 | novel-m0374-5p | -0.8 | 1.09,1.975,0.325,1.31 | 2.6338,0.0100,2.4074,1.2745 | Unigene059563_01 |
| **RNA_profile8-vs-MIR_profile5** | Unigene059563 | novel-m0070-5p | -1 | 1.09,1.975,0.325,1.31 | 1.3839,0.0100,4.7341,1.2745 | Unigene059563_01 |
| **RNA_profile8-vs-MIR_profile5** | Unigene090071 | novel-m0027-5p | -0.6 | 5.265,4.04,1.185,3.96 | 0.9243,0.0100,1.2062,4.6026 | Unigene090071_02 |
| **RNA_profile8-vs-MIR_profile5** | Unigene090071 | novel-m0123-5p | -0.6 | 5.265,4.04,1.185,3.96 | 0.9243,0.0100,1.2062,4.6026 | Unigene090071_02 |
| **RNA_profile8-vs-MIR_profile5** | Unigene090071 | novel-m0395-5p | -0.6 | 5.265,4.04,1.185,3.96 | 0.9243,0.0100,1.2062,4.6026 | Unigene090071_02 |
| **RNA_profile8-vs-MIR_profile5** | Unigene090071 | novel-m0593-5p | -0.6 | 5.265,4.04,1.185,3.96 | 0.9243,0.0100,1.2062,4.6026 | Unigene090071_02 |
| **RNA_profile8-vs-MIR_profile5** | Unigene039772 | MIR8175-y | -0.8 | 9.9,14.605,4.245,8.325 | 3.5531,0.0100,5.0926,6.7054 | Unigene039772_01 |
| **RNA_profile8-vs-MIR_profile5** | Unigene062816 | MIR535-z | -0.8 | 104.715,113.56,59.845,121.48 | 8.4047,0.0100,9.8569,6.7448 | Unigene062816_05 |
| **RNA_profile8-vs-MIR_profile5** | Unigene006658 | MIR535-z | -1 | 1.565,2.49,0.825,1.77 | 8.4047,0.0100,9.8569,6.7448 | Unigene006658_01 |
| **RNA_profile8-vs-MIR_profile5** | Unigene093879 | novel-m0512-5p | -0.8 | 0.41,0.66,0.16,0.48 | 337.6627,0.0100,452.4956,369.1502 | Unigene093879_01 |
| **RNA_profile8-vs-MIR_profile5** | Unigene022073 | MIR5139-x | -0.8 | 6.59,19.125,0.000500,2.355 | 6.7706,0.0100,6.9952,2.9361 | Unigene022073_01 |
| **RNA_profile8-vs-MIR_profile5** | Unigene035384 | novel-m0134-3p | -0.8 | 5.545,5.92,1.17,5.905 | 6.3984,0.0100,2.3317,1.2745 | Unigene035384_01 |
| **RNA_profile8-vs-MIR_profile5** | Unigene035384 | novel-m0374-5p | -0.8 | 5.545,5.92,1.17,5.905 | 2.6338,0.0100,2.4074,1.2745 | Unigene035384_01 |
| **RNA_profile8-vs-MIR_profile5** | Unigene076174 | MIR535-z | -0.8 | 59.265,75.825,27.415,57.995 | 8.4047,0.0100,9.8569,6.7448 | Unigene076174_01 |
| **RNA_profile8-vs-MIR_profile5** | Unigene043100 | MIR9726-y | -1 | 2.645,5.94,0.375,2.045 | 0.9243,0.0100,2.8970,1.2745 | Unigene043100_01 |
| **RNA_profile8-vs-MIR_profile5** | Unigene004313 | MIR5139-x | -0.8 | 0.605,1.28,0.12,0.54 | 6.7706,0.0100,6.9952,2.9361 | Unigene004313_01 |
| **RNA_profile8-vs-MIR_profile5** | Unigene065141 | novel-m0566-3p | -0.8 | 20.31,62.95,5.995,26.11 | 16.2187,0.0100,12.7893,6.9039 | Unigene065141_01 |
| **RNA_profile8-vs-MIR_profile5** | Unigene046116 | novel-m0409-3p | -0.8 | 16.945,48.395,3.325,11.215 | 75.6352,2.3854,129.7306,230.7593 | Unigene046116_02 |
| **RNA_profile8-vs-MIR_profile5** | Unigene046116 | novel-m0410-3p | -0.8 | 16.945,48.395,3.325,11.215 | 75.6352,2.3854,129.7306,230.7593 | Unigene046116_02 |
| **RNA_profile8-vs-MIR_profile5** | Unigene046116 | novel-m0329-3p | -0.8 | 16.945,48.395,3.325,11.215 | 10.1191,0.0100,16.5345,31.5834 | Unigene046116_02 |
| **RNA_profile8-vs-MIR_profile5** | Unigene046116 | novel-m0471-3p | -0.8 | 16.945,48.395,3.325,11.215 | 75.6352,2.3854,129.7306,230.7593 | Unigene046116_02 |
| **RNA_profile8-vs-MIR_profile5** | Unigene001395 | MIR535-z | -0.8 | 0.915,1.71,0.25,0.685 | 8.4047,0.0100,9.8569,6.7448 | Unigene001395_01 |
| **RNA_profile8-vs-MIR_profile5** | Unigene023908 | novel-m0316-5p | -0.6 | 0.885,0.58,0.000500,0.45 | 0.9243,0.0100,2.8315,3.5709 | Unigene023908_01 |
| **RNA_profile8-vs-MIR_profile5** | Unigene023908 | novel-m0644-5p | -0.6 | 0.885,0.58,0.000500,0.45 | 0.9243,0.0100,2.8315,3.5709 | Unigene023908_01 |
| **RNA_profile8-vs-MIR_profile5** | Unigene059751 | novel-m0041-3p | -0.8 | 2.375,2.575,0.145,1.68 | 11.3740,0.0100,12.7893,33.2499 | Unigene059751_04 |
| **RNA_profile8-vs-MIR_profile5** | Unigene025737 | novel-m0520-3p | -1 | 0.65,3.29,0.000500,0.275 | 2.8015,0.0100,22.1868,13.2910 | Unigene025737_01 |
| **RNA_profile8-vs-MIR_profile5** | Unigene070560 | MIR535-z | -1 | 30.26,49.66,15.625,38.43 | 8.4047,0.0100,9.8569,6.7448 | Unigene070560_06 |
| **RNA_profile8-vs-MIR_profile5** | Unigene015574 | MIR535-z | -1 | 0.63,1.07,0.395,0.78 | 8.4047,0.0100,9.8569,6.7448 | Unigene015574_01 |
| **RNA_profile8-vs-MIR_profile5** | Unigene075083 | MIR535-z | -1 | 1.035,1.255,0.475,1.04 | 8.4047,0.0100,9.8569,6.7448 | Unigene075083_02 |
| **RNA_profile8-vs-MIR_profile5** | Unigene030359 | novel-m0041-3p | -0.6 | 1.185,1.18,0.43,0.96 | 11.3740,0.0100,12.7893,33.2499 | Unigene030359_01 |
| **RNA_profile8-vs-MIR_profile5** | Unigene094214 | novel-m0039-3p | -0.8 | 189.665,252.82,60.58,159.565 | 2.6338,0.0100,4.0629,16.9817 | Unigene094214_12 |
| **RNA_profile8-vs-MIR_profile5** | Unigene040204 | novel-m0575-5p | -0.8 | 25.905,30.505,11.45,18.975 | 14.1755,0.0100,29.7120,76.1002 | Unigene040204_05 |
| **RNA_profile8-vs-MIR_profile5** | Unigene091034 | novel-m0412-5p | -0.8 | 0.2,0.535,0.065,0.505 | 138.3795,40.1530,413.5979,327.0849 | Unigene091034_01 |
| **RNA_profile8-vs-MIR_profile5** | Unigene070743 | MIR5139-x | -0.8 | 0.245,1.45,0.000500,0.1 | 6.7706,0.0100,6.9952,2.9361 | Unigene070743_03 |
| **RNA_profile8-vs-MIR_profile5** | Unigene003987 | novel-m0039-3p | -0.8 | 1.285,1.43,0.000500,0.735 | 2.6338,0.0100,4.0629,16.9817 | Unigene003987_01 |
| **RNA_profile8-vs-MIR_profile5** | Unigene003987 | novel-m0027-5p | -0.8 | 1.285,1.43,0.000500,0.735 | 0.9243,0.0100,1.2062,4.6026 | Unigene003987_01 |
| **RNA_profile8-vs-MIR_profile5** | Unigene003987 | novel-m0123-5p | -0.8 | 1.285,1.43,0.000500,0.735 | 0.9243,0.0100,1.2062,4.6026 | Unigene003987_01 |
| **RNA_profile8-vs-MIR_profile5** | Unigene003987 | novel-m0395-5p | -0.8 | 1.285,1.43,0.000500,0.735 | 0.9243,0.0100,1.2062,4.6026 | Unigene003987_01 |
| **RNA_profile8-vs-MIR_profile5** | Unigene003987 | novel-m0593-5p | -0.8 | 1.285,1.43,0.000500,0.735 | 0.9243,0.0100,1.2062,4.6026 | Unigene003987_01 |
| **RNA_profile8-vs-MIR_profile5** | Unigene071528 | MIR535-z | -0.8 | 66.215,95.415,29.82,65.105 | 8.4047,0.0100,9.8569,6.7448 | Unigene071528_01 |
| **RNA_profile8-vs-MIR_profile5** | Unigene072929 | MIR8175-y | -0.6 | 9.865,8.48,1.765,4.765 | 3.5531,0.0100,5.0926,6.7054 | Unigene072929_01 |
| **RNA_profile8-vs-MIR_profile5** | Unigene081575 | novel-m0594-5p | -0.8 | 25152.12,40045.275,12686.58,29429.765 | 1.8872,0.0100,12.2592,5.6737 | Unigene081575_03 |
| **RNA_profile8-vs-MIR_profile6** | Unigene004313 | novel-m0445-3p | -0.8 | 0.605,1.28,0.12,0.54 | 25.6300,7.1462,44.6565,71.3385 | Unigene004313_01 |
| **RNA_profile8-vs-MIR_profile6** | Unigene004313 | novel-m0444-3p | -0.8 | 0.605,1.28,0.12,0.54 | 25.6300,7.1462,44.6565,71.3385 | Unigene004313_01 |
| **RNA_profile8-vs-MIR_profile6** | Unigene064483 | novel-m0445-3p | -0.8 | 2.22,9.225,0.31,1.95 | 25.6300,7.1462,44.6565,71.3385 | Unigene064483_01 |
| **RNA_profile8-vs-MIR_profile6** | Unigene064483 | novel-m0444-3p | -0.8 | 2.22,9.225,0.31,1.95 | 25.6300,7.1462,44.6565,71.3385 | Unigene064483_01 |
| **RNA_profile8-vs-MIR_profile12** | Unigene003987 | novel-m0628-3p | -0.737864787 | 1.285,1.43,0.000500,0.735 | 0.0100,0.0100,2.3317,8.4113 | Unigene003987_01 |
| **RNA_profile8-vs-MIR_profile12** | Unigene003987 | novel-m0626-3p | -0.737864787 | 1.285,1.43,0.000500,0.735 | 0.0100,0.0100,2.3317,8.4113 | Unigene003987_01 |
| **RNA_profile8-vs-MIR_profile12** | Unigene084319 | novel-m0432-3p | -0.737864787 | 0.07,1.94,0.000500,0.06 | 0.0100,0.0100,5.8293,6.3085 | Unigene084319_01 |
| **RNA_profile8-vs-MIR_profile12** | Unigene084319 | novel-m0623-3p | -0.737864787 | 0.07,1.94,0.000500,0.06 | 0.0100,0.0100,5.8293,6.3085 | Unigene084319_01 |
| **RNA_profile8-vs-MIR_profile12** | Unigene084319 | novel-m0323-3p | -0.737864787 | 0.07,1.94,0.000500,0.06 | 0.0100,0.0100,5.8293,6.3085 | Unigene084319_01 |
| **RNA_profile8-vs-MIR_profile12** | Unigene084319 | novel-m0492-5p | -0.737864787 | 0.07,1.94,0.000500,0.06 | 0.0100,0.0100,5.8293,6.3085 | Unigene084319_01 |
| **RNA_profile8-vs-MIR_profile12** | Unigene004313 | MIR845-x | -0.8 | 0.605,1.28,0.12,0.54 | 99445.7811,72438.8438,167614.6491,169189.4274 | Unigene004313_01 |
| **RNA_profile8-vs-MIR_profile12** | Unigene051825 | novel-m0628-3p | -0.737864787 | 0.555,0.515,0.045,0.34 | 0.0100,0.0100,2.3317,8.4113 | Unigene051825_01 |
| **RNA_profile8-vs-MIR_profile12** | Unigene051825 | novel-m0626-3p | -0.737864787 | 0.555,0.515,0.045,0.34 | 0.0100,0.0100,2.3317,8.4113 | Unigene051825_01 |
| **RNA_profile8-vs-MIR_profile12** | Unigene048279 | novel-m0432-3p | -0.737864787 | 8.91,12.675,5.37,8.53 | 0.0100,0.0100,5.8293,6.3085 | Unigene048279_03 |
| **RNA_profile8-vs-MIR_profile12** | Unigene048279 | novel-m0623-3p | -0.737864787 | 8.91,12.675,5.37,8.53 | 0.0100,0.0100,5.8293,6.3085 | Unigene048279_03 |
| **RNA_profile8-vs-MIR_profile12** | Unigene048279 | novel-m0323-3p | -0.737864787 | 8.91,12.675,5.37,8.53 | 0.0100,0.0100,5.8293,6.3085 | Unigene048279_03 |
| **RNA_profile8-vs-MIR_profile12** | Unigene048279 | novel-m0492-5p | -0.737864787 | 8.91,12.675,5.37,8.53 | 0.0100,0.0100,5.8293,6.3085 | Unigene048279_03 |
| **RNA_profile8-vs-MIR_profile12** | Unigene090071 | novel-m0628-3p | -0.737864787 | 5.265,4.04,1.185,3.96 | 0.0100,0.0100,2.3317,8.4113 | Unigene090071_02 |
| **RNA_profile8-vs-MIR_profile12** | Unigene090071 | novel-m0626-3p | -0.737864787 | 5.265,4.04,1.185,3.96 | 0.0100,0.0100,2.3317,8.4113 | Unigene090071_02 |
| **RNA_profile8-vs-MIR_profile12** | Unigene064483 | MIR845-x | -0.8 | 2.22,9.225,0.31,1.95 | 99445.7811,72438.8438,167614.6491,169189.4274 | Unigene064483_01 |
| **RNA_profile8-vs-MIR_profile12** | Unigene008555 | novel-m0432-3p | -0.737864787 | 4.43,2.28,0.115,1.315 | 0.0100,0.0100,5.8293,6.3085 | Unigene008555_03 |
| **RNA_profile8-vs-MIR_profile12** | Unigene008555 | novel-m0623-3p | -0.737864787 | 4.43,2.28,0.115,1.315 | 0.0100,0.0100,5.8293,6.3085 | Unigene008555_03 |
| **RNA_profile8-vs-MIR_profile12** | Unigene008555 | novel-m0323-3p | -0.737864787 | 4.43,2.28,0.115,1.315 | 0.0100,0.0100,5.8293,6.3085 | Unigene008555_03 |
| **RNA_profile8-vs-MIR_profile12** | Unigene008555 | novel-m0492-5p | -0.737864787 | 4.43,2.28,0.115,1.315 | 0.0100,0.0100,5.8293,6.3085 | Unigene008555_03 |
| **RNA_profile9-vs-MIR_profile3** | Unigene029523 | MIR1134-z | -0.737864787 | 0.125,0.645,1.085,0.000500 | 2.8015,0.0100,0.0100,1.6715 | Unigene029523_01 |
| **RNA_profile9-vs-MIR_profile3** | Unigene067106 | MIR1134-z | -0.737864787 | 0.175,0.245,0.805,0.035 | 2.8015,0.0100,0.0100,1.6715 | Unigene067106_01 |
| **RNA_profile9-vs-MIR_profile3** | Unigene064792 | MIR1134-z | -0.737864787 | 0.155,0.335,1.03,0.000500 | 2.8015,0.0100,0.0100,1.6715 | Unigene064792_01 |
| **RNA_profile9-vs-MIR_profile3** | Unigene022932 | MIR1134-z | -0.737864787 | 4.115,4.96,4.23,1.65 | 2.8015,0.0100,0.0100,1.6715 | Unigene022932_01 |
| **RNA_profile10-vs-MIR_profile16** | Unigene007262 | MIR7783-y | -0.737864787 | 0.025,0.005,0.015,95.295 | 0.0100,2.3854,2.8970,0.0100 | Unigene007262_02 |
| **RNA_profile11-vs-MIR_profile7** | Unigene041624 | novel-m0171-5p | -0.948683298 | 45.185,28,119.345,61.325 | 1.3839,11.7626,0.0100,0.0100 | Unigene041624_09 |
| **RNA_profile11-vs-MIR_profile7** | Unigene041624 | novel-m0170-5p | -0.948683298 | 45.185,28,119.345,61.325 | 1.3839,11.7626,0.0100,0.0100 | Unigene041624_09 |
| **RNA_profile11-vs-MIR_profile13** | Unigene011215 | novel-m0015-3p | -0.8 | 21.58,16,35.045,22.17 | 5.2676,25.9752,4.7341,1.2745 | Unigene011215_01 |
| **RNA_profile11-vs-MIR_profile15** | Unigene081666 | novel-m0595-3p | -0.6 | 0.56,0.145,2.225,0.5 | 18.4802,65.0077,36.2126,99.4690 | Unigene081666_01 |
| **RNA_profile11-vs-MIR_profile15** | Unigene088913 | novel-m0595-3p | -0.6 | 3.43,1.8,8.005,2.695 | 18.4802,65.0077,36.2126,99.4690 | Unigene088913_01 |
| **RNA_profile11-vs-MIR_profile15** | Unigene074767 | novel-m0595-3p | -0.8 | 1.245,0.915,2.405,0.695 | 18.4802,65.0077,36.2126,99.4690 | Unigene074767_01 |
| **RNA_profile11-vs-MIR_profile15** | Unigene094411 | novel-m0370-5p | -0.948683298 | 5.92,3.95,9.1,5.395 | 0.0100,7.0715,0.0100,1.2745 | Unigene094411_03 |
| **RNA_profile11-vs-MIR_profile15** | Unigene034852 | novel-m0528-3p | -0.632455532 | 16.55,9.585,40.215,19.97 | 0.0100,96.8642,0.0100,1.2745 | Unigene034852_09 |
| **RNA_profile11-vs-MIR_profile15** | Unigene016619 | novel-m0370-5p | -0.632455532 | 0.43,0.27,1.585,0.575 | 0.0100,7.0715,0.0100,1.2745 | Unigene016619_01 |
| **RNA_profile11-vs-MIR_profile15** | Unigene090564 | novel-m0650-5p | -0.632455532 | 6.39,2.815,24.665,9.51 | 0.0100,15.3331,0.0100,1.6715 | Unigene090564_03 |
| **RNA_profile11-vs-MIR_profile15** | Unigene028077 | novel-m0247-5p | -0.6 | 19.955,18.29,41.005,18.03 | 53.1721,229.8227,75.7104,169.0999 | Unigene028077_01 |
| **RNA_profile13-vs-MIR_profile3** | Unigene075279 | novel-m0244-3p | -0.632455532 | 1.69,7.39,1.53,0.805 | 2.8015,0.0100,0.0100,5.2374 | Unigene075279_01 |
| **RNA_profile13-vs-MIR_profile3** | Unigene075279 | novel-m0269-3p | -0.632455532 | 1.69,7.39,1.53,0.805 | 2.8015,0.0100,0.0100,5.2374 | Unigene075279_01 |
| **RNA_profile13-vs-MIR_profile3** | Unigene018655 | novel-m0206-5p | -0.632455532 | 5.235,10.355,3.085,2.345 | 0.9243,0.0100,0.0100,6.0707 | Unigene018655_02 |
| **RNA_profile13-vs-MIR_profile3** | Unigene086774 | MIR1134-z | -0.737864787 | 0.625,1.195,0.72,0.435 | 2.8015,0.0100,0.0100,1.6715 | Unigene086774_01 |
| **RNA_profile13-vs-MIR_profile3** | Unigene087362 | novel-m0540-3p | -0.632455532 | 0.45,0.765,0.335,0.28 | 3.2612,0.0100,0.0100,4.2056 | Unigene087362_01 |
| **RNA_profile13-vs-MIR_profile3** | Unigene059621 | MIR1134-z | -0.737864787 | 9.46,25.335,14.52,7.38 | 2.8015,0.0100,0.0100,1.6715 | Unigene059621_02 |
| **RNA_profile13-vs-MIR_profile3** | Unigene030893 | novel-m0244-3p | -0.632455532 | 0.925,8.98,0.2,0.085 | 2.8015,0.0100,0.0100,5.2374 | Unigene030893_01 |
| **RNA_profile13-vs-MIR_profile3** | Unigene030893 | novel-m0269-3p | -0.632455532 | 0.925,8.98,0.2,0.085 | 2.8015,0.0100,0.0100,5.2374 | Unigene030893_01 |
| **RNA_profile13-vs-MIR_profile5** | Unigene086498 | novel-m0402-5p | -0.948683298 | 0.02,4.665,0.02,0.000500 | 18.7721,0.0100,32.4678,61.5378 | Unigene086498_01 |
| **RNA_profile13-vs-MIR_profile5** | Unigene091103 | novel-m0412-5p | -0.8 | 6.485,8.19,5.875,3.215 | 138.3795,40.1530,413.5979,327.0849 | Unigene091103_02 |
| **RNA_profile13-vs-MIR_profile5** | Unigene075279 | novel-m0259-3p | -1 | 1.69,7.39,1.53,0.805 | 2.6338,0.0100,4.7341,10.2762 | Unigene075279_01 |
| **RNA_profile13-vs-MIR_profile5** | Unigene075279 | novel-m0307-3p | -1 | 1.69,7.39,1.53,0.805 | 2.6338,0.0100,4.7341,10.2762 | Unigene075279_01 |
| **RNA_profile13-vs-MIR_profile5** | Unigene053882 | novel-m0039-3p | -0.8 | 20.12,34.7,23.465,13.91 | 2.6338,0.0100,4.0629,16.9817 | Unigene053882_06 |
| **RNA_profile13-vs-MIR_profile5** | Unigene088801 | novel-m0105-3p | -0.8 | 2.045,4.425,1.115,0.65 | 3.2612,0.0100,2.8970,4.4041 | Unigene088801_01 |
| **RNA_profile13-vs-MIR_profile5** | Unigene088801 | novel-m0325-5p | -0.8 | 2.045,4.425,1.115,0.65 | 3.2612,0.0100,2.8970,4.4041 | Unigene088801_01 |
| **RNA_profile13-vs-MIR_profile5** | Unigene088801 | novel-m0382-3p | -0.8 | 2.045,4.425,1.115,0.65 | 3.2612,0.0100,2.8970,4.4041 | Unigene088801_01 |
| **RNA_profile13-vs-MIR_profile5** | Unigene088801 | novel-m0558-3p | -0.8 | 2.045,4.425,1.115,0.65 | 3.2612,0.0100,2.8970,4.4041 | Unigene088801_01 |
| **RNA_profile13-vs-MIR_profile5** | Unigene047538 | novel-m0338-3p | -1 | 10.605,17.925,6.805,5.52 | 2.5147,0.0100,3.5329,7.1418 | Unigene047538_01 |
| **RNA_profile13-vs-MIR_profile5** | Unigene033976 | novel-m0494-3p | -1 | 0.995,4.1,1.1,0.52 | 5.6031,0.0100,1.1356,7.5387 | Unigene033976_01 |
| **RNA_profile13-vs-MIR_profile5** | Unigene080385 | MIR8175-y | -0.8 | 1.615,3.085,2.33,0.65 | 3.5531,0.0100,5.0926,6.7054 | Unigene080385_04 |
| **RNA_profile13-vs-MIR_profile5** | Unigene056442 | novel-m0123-5p | -1 | 1.98,3.085,1.38,1.27 | 0.9243,0.0100,1.2062,4.6026 | Unigene056442_02 |
| **RNA_profile13-vs-MIR_profile5** | Unigene056442 | novel-m0395-5p | -1 | 1.98,3.085,1.38,1.27 | 0.9243,0.0100,1.2062,4.6026 | Unigene056442_02 |
| **RNA_profile13-vs-MIR_profile5** | Unigene056442 | novel-m0027-5p | -1 | 1.98,3.085,1.38,1.27 | 0.9243,0.0100,1.2062,4.6026 | Unigene056442_02 |
| **RNA_profile13-vs-MIR_profile5** | Unigene056442 | novel-m0593-5p | -1 | 1.98,3.085,1.38,1.27 | 0.9243,0.0100,1.2062,4.6026 | Unigene056442_02 |
| **RNA_profile13-vs-MIR_profile5** | Unigene065708 | novel-m0340-3p | -1 | 1.045,2.1,1.245,0.8 | 1.8872,0.0100,1.1356,7.5387 | Unigene065708_01 |
| **RNA_profile13-vs-MIR_profile5** | Unigene030893 | novel-m0123-5p | -1 | 0.925,8.98,0.2,0.085 | 0.9243,0.0100,1.2062,4.6026 | Unigene030893_01 |
| **RNA_profile13-vs-MIR_profile5** | Unigene030893 | novel-m0395-5p | -1 | 0.925,8.98,0.2,0.085 | 0.9243,0.0100,1.2062,4.6026 | Unigene030893_01 |
| **RNA_profile13-vs-MIR_profile5** | Unigene030893 | novel-m0027-5p | -1 | 0.925,8.98,0.2,0.085 | 0.9243,0.0100,1.2062,4.6026 | Unigene030893_01 |
| **RNA_profile13-vs-MIR_profile5** | Unigene030893 | novel-m0593-5p | -1 | 0.925,8.98,0.2,0.085 | 0.9243,0.0100,1.2062,4.6026 | Unigene030893_01 |
| **RNA_profile13-vs-MIR_profile6** | Unigene021536 | MIR395-y | -0.8 | 4.785,15.59,5.395,2.72 | 263.0202,93.0495,300.1204,886.3774 | Unigene021536_03 |
| **RNA_profile13-vs-MIR_profile6** | Unigene055972 | novel-m0390-5p | -1 | 10.255,16.575,13.985,6.62 | 36.8799,10.6421,34.3407,60.1896 | Unigene055972_01 |
| **RNA_profile13-vs-MIR_profile6** | Unigene074163 | novel-m0445-3p | -0.8 | 2.33,4.13,2.665,1.74 | 25.6300,7.1462,44.6565,71.3385 | Unigene074163_01 |
| **RNA_profile13-vs-MIR_profile6** | Unigene074163 | novel-m0444-3p | -0.8 | 2.33,4.13,2.665,1.74 | 25.6300,7.1462,44.6565,71.3385 | Unigene074163_01 |
| **RNA_profile13-vs-MIR_profile6** | Unigene074162 | novel-m0445-3p | -1 | 7.28,12.745,7.23,4.505 | 25.6300,7.1462,44.6565,71.3385 | Unigene074162_02 |
| **RNA_profile13-vs-MIR_profile6** | Unigene074162 | novel-m0444-3p | -1 | 7.28,12.745,7.23,4.505 | 25.6300,7.1462,44.6565,71.3385 | Unigene074162_02 |
| **RNA_profile13-vs-MIR_profile6** | Unigene060731 | novel-m0445-3p | -0.8 | 8.725,15.335,9.105,7.015 | 25.6300,7.1462,44.6565,71.3385 | Unigene060731_01 |
| **RNA_profile13-vs-MIR_profile6** | Unigene060731 | novel-m0444-3p | -0.8 | 8.725,15.335,9.105,7.015 | 25.6300,7.1462,44.6565,71.3385 | Unigene060731_01 |
| **RNA_profile13-vs-MIR_profile12** | Unigene060731 | MIR845-x | -0.8 | 8.725,15.335,9.105,7.015 | 99445.7811,72438.8438,167614.6491,169189.4274 | Unigene060731_01 |
| **RNA_profile13-vs-MIR_profile12** | Unigene069963 | novel-m0505-3p | -0.948683298 | 1.405,2.245,1.245,0.89 | 0.0100,0.0100,1.7008,2.9361 | Unigene069963_03 |
| **RNA_profile13-vs-MIR_profile12** | Unigene074163 | MIR845-x | -0.8 | 2.33,4.13,2.665,1.74 | 99445.7811,72438.8438,167614.6491,169189.4274 | Unigene074163_01 |
| **RNA_profile13-vs-MIR_profile12** | Unigene074162 | MIR845-x | -1 | 7.28,12.745,7.23,4.505 | 99445.7811,72438.8438,167614.6491,169189.4274 | Unigene074162_02 |
| **RNA_profile13-vs-MIR_profile12** | Unigene016083 | novel-m0492-5p | -0.948683298 | 2.89,9.585,1.995,1.25 | 0.0100,0.0100,5.8293,6.3085 | Unigene016083_01 |
| **RNA_profile13-vs-MIR_profile12** | Unigene016083 | novel-m0323-3p | -0.948683298 | 2.89,9.585,1.995,1.25 | 0.0100,0.0100,5.8293,6.3085 | Unigene016083_01 |
| **RNA_profile13-vs-MIR_profile12** | Unigene016083 | novel-m0623-3p | -0.948683298 | 2.89,9.585,1.995,1.25 | 0.0100,0.0100,5.8293,6.3085 | Unigene016083_01 |
| **RNA_profile13-vs-MIR_profile12** | Unigene016083 | novel-m0432-3p | -0.948683298 | 2.89,9.585,1.995,1.25 | 0.0100,0.0100,5.8293,6.3085 | Unigene016083_01 |
| **RNA_profile13-vs-MIR_profile19** | Unigene045641 | novel-m0174-5p | -0.8 | 4.635,9.67,4.395,3.285 | 2052.2731,2606.8575,3522.1640,4735.6087 | Unigene045641_01 |
| **RNA_profile14-vs-MIR_profile4** | Unigene034367 | MIR1863-y | -0.544331054 | 0.000500,4.82,0.000500,0.000500 | 3.0935,0.0100,1.7008,0.0100 | Unigene034367_01 |
| **RNA_profile14-vs-MIR_profile4** | Unigene077916 | MIR1863-y | -0.544331054 | 0.000500,2.665,0.000500,0.000500 | 3.0935,0.0100,1.7008,0.0100 | Unigene077916_01 |
| **RNA_profile14-vs-MIR_profile4** | Unigene076553 | MIR393-y | -0.632455532 | 5.835,12.96,7.62,6.775 | 5.3917,0.0100,2.4074,0.0100 | Unigene076553_04 |
| **RNA_profile14-vs-MIR_profile4** | Unigene064481 | MIR1863-y | -0.737864787 | 0.755,6.07,0.225,1.1 | 3.0935,0.0100,1.7008,0.0100 | Unigene064481_01 |
| **RNA_profile14-vs-MIR_profile4** | Unigene034701 | MIR1871-y | -0.737864787 | 1.17,2.81,1.265,1.315 | 1.8436,0.0100,2.3317,0.0100 | Unigene034701_01 |
| **RNA_profile14-vs-MIR_profile4** | Unigene042505 | MIR1863-y | -0.632455532 | 0.195,4.335,0.54,0.285 | 3.0935,0.0100,1.7008,0.0100 | Unigene042505_05 |
| **RNA_profile14-vs-MIR_profile4** | Unigene068063 | novel-m0457-3p | -0.948683298 | 0.39,26.11,0.185,1.84 | 2.7629,0.0100,3.4623,0.0100 | Unigene068063_01 |
| **RNA_profile14-vs-MIR_profile4** | Unigene038130 | MIR1871-y | -0.948683298 | 0.695,3.735,0.615,0.95 | 1.8436,0.0100,2.3317,0.0100 | Unigene038130_01 |
| **RNA_profile14-vs-MIR_profile4** | Unigene038130 | MIR1863-y | -0.737864787 | 0.695,3.735,0.615,0.95 | 3.0935,0.0100,1.7008,0.0100 | Unigene038130_01 |
| **RNA_profile14-vs-MIR_profile4** | Unigene046928 | MIR1863-y | -0.632455532 | 0.25,1.84,0.39,0.35 | 3.0935,0.0100,1.7008,0.0100 | Unigene046928_03 |
| **RNA_profile14-vs-MIR_profile4** | Unigene072283 | novel-m0457-3p | -0.737864787 | 3.82,9.545,5.29,5.645 | 2.7629,0.0100,3.4623,0.0100 | Unigene072283_04 |
| **RNA_profile14-vs-MIR_profile4** | Unigene051306 | novel-m0337-5p | -0.544331054 | 0.000500,1.325,0.000500,0.000500 | 0.9243,0.0100,2.3317,0.0100 | Unigene051306_01 |
| **RNA_profile14-vs-MIR_profile4** | Unigene094100 | MIR1863-y | -0.544331054 | 0.000500,2.62,0.000500,0.000500 | 3.0935,0.0100,1.7008,0.0100 | Unigene094100_01 |
| **RNA_profile14-vs-MIR_profile4** | Unigene066151 | MIR1863-y | -0.544331054 | 0.000500,3.015,0.000500,0.000500 | 3.0935,0.0100,1.7008,0.0100 | Unigene066151_01 |
| **RNA_profile14-vs-MIR_profile4** | Unigene077025 | MIR1863-y | -0.544331054 | 0.000500,2.09,0.000500,0.000500 | 3.0935,0.0100,1.7008,0.0100 | Unigene077025_01 |
| **RNA_profile14-vs-MIR_profile4** | Unigene031506 | MIR1863-y | -0.737864787 | 2.4,5.945,2.08,2.535 | 3.0935,0.0100,1.7008,0.0100 | Unigene031506_04 |
| **RNA_profile14-vs-MIR_profile4** | Unigene035415 | novel-m0457-3p | -0.948683298 | 0.84,5.055,0.825,1.005 | 2.7629,0.0100,3.4623,0.0100 | Unigene035415_01 |
| **RNA_profile15-vs-MIR_profile4** | Unigene083418 | novel-m0023-5p | -0.737864787 | 0.33,0.915,0.24,0.85 | 2.6338,0.0100,2.2662,0.0100 | Unigene083418_01 |
| **RNA_profile15-vs-MIR_profile4** | Unigene023857 | MIR1863-y | -0.948683298 | 5.13,10.655,5.895,7.71 | 3.0935,0.0100,1.7008,0.0100 | Unigene023857_01 |
| **RNA_profile15-vs-MIR_profile4** | Unigene035419 | novel-m0457-3p | -0.888888889 | 0.000500,13.805,0.000500,0.625 | 2.7629,0.0100,3.4623,0.0100 | Unigene035419_01 |
| **RNA_profile15-vs-MIR_profile4** | Unigene034700 | MIR1863-y | -0.948683298 | 9.16,24.485,9.965,15.435 | 3.0935,0.0100,1.7008,0.0100 | Unigene034700_08 |
| **RNA_profile15-vs-MIR_profile4** | Unigene066880 | novel-m0457-3p | -0.948683298 | 5.285,23.95,2.83,12.245 | 2.7629,0.0100,3.4623,0.0100 | Unigene066880_01 |
| **RNA_profile15-vs-MIR_profile4** | Unigene000913 | MIR1863-y | -0.888888889 | 0.000500,1.025,0.000500,0.05 | 3.0935,0.0100,1.7008,0.0100 | Unigene000913_01 |
| **RNA_profile15-vs-MIR_profile4** | Unigene061444 | MIR1871-y | -0.948683298 | 1.145,2.225,1,1.635 | 1.8436,0.0100,2.3317,0.0100 | Unigene061444_03 |
| **RNA_profile15-vs-MIR_profile4** | Unigene061444 | MIR1863-y | -0.737864787 | 1.145,2.225,1,1.635 | 3.0935,0.0100,1.7008,0.0100 | Unigene061444_03 |
| **RNA_profile15-vs-MIR_profile4** | Unigene051098 | novel-m0457-3p | -0.737864787 | 1,59.62,3.175,11.69 | 2.7629,0.0100,3.4623,0.0100 | Unigene051098_01 |
| **RNA_profile15-vs-MIR_profile4** | Unigene095128 | novel-m0339-3p | -0.737864787 | 0.755,7,0.865,3.175 | 4.8079,0.0100,5.4103,0.0100 | Unigene095128_02 |
| **RNA_profile15-vs-MIR_profile4** | Unigene083882 | novel-m0577-5p | -0.737864787 | 3.875,9.18,3.205,5.55 | 6.8142,0.0100,1.8067,0.0100 | Unigene083882_03 |
| **RNA_profile15-vs-MIR_profile4** | Unigene025994 | novel-m0457-3p | -0.948683298 | 0.515,7.25,0.25,1.51 | 2.7629,0.0100,3.4623,0.0100 | Unigene025994_02 |
| **RNA_profile16-vs-MIR_profile3** | Unigene031929 | novel-m0540-3p | -0.737864787 | 0.13,0.42,1.045,0.16 | 3.2612,0.0100,0.0100,4.2056 | Unigene031929_01 |
| **RNA_profile16-vs-MIR_profile3** | Unigene046849 | MIR1134-z | -0.737864787 | 3.01,5.245,6.875,2.47 | 2.8015,0.0100,0.0100,1.6715 | Unigene046849_06 |
| **RNA_profile16-vs-MIR_profile3** | Unigene081729 | MIR1134-z | -0.948683298 | 0.42,0.995,1.315,0.425 | 2.8015,0.0100,0.0100,1.6715 | Unigene081729_01 |
| **RNA_profile16-vs-MIR_profile3** | Unigene059108 | novel-m0151-5p | -0.737864787 | 10.67,26.025,21.21,14.06 | 0.9243,0.0100,0.0100,5.0389 | Unigene059108_05 |
| **RNA_profile16-vs-MIR_profile3** | Unigene005663 | MIR1134-z | -0.737864787 | 1.1,2.21,2.745,0.71 | 2.8015,0.0100,0.0100,1.6715 | Unigene005663_07 |
| **RNA_profile16-vs-MIR_profile3** | Unigene071611 | MIR1134-z | -0.737864787 | 3.38,6.02,8.79,1.575 | 2.8015,0.0100,0.0100,1.6715 | Unigene071611_01 |
| **RNA_profile16-vs-MIR_profile3** | Unigene045833 | novel-m0244-3p | -0.737864787 | 0.09,0.425,0.785,0.095 | 2.8015,0.0100,0.0100,5.2374 | Unigene045833_01 |
| **RNA_profile16-vs-MIR_profile3** | Unigene045833 | novel-m0269-3p | -0.737864787 | 0.09,0.425,0.785,0.095 | 2.8015,0.0100,0.0100,5.2374 | Unigene045833_01 |
| **RNA_profile16-vs-MIR_profile3** | Unigene064372 | MIR1134-z | -0.948683298 | 2.21,4.845,4.67,3.28 | 2.8015,0.0100,0.0100,1.6715 | Unigene064372_03 |
| **RNA_profile16-vs-MIR_profile3** | Unigene073400 | novel-m0244-3p | -0.737864787 | 18.86,41.23,28.69,20.305 | 2.8015,0.0100,0.0100,5.2374 | Unigene073400_02 |
| **RNA_profile16-vs-MIR_profile3** | Unigene073400 | novel-m0269-3p | -0.737864787 | 18.86,41.23,28.69,20.305 | 2.8015,0.0100,0.0100,5.2374 | Unigene073400_02 |
| **RNA_profile16-vs-MIR_profile3** | Unigene071773 | MIR1134-z | -0.948683298 | 0.27,2.35,10.225,0.505 | 2.8015,0.0100,0.0100,1.6715 | Unigene071773_04 |
| **RNA_profile16-vs-MIR_profile3** | Unigene062480 | MIR1134-z | -0.737864787 | 1.47,4.455,2.875,1.1 | 2.8015,0.0100,0.0100,1.6715 | Unigene062480_01 |
| **RNA_profile16-vs-MIR_profile3** | Unigene044092 | MIR1134-z | -0.948683298 | 0.31,2.615,1.195,0.49 | 2.8015,0.0100,0.0100,1.6715 | Unigene044092_02 |
| **RNA_profile16-vs-MIR_profile3** | Unigene089443 | MIR1134-z | -0.888888889 | 0.000500,0.35,0.475,0.000500 | 2.8015,0.0100,0.0100,1.6715 | Unigene089443_01 |
| **RNA_profile16-vs-MIR_profile3** | Unigene079462 | MIR1134-z | -0.948683298 | 0.13,0.48,1.245,0.185 | 2.8015,0.0100,0.0100,1.6715 | Unigene079462_01 |
| **RNA_profile16-vs-MIR_profile3** | Unigene006614 | MIR1134-z | -0.948683298 | 0.425,3.13,1.925,0.53 | 2.8015,0.0100,0.0100,1.6715 | Unigene006614_01 |
| **RNA_profile16-vs-MIR_profile3** | Unigene006718 | MIR1134-z | -0.948683298 | 0.12,0.875,1.625,0.13 | 2.8015,0.0100,0.0100,1.6715 | Unigene006718_01 |
| **RNA_profile16-vs-MIR_profile3** | Unigene043478 | MIR1134-z | -0.948683298 | 0.31,1.685,1.68,0.63 | 2.8015,0.0100,0.0100,1.6715 | Unigene043478_01 |
| **RNA_profile17-vs-MIR_profile3** | Unigene088442 | MIR1134-z | -0.632455532 | 0.000500,1.245,0.06,0.09 | 2.8015,0.0100,0.0100,1.6715 | Unigene088442_01 |
| **RNA_profile17-vs-MIR_profile3** | Unigene058790 | novel-m0451-3p | -0.737864787 | 0.000500,2.66,6.375,0.965 | 3.0935,0.0100,0.0100,5.0389 | Unigene058790_01 |
| **RNA_profile17-vs-MIR_profile3** | Unigene058790 | novel-m0450-3p | -0.737864787 | 0.000500,2.66,6.375,0.965 | 3.0935,0.0100,0.0100,5.0389 | Unigene058790_01 |
| **RNA_profile17-vs-MIR_profile3** | Unigene055254 | novel-m0348-3p | -0.948683298 | 0.000500,0.77,2.5,0.51 | 5.0998,0.0100,0.0100,1.9093 | Unigene055254_01 |
| **RNA_profile17-vs-MIR_profile3** | Unigene016869 | MIR1134-z | -0.948683298 | 0.000500,0.625,0.425,0.115 | 2.8015,0.0100,0.0100,1.6715 | Unigene016869_01 |
| **RNA_profile17-vs-MIR_profile3** | Unigene000336 | novel-m0003-3p | -0.737864787 | 0.000500,0.975,4.335,0.165 | 3.1421,0.0100,0.0100,6.7054 | Unigene000336_01 |
| **RNA_profile17-vs-MIR_profile3** | Unigene060588 | MIR1134-z | -0.632455532 | 0.000500,1.52,0.1,0.205 | 2.8015,0.0100,0.0100,1.6715 | Unigene060588_02 |
| **RNA_profile17-vs-MIR_profile3** | Unigene017867 | MIR1134-z | -0.632455532 | 1.45,3.175,2.125,2.44 | 2.8015,0.0100,0.0100,1.6715 | Unigene017867_02 |
| **RNA_profile17-vs-MIR_profile3** | Unigene000338 | novel-m0003-3p | -0.737864787 | 0.22,2.935,5.315,1.76 | 3.1421,0.0100,0.0100,6.7054 | Unigene000338_01 |
| **RNA_profile18-vs-MIR_profile3** | Unigene034123 | MIR1134-z | -0.948683298 | 2.34,4.43,6.94,4.255 | 2.8015,0.0100,0.0100,1.6715 | Unigene034123_01 |
| **RNA_profile18-vs-MIR_profile3** | Unigene050253 | novel-m0450-3p | -0.737864787 | 2.835,5.575,14.475,4.43 | 3.0935,0.0100,0.0100,5.0389 | Unigene050253_11 |
| **RNA_profile18-vs-MIR_profile3** | Unigene050253 | novel-m0451-3p | -0.737864787 | 2.835,5.575,14.475,4.43 | 3.0935,0.0100,0.0100,5.0389 | Unigene050253_11 |
| **RNA_profile18-vs-MIR_profile3** | Unigene068490 | MIR1134-z | -0.632455532 | 0.14,0.355,1.63,0.365 | 2.8015,0.0100,0.0100,1.6715 | Unigene068490_02 |
| **RNA_profile18-vs-MIR_profile3** | Unigene027450 | MIR1134-z | -0.948683298 | 0.245,0.68,1.925,0.325 | 2.8015,0.0100,0.0100,1.6715 | Unigene027450_01 |
| **RNA_profile18-vs-MIR_profile3** | Unigene030023 | MIR1134-z | -0.948683298 | 0.12,0.75,2.07,0.45 | 2.8015,0.0100,0.0100,1.6715 | Unigene030023_01 |
| **RNA_profile18-vs-MIR_profile3** | Unigene090058 | novel-m0244-3p | -0.737864787 | 2.25,3.46,5.13,2.715 | 2.8015,0.0100,0.0100,5.2374 | Unigene090058_01 |
| **RNA_profile18-vs-MIR_profile3** | Unigene090058 | novel-m0269-3p | -0.737864787 | 2.25,3.46,5.13,2.715 | 2.8015,0.0100,0.0100,5.2374 | Unigene090058_01 |
| **RNA_profile18-vs-MIR_profile3** | Unigene047675 | novel-m0333-5p | -0.632455532 | 2.195,3.28,5.16,3.715 | 4.8079,0.0100,0.0100,2.9361 | Unigene047675_01 |
| **RNA_profile18-vs-MIR_profile3** | Unigene051906 | MIR1134-z | -0.948683298 | 0.045,0.59,1.835,0.325 | 2.8015,0.0100,0.0100,1.6715 | Unigene051906_01 |
| **RNA_profile18-vs-MIR_profile3** | Unigene016926 | novel-m0540-3p | -0.737864787 | 0.03,0.345,1.01,0.185 | 3.2612,0.0100,0.0100,4.2056 | Unigene016926_01 |
| **RNA_profile18-vs-MIR_profile3** | Unigene084172 | MIR1134-z | -0.948683298 | 0.055,0.34,1.175,0.215 | 2.8015,0.0100,0.0100,1.6715 | Unigene084172_01 |
| **RNA_profile18-vs-MIR_profile3** | Unigene000342 | novel-m0003-3p | -0.737864787 | 0.035,0.36,1.455,0.215 | 3.1421,0.0100,0.0100,6.7054 | Unigene000342_01 |
| **RNA_profile18-vs-MIR_profile3** | Unigene000335 | novel-m0003-3p | -0.737864787 | 0.17,0.76,1.725,0.34 | 3.1421,0.0100,0.0100,6.7054 | Unigene000335_01 |
| **RNA_profile18-vs-MIR_profile3** | Unigene092551 | MIR1134-z | -0.948683298 | 2.825,7.63,10.91,4.79 | 2.8015,0.0100,0.0100,1.6715 | Unigene092551_01 |
| **RNA_profile18-vs-MIR_profile3** | Unigene054145 | MIR1134-z | -0.948683298 | 0.15,0.555,1.535,0.28 | 2.8015,0.0100,0.0100,1.6715 | Unigene054145_01 |
| **RNA_profile18-vs-MIR_profile3** | Unigene058390 | MIR1134-z | -0.948683298 | 0.095,0.17,0.645,0.165 | 2.8015,0.0100,0.0100,1.6715 | Unigene058390_01 |
| **RNA_profile18-vs-MIR_profile3** | Unigene085380 | MIR1134-z | -0.948683298 | 0.81,1.25,3.335,1.235 | 2.8015,0.0100,0.0100,1.6715 | Unigene085380_02 |
| **RNA_profile18-vs-MIR_profile3** | Unigene000236 | MIR1134-z | -0.948683298 | 1.995,8.125,32.385,4.48 | 2.8015,0.0100,0.0100,1.6715 | Unigene000236_01 |
| **RNA_profile18-vs-MIR_profile3** | Unigene028719 | MIR1134-z | -0.948683298 | 0.3,0.79,1.665,0.435 | 2.8015,0.0100,0.0100,1.6715 | Unigene028719_01 |
| **RNA_profile18-vs-MIR_profile3** | Unigene000341 | novel-m0003-3p | -0.737864787 | 0.26,1.01,3.08,0.695 | 3.1421,0.0100,0.0100,6.7054 | Unigene000341_01 |
| **RNA_profile18-vs-MIR_profile3** | Unigene055501 | MIR1134-z | -0.632455532 | 0.24,0.57,1.635,0.765 | 2.8015,0.0100,0.0100,1.6715 | Unigene055501_01 |
| **RNA_profile18-vs-MIR_profile3** | Unigene034525 | MIR1134-z | -0.632455532 | 0.11,0.335,1.53,0.42 | 2.8015,0.0100,0.0100,1.6715 | Unigene034525_01 |
| **RNA_profile18-vs-MIR_profile3** | Unigene085700 | MIR1134-z | -0.948683298 | 0.12,0.365,0.755,0.195 | 2.8015,0.0100,0.0100,1.6715 | Unigene085700_01 |
