# Supplementary material for: MicroRNAome Profile of Euphorbia kansui in Response to Methyl Jasmonate
Source: Int J Mol Sci. 2019 Mar 13;20(6):1267. doi: 10.3390/ijms20061267 (PMC6471261; doi:10.3390/ijms20061267)
Supplement: Supplementary file 1 [file ijms-20-01267-s001.zip › ijms-432617-SI-final/Table S6.doc]

**Additional File6 Table S6: KEGG pathway enrichment of pairwise comparison**

| **KEGG_A_class** | **KEGG_B_class** | **Pathway** | **CK_vs_T1 (318)** | **All**  **(12228)** | **Pvalue** | **Pathway ID** |
| --- | --- | --- | --- | --- | --- | --- |
| **Metabolism** | Metabolism of terpenoids and polyketides | Sesquiterpenoid and triterpenoid biosynthesis | 6 | 51 | 0.001984 | ko00909 |
| **Metabolism** | Lipid metabolism | Ether lipid metabolism | 6 | 54 | 0.002666 | ko00565 |
| **Metabolism** | Carbohydrate metabolism | Fructose and mannose metabolism | 9 | 123 | 0.004783 | ko00051 |
| **Metabolism** | Global and Overview | Carbon metabolism | 35 | 891 | 0.009316 | ko01200 |
| **Metabolism** | Carbohydrate metabolism | Glycolysis / Gluconeogenesis | 16 | 351 | 0.021322 | ko00010 |
| **Metabolism** | Lipid metabolism | Steroid biosynthesis | 6 | 88 | 0.026984 | ko00100 |
| **Metabolism** | Metabolism of other amino acids | Glutathione metabolism | 12 | 254 | 0.033882 | ko00480 |
| **Metabolism** | Glycan biosynthesis and metabolism | Glycosphingolipid biosynthesis - ganglio series | 2 | 13 | 0.043529 | ko00604 |
| **Metabolism** | Carbohydrate metabolism | Pentose phosphate pathway | 8 | 155 | 0.04949 | ko00030 |
| **KEGG_A_class** | **KEGG_B_class** | **Pathway** | **CK_vs_T2 (174)** | **All**  **(12228)** | **Pvalue** | **Pathway ID** |
| **Metabolism** | Global and Overview | Carbon metabolism | 27 | 891 | 0.000145 | ko01200 |
| **Metabolism** | Metabolism of other amino acids | Glutathione metabolism | 11 | 254 | 0.001021 | ko00480 |
| **Metabolism** | Carbohydrate metabolism | Glycolysis / Gluconeogenesis | 13 | 351 | 0.00153 | ko00010 |
| **Metabolism** | Energy metabolism | Carbon fixation in photosynthetic organisms | 10 | 243 | 0.002502 | ko00710 |
| **Metabolism** | Carbohydrate metabolism | Inositol phosphate metabolism | 7 | 143 | 0.004331 | ko00562 |
| **Metabolism** | Metabolism of other amino acids | beta-Alanine metabolism | 7 | 147 | 0.005032 | ko00410 |
| **Metabolism** | Carbohydrate metabolism | Pentose phosphate pathway | 7 | 155 | 0.006688 | ko00030 |
| **Metabolism** | Global and Overview | Biosynthesis of amino acids | 17 | 638 | 0.009453 | ko01230 |
| **Metabolism** | Lipid metabolism | Glycerolipid metabolism | 6 | 157 | 0.024722 | ko00561 |
| **Metabolism** | Amino acid metabolism | Tryptophan metabolism | 4 | 81 | 0.028253 | ko00380 |
| **Metabolism** | Amino acid metabolism | Tyrosine metabolism | 4 | 81 | 0.028253 | ko00350 |
| **Metabolism** | Carbohydrate metabolism | Fructose and mannose metabolism | 5 | 123 | 0.031069 | ko00051 |
| **Metabolism** | Global and Overview | Degradation of aromatic compounds | 2 | 21 | 0.035415 | ko01220 |
| **Metabolism** | Carbohydrate metabolism | Citrate cycle (TCA cycle) | 8 | 265 | 0.035788 | ko00020 |
| **Cellular Processes** | Transport and catabolism | Peroxisome | 8 | 269 | 0.038558 | ko04146 |
| **Metabolism** | Amino acid metabolism | Lysine biosynthesis | 2 | 22 | 0.038599 | ko00300 |
| **Metabolism** | Lipid metabolism | Ether lipid metabolism | 3 | 54 | 0.041404 | ko00565 |
| **Metabolism** | Metabolism of terpenoids and polyketides | Sesquiterpenoid and triterpenoid biosynthesis | 1 | 51 | 0.519262 | ko00909 |
| **Metabolism** | Lipid metabolism | Steroid biosynthesis | 2 | 88 | 0.357108 | ko00100 |
| **Genetic Information Processing** | Replication and repair | Homologous recombination | 5 | 134 | 0.042475 | ko03440 |
| **KEGG_A_class** | **KEGG_B_class** | **Pathway** | **CK_vs_T3 (156)** | **All**  **(12228)** | **Pvalue** | **Pathway ID** |
| **Metabolism** | Metabolism of terpenoids and polyketides | Sesquiterpenoid and triterpenoid biosynthesis | 4 | 51 | 0.003998 | ko00909 |
| **Metabolism** | Lipid metabolism | Steroid biosynthesis | 5 | 88 | 0.005308 | ko00100 |
| **Environmental Information Processing** | Membrane transport | ABC transporters | 4 | 69 | 0.011598 | ko02010 |
| **Metabolism** | Energy metabolism | Carbon fixation in photosynthetic organisms | 8 | 243 | 0.012687 | ko00710 |
| **Metabolism** | Lipid metabolism | Glycerolipid metabolism | 6 | 157 | 0.015301 | ko00561 |
| **Genetic Information Processing** | Transcription | RNA polymerase | 5 | 135 | 0.029285 | ko03020 |
| **Genetic Information Processing** | Translation | Aminoacyl-tRNA biosynthesis | 7 | 243 | 0.036192 | ko00970 |
| **Metabolism** | Amino acid metabolism | Glycine, serine and threonine metabolism | 5 | 156 | 0.049475 | ko00260 |
| **Metabolism** | Global and Overview | Carbon metabolism | 17 | 891 | 0.06186 | ko01200 |
| **Metabolism** | Metabolism of other amino acids | Glutathione metabolism | 6 | 254 | 0.106579 | ko00480 |
| **Metabolism** | Lipid metabolism | Ether lipid metabolism | 2 | 54 | 0.15101 | ko00565 |
| **Environmental Information Processing** | Signal transduction | Plant hormone signal transduction | 8 | 316 | 0.049527 | ko04075 |
| **KEGG_A_class** | **KEGG_B_class** | **Pathway** | **T1_vs_T2 (218)** | **All**  **(12228)** | **Pvalue** | **Pathway ID** |
| **Metabolism** | Energy metabolism | Photosynthesis | 8 | 115 | 0.001036 | ko00195 |
| **Metabolism** | Lipid metabolism | Ether lipid metabolism | 5 | 54 | 0.002675 | ko00565 |
| **Metabolism** | Biosynthesis of other secondary metabolites | Tropane, piperidine and pyridine alkaloid biosynthesis | 4 | 59 | 0.020843 | ko00960 |
| **Metabolism** | Metabolism of terpenoids and polyketides | Sesquiterpenoid and triterpenoid biosynthesis | 2 | 51 | 0.230499 | ko00909 |
| **Metabolism** | Metabolism of other amino acids | Glutathione metabolism | 6 | 254 | 0.300277 | ko00480 |
| **Metabolism** | Lipid metabolism | Steroid biosynthesis | 1 | 88 | 0.795814 | ko00100 |
| **Metabolism** | Global and Overview | Carbon metabolism | 13 | 891 | 0.811463 | ko01200 |
| **Metabolism** | Lipid metabolism | Glycerophospholipid metabolism | 8 | 197 | 0.024926 | ko00564 |
| **KEGG_A_class** | **KEGG_B_class** | **Pathway** | **T1_vs_T3 (262)** | **All**  **(12228)** | **Pvalue** | **Pathway ID** |
| **Metabolism** | Lipid metabolism | Ether lipid metabolism | 6 | 54 | 0.000997 | ko00565 |
| **Metabolism** | Amino acid metabolism | Glycine, serine and threonine metabolism | 10 | 156 | 0.001944 | ko00260 |
| **Metabolism** | Energy metabolism | Carbon fixation in photosynthetic organisms | 12 | 243 | 0.006186 | ko00710 |
| **Metabolism** | Lipid metabolism | Glycerolipid metabolism | 9 | 157 | 0.006697 | ko00561 |
| **Metabolism** | Metabolism of other amino acids | Glutathione metabolism | 12 | 254 | 0.008673 | ko00480 |
| **Metabolism** | Global and Overview | Carbon metabolism | 29 | 891 | 0.015715 | ko01200 |
| **Metabolism** | Carbohydrate metabolism | Fructose and mannose metabolism | 7 | 123 | 0.016587 | ko00051 |
| **Metabolism** | Carbohydrate metabolism | Pentose and glucuronate interconversions | 7 | 137 | 0.028014 | ko00040 |
| **Metabolism** | Glycan biosynthesis and metabolism | Glycosphingolipid biosynthesis - ganglio series | 2 | 13 | 0.030532 | ko00604 |
| **Metabolism** | Carbohydrate metabolism | Glyoxylate and dicarboxylate metabolism | 11 | 274 | 0.034121 | ko00630 |
| **Metabolism** | Energy metabolism | Photosynthesis | 6 | 115 | 0.037145 | ko00195 |
| **Metabolism** | Biosynthesis of other secondary metabolites | Tropane, piperidine and pyridine alkaloid biosynthesis | 4 | 59 | 0.037422 | ko00960 |
| **Metabolism** | Metabolism of terpenoids and polyketides | Sesquiterpenoid and triterpenoid biosynthesis | 3 | 51 | 0.095752 | ko00909 |
| **Metabolism** | Lipid metabolism | Steroid biosynthesis | 3 | 88 | 0.291886 | ko00100 |
| **Metabolism** | Metabolism of cofactors and vitamins | Pantothenate and CoA biosynthesis | 5 | 92 | 0.047507 | ko00770 |
| **KEGG_A_class** | **KEGG_B_class** | **Pathway** | **T2_vs_T3 (125)** | **All**  **(12228)** | **Pvalue** | **Pathway ID** |
| **Metabolism** | Metabolism of other amino acids | Glutathione metabolism | 7 | 254 | 0.015415 | ko00480 |
| **Metabolism** | Lipid metabolism | Ether lipid metabolism | 3 | 54 | 0.017713 | ko00565 |
| **Metabolism** | Amino acid metabolism | Glycine, serine and threonine metabolism | 5 | 156 | 0.021892 | ko00260 |
| **Metabolism** | Lipid metabolism | Glycerolipid metabolism | 5 | 157 | 0.022433 | ko00561 |
| **Metabolism** | Glycan biosynthesis and metabolism | Glycosylphosphatidylinositol(GPI)-anchor biosynthesis | 3 | 71 | 0.036049 | ko00563 |
| **Metabolism** | Global and Overview | Carbon metabolism | 15 | 891 | 0.038077 | ko01200 |
| **Metabolism** | Metabolism of cofactors and vitamins | Thiamine metabolism | 2 | 31 | 0.039747 | ko00730 |
| **Metabolism** | Global and Overview | 2-Oxocarboxylic acid metabolism | 5 | 192 | 0.047052 | ko01210 |
| **Metabolism** | Lipid metabolism | Steroid biosynthesis | 3 | 88 | 0.061197 | ko00100 |
| **Metabolism** | Metabolism of terpenoids and polyketides | Sesquiterpenoid and triterpenoid biosynthesis | 2 | 51 | 0.09566 | ko00909 |
