# Supplementary material for: MicroRNAome Profile of Euphorbia kansui in Response to Methyl Jasmonate
Source: Int J Mol Sci. 2019 Mar 13;20(6):1267. doi: 10.3390/ijms20061267 (PMC6471261; doi:10.3390/ijms20061267)
Supplement: Supplementary file 1 [file ijms-20-01267-s001.zip › ijms-432617-SI-final/Table S7.doc]

**Additional file7 Table S7: GO enrichment of all miRNA target DEGs, the number and functional class affected by MeJA**

| **CK-vs-T1** | | **CK-vs-T2** | | **CK-vs-T3** | | **T1-vs-T2** | | **T1-vs-T3** | | **T2-vs-T3** | |
| --- | --- | --- | --- | --- | --- | --- | --- | --- | --- | --- | --- |
| **Class** | **number** | **Class** | **number** | **Class** | **number** | **Class** | **numbe** | **Class** | **numbe** | **Class** | **numbe** |
| single-organism process | 195 | detoxification | 4 | detoxification | 4 | cellular process | 215 | cellular process | 248 | localization | 33 |
| cellular process | 282 | biological regulation | 23 | single-organism process | 90 | response to stimulus | 52 | single-organism process | 169 | single-organism process | 80 |
| biological regulation | 43 | single-organism process | 101 | immune system process | 2 | biological regulation | 36 | response to stimulus | 56 | immune system process | 2 |
| response to stimulus | 63 | cellular process | 148 | biological regulation | 20 | single-organism process | 146 | signaling | 15 | multicellular organismal process | 12 |
| detoxification | 5 | reproduction | 10 | multi-organism process | 6 | signaling | 14 | biological regulation | 37 | biological regulation | 15 |
| signaling | 17 | response to stimulus | 31 | cellular process | 129 | multi-organism process | 11 | localization | 56 | multi-organism process | 4 |
| reproduction | 18 | reproductive process | 9 | reproduction | 9 | detoxification | 4 | detoxification | 4 | signaling | 5 |
| reproductive process | 17 | multicellular organismal process | 13 | multicellular organismal process | 13 | rhythmic process | 2 | immune system process | 2 | developmental process | 12 |
| rhythmic process | 2 | metabolic process | 157 | reproductive process | 8 | developmental process | 26 | reproductive process | 14 | detoxification | 1 |
| developmental process | 32 | developmental process | 16 | signaling | 6 | multicellular organismal process | 20 | reproduction | 14 | cellular process | 107 |
| growth | 3 | multi-organism process | 5 | localization | 27 | reproductive process | 13 | developmental process | 27 | reproductive process | 5 |
| multi-organism process | 9 | immune system process | 1 | metabolic process | 138 | growth | 3 | multi-organism process | 8 | reproduction | 5 |
| localization | 55 | signaling | 6 | response to stimulus | 25 | reproduction | 13 | rhythmic process | 1 | cellular component organization or biogenesis | 12 |
| multicellular organismal process | 22 | growth | 1 | developmental process | 14 | localization | 42 | multicellular organismal process | 19 | response to stimulus | 19 |
| immune system process | 1 | localization | 27 | growth | 1 | metabolic process | 216 | metabolic process | 249 | metabolic process | 116 |
| metabolic process | 281 | cellular component organization or biogenesis | 15 | cellular component organization or biogenesis | 13 | immune system process | 1 | growth | 2 | molecular transducer activity | 2 |
| cellular component organization or biogenesis | 28 | catalytic activity | 170 | catalytic activity | 154 | cellular component organization or biogenesis | 18 | cellular component organization or biogenesis | 23 | transporter activity | 17 |
| catalytic activity | 305 | molecular transducer activity | 1 | molecular transducer activity | 1 | electron carrier activity | 3 | electron carrier activity | 2 | signal transducer activity | 2 |
| electron carrier activity | 2 | structural molecule activity | 7 | antioxidant activity | 1 | catalytic activity | 221 | transporter activity | 31 | electron carrier activity | 1 |
| binding | 232 | antioxidant activity | 1 | signal transducer activity | 1 | molecular transducer activity | 1 | catalytic activity | 263 | catalytic activity | 125 |
| transporter activity | 28 | signal transducer activity | 1 | binding | 102 | binding | 163 | binding | 197 | molecular function regulator | 1 |
| molecular transducer activity | 1 | molecular function regulator | 2 | molecular function regulator | 1 | transporter activity | 20 | molecular transducer activity | 1 | nucleic acid binding transcription factor activity | 1 |
| molecular function regulator | 3 | binding | 119 | nucleic acid binding transcription factor activity | 1 | signal transducer activity | 1 | molecular function regulator | 3 | binding | 86 |
| antioxidant activity | 1 | transporter activity | 13 | transporter activity | 10 | molecular function regulator | 1 | signal transducer activity | 1 | structural molecule activity | 3 |
| signal transducer activity | 1 | nucleic acid binding transcription factor activity | 1 | structural molecule activity | 1 | nucleic acid binding transcription factor activity | 1 | nucleic acid binding transcription factor activity | 1 | membrane part | 70 |
| nucleic acid binding transcription factor activity | 2 | membrane | 112 | membrane part | 83 | structural molecule activity | 2 | structural molecule activity | 2 | membrane | 84 |
| structural molecule activity | 6 | membrane part | 81 | membrane | 104 | membrane part | 119 | membrane part | 152 | organelle | 56 |
| membrane part | 160 | extracellular region | 3 | virion | 2 | membrane | 152 | membrane | 193 | membrane-enclosed lumen | 3 |
| membrane | 206 | virion | 1 | virion part | 2 | cell junction | 12 | nucleoid | 1 | organelle part | 20 |
| cell junction | 14 | virion part | 1 | membrane-enclosed lumen | 5 | organelle | 120 | cell junction | 11 | extracellular region | 1 |
| virion | 2 | membrane-enclosed lumen | 5 | organelle | 74 | virion | 1 | extracellular region | 4 | cell junction | 2 |
| virion part | 2 | organelle | 81 | cell junction | 5 | virion part | 1 | virion | 1 | cell | 73 |
| organelle | 148 | cell junction | 3 | extracellular region | 2 | extracellular region | 3 | virion part | 1 | cell part | 73 |
| extracellular region | 3 | organelle part | 25 | organelle part | 23 | cell | 155 | membrane-enclosed lumen | 5 | macromolecular complex | 10 |
| membrane-enclosed lumen | 5 | cell | 105 | cell | 94 | cell part | 155 | organelle | 123 |  |  |
| organelle part | 43 | cell part | 105 | cell part | 94 | organelle part | 39 | organelle part | 37 |  |  |
| cell | 190 | macromolecular complex | 15 | macromolecular complex | 14 | macromolecular complex | 31 | macromolecular complex | 28 |  |  |
| cell part | 190 |  |  |  |  | membrane-enclosed lumen | 3 | cell | 166 |  |  |
| macromolecular complex | 30 |  |  |  |  |  |  | cell part | 166 |  |  |
