# Supplementary material for: MicroRNAome Profile of Euphorbia kansui in Response to Methyl Jasmonate
Source: Int J Mol Sci. 2019 Mar 13;20(6):1267. doi: 10.3390/ijms20061267 (PMC6471261; doi:10.3390/ijms20061267)
Supplement: Supplementary file 1 [file ijms-20-01267-s001.zip › ijms-432617-SI-final/Table S8.doc]

**Additional File8 TableS8:** Association analysis of the MeJA-responsive differential expressed miRNA and their target mRNAs in terpenoid biosynthesis.

| GeneID | Pathway | miRNA_id | CK | T1 | T2 | T3 |
| --- | --- | --- | --- | --- | --- | --- |
| Unigene002085 | - | MIR535-y | 13.33085 | 5.93785 | 10.98095 | 6.62615 |
|  |  | MIR7752-y | 0.01 | 0.01 | 0.01 | 0.01 |
| Unigene003418 | ko00900//Terpenoid backbone biosynthesis | MIR7747-x | 0.01 | 0.01 | 1.1356 | 0.01 |
|  |  | novel-m0022-5p | 15.5545 | 0.01 | 18.9716 | 9.24455 |
| Unigene001452 |  | MIR5563-x |  |  |  |  |
| Unigene023256 | ko00900//Terpenoid backbone biosynthesis | novel-m0252-5p | 6.31095 | 1.87842 | 8.267 | 1.6715 |
|  |  | novel-m0323-3p | 0.01 | 0.01 | 5.82935 | 6.3085 |
|  |  | novel-m0432-3p | 0.01 | 0.01 | 5.82935 | 6.3085 |
|  |  | novel-m0491-5p | 6.31095 | 4.69605 | 8.267 | 1.16715 |
|  |  | novel-m0492-5p | 0.01 | 0.01 | 5.82935 | 6.3085 |
|  |  | novel-m0623-3p | 0.01 | 0.01 | 5.82935 | 6.3085 |
| Unigene049665 | ko00900//Terpenoid backbone biosynthesis | MIR5139-x | 6.7706 | 0.01 | 6.9952 | 2.93605 |
|  |  | MIR8155-y | 4.932 | 0.01 | 3.4623 | 2.93605 |
| Unigene055774 | ko00900//Terpenoid backbone biosynthesis | novel-m0389-5p | 1.8436 | 0.01 | 4.0629 | 5.87215 |
| Unigene011218 | ko00130//Ubiquinone and other terpenoid-quinone biosynthesis | novel-m0364-5p | 0.01 | 0.01 | 1.80675 | 3.76935 |
|  |  | novel-m0370-5p | 0.01 | 11.099 | 0.01 | 1.27455 |
| Unigene062800 | ko00130//Ubiquinone and other terpenoid-quinone biosynthesis | MIR1134-z | 2.80155 | 0.01 | 0.01 | 1.6715 |
| Unigene062801 | ko00130//Ubiquinone and other terpenoid-quinone biosynthesis | MIR1134-z | 2.80155 | 0.01 | 0.01 | 1.6715 |
| Unigene045546 | ko00940//Phenylpropanoid biosynthesis;ko00130//Ubiquinone and other terpenoid-quinone | novel-m0324-3p | 1.38395 | 0.01 | 0.01 | 4.40415 |
| Unigene028155 | ko00130//Ubiquinone and other terpenoid-quinone biosynthesis;ko00360//Phenylalanine metabolism;ko00350//Tyrosine metabolism | novel-m0580-5p | 0.9243 | 3.5756 | 0.01 | 7.7765 |
|  |  | novel-m0581-5p | 0.9243 | 3.5756 | 0.01 | 7.7765 |
| Unigene056630 | ko00130//Ubiquinone and other terpenoid-quinone biosynthesis | MIR846-x | 0.01 | 0.01 | 0.01 | 0.01 |
| Unigene063000 | ko00130//Ubiquinone and other terpenoid-quinone biosynthesis | MIR1446-x | 17.4735 | 15.4029 | 26.8856 | 19.283 |
| Unigene069994 | ko00130//Ubiquinone and other terpenoid-quinone biosynthesis | MIR394-y | 0.01 | 0.01 | 1.80675 | 0.01 |
|  |  | novel-m0496-5p | 0.9243 | 2.3854 | 1.13555 | 1.17455 |
| Unigene070519 | ko00130//Ubiquinone and other terpenoid-quinone biosynthesis | MIR1531-y | 0.01 | 0.01 | 0.01 | 0.01 |
| Unigene031929 | ko00100//Steroid biosynthesis;ko00909//Sesquiterpenoid and triterpenoid biosynthesis | novel-m0540-3p | 3.2612 | 0.01 | 0.01 | 4.2056 |
| Unigene045324 | ko00100//Steroid biosynthesis;ko00909//Sesquiterpenoid and triterpenoid biosynthesis | MIR8565-x | 60.5771 | 0.01 | 69.80935 | 24.16275 |
| Unigene049481 | ko00100//Steroid biosynthesis | MIR2275-x | 0.01 | 0.01 | 1.13555 | 0.01 |
|  |  | novel-m0041-5p | 2.6338 | 0.01 | 1.20615 | 0.01 |
| Unigene057348 | ko00100//Steroid biosynthesis | MIR6034-y | 17.89645 | 18.90375 | 20.42035 | 17.61645 |
|  |  | novel-m0527-5p | 2.80155 | 26.9824 | 5.75875 | 14.0456 |
| Unigene073014 | ko00100//Steroid biosynthesis;ko00909//Sesquiterpenoid and triterpenoid biosynthesis | MIR8175-y | 3.5531 | 0.01 | 5.09255 | 6.7054 |
|  |  | novel-m0568-3p | 0.01 | 0.01 | 0.01 | 4.2056 |
| Unigene076528 | ko00100//Steroid biosynthesis;ko00909//Sesquiterpenoid and triterpenoid biosynthesis | MIR1134-z | 2.80155 | 0.01 | 0.01 | 1.6715 |
|  |  | novel-m0168-5p | 11.79 | 12.95275 | 6.46525 | 6.9039 |
|  |  | novel-m0225-5p | 0.9243 | 3.5756 | 4.0982 | 0.01 |
|  |  | novel-m0595-3p | 18.4802 | 65.0077 | 36.21255 | 99.46905 |
| Unigene020790 | ko00100//Steroid biosynthesis | MIR159-y | 679167.15 | 575220 | 588603.1 | 561846.55 |
|  |  | MIR319-y | 18905.04 | 5770.8115 | 7038.94 | 5088.303 |
| Unigene003865 | ko00909//Sesquiterpenoid and triterpenoid biosynthesis | MIR1863-y | 3.09345 | 0.01 | 1.70085 | 0.01 |
|  |  | novel-m0277-3p | 26.46195 | 18.9386 | 24.27095 | 10.08235 |
| Unigene006820 | ko00909//Sesquiterpenoid and triterpenoid biosynthesis | MIR535-z | 8.4047 | 0.01 | 9.85695 | 6.74475 |
| Unigene069286 | ko00909//Sesquiterpenoid and triterpenoid biosynthesis | novel-m0330-3p | 0.01 | 9.417 | 1.80675 | 1.90935 |
| Unigene045324 | ko00100//Steroid biosynthesis;ko00909//Sesquiterpenoid and triterpenoid biosynthesis | MIR8565-x | 60.5771 | 0.01 | 69.80935 | 24.16275 |
| Unigene018448 | ko00904//Diterpenoid biosynthesis | novel-m0170-5p | 1.38395 | 11.76255 | 0.01 | 0.01 |
|  |  | novel-m0171-5p | 1.38395 | 11.76255 | 0.01 | 0.01 |
| Unigene050717 | ko00100//Steroid biosynthesis | novel-m0346-3p | 13.02245 | 13.0225 | 21.6215 | 14.08495 |
| Unigene014530 | ko00100//Steroid biosynthesis | MIR845-y | 334.38635 | 94.6929 | 319.27305 | 205.4057 |
| Unigene068133 | ko00904//Diterpenoid biosynthesis | novel-m0574-5p | 0.9243 | 68.4737 | 1.70085 | 0.01 |
| Unigene019800 | ko00902//Monoterpenoid biosynthesis | novel-m0077-5p | 4.1418 | 4.7658 | 3.0079 | 1.6715 |
|  |  | novel-m0078-5p | 4.1418 | 4.7658 | 3.0079 | 1.6715 |
|  |  | novel-m0152-3p | 2.6338 | 4.7658 | 6.5005 | 0.001 |
|  |  | novel-m0187-3p | 16.43015 | 35.4271 | 20.06685 | 14.87885 |
|  |  | novel-m0243-3p | 0.9243 | 0.01 | 2.83145 | 0.01 |
| Unigene022256 | ko01200//Carbon metabolism;ko00620//Pyruvate metabolism;ko00630//Glyoxylate and dicarboxylate metabolism; ko01212//Fatty acid metabolism; ko00280//Valine, leucine and isoleucine degradation; ko00071//Fatty acid degradation; ko00900//Terpenoid backbone biosynthesis;ko00640//Propanoate metabolism; ko00310//Lysine degradation; ko00380//Tryptophan metabolism; ko00650//Butanoate metabolism; ko00072//Synthesis and degradation of ketone bodies | novel-m0077-5p | 4.1418 | 4.7658 | 3.0079 | 1.6715 |
|  |  | novel-m0078-5p | 4.1418 | 4.7658 | 3.0079 | 1.6715 |
| Unigene031929 | ko00564//Glycerophospholipid metabolism;  ko00561//Glycerolipid metabolism;  ko00592//alpha-Linolenic acid metabolism;  ko00100//Steroid biosynthesis;  ko00565//Ether lipid metabolism;  ko00590//Arachidonic acid metabolism | novel-m0540-3p | 3.2612 | 0.01 | 0.01 | 4.2056 |
| Unigene043985 | - | novel-m0253-3p | 3.6822 | 2.3854 | 8.65575 | 12.1413 |

Notes: CK, T1, T2 and T3 represent 0 h, 24 h, 36 h and 48 h MeJA treatment respectively.
