# Supplementary material for: MicroRNAome Profile of Euphorbia kansui in Response to Methyl Jasmonate
Source: Int J Mol Sci. 2019 Mar 13;20(6):1267. doi: 10.3390/ijms20061267 (PMC6471261; doi:10.3390/ijms20061267)
Supplement: Supplementary file 1 [file ijms-20-01267-s001.zip › ijms-432617-SI-final/Table S9.doc]

**Additional File9 TableS9:** The result of QPCR verification

|  | STD | | | | | | | |
| --- | --- | --- | --- | --- | --- | --- | --- | --- |
|  | CK | | T1 | | T2 | | T3 | |
|  | CK1 | CK2 | T1-1 | T1-2 | T2-1 | T2-2 | T3-1 | T3-2 |
| MIR1446-x | 0.4715857 | 0.426207 | 0.0179025 | 0.0345878 | 0.0575789 | 0.04175169 | 0.7574533 | 0.7355770 |
| MIR535-y | 0.0444604 | 0.0518261 | 0.0322835 | 0.0654143 | 0.1967068 | 0.1901807 | 1.8588134 | 0.1254472 |
| MIR845-y | 0.0245475 | 0.0392291 | 0.0153618 | 0.0359837 | 0.4060742 | 0.9226855 | 1.2149693 | 0.0892671 |
| MIR394-y | 0.0250921 | 0.063865 | 0.0441647 | 0.0964773 | 0.5181444 | 0.3419843 | 1.2439538 | 0.2340613 |
| MIR5563-x | 0.1099073 | 0.1529219 | 0.0539067 | 0.021878 | 0.4288173 | 1.1195108 | 1.4581357 | 0.3556644 |
| novel-m0022-5p | 0.0152867 | 0.0358023 | 0.0845545 | 0.0856367 | 0.7174839 | 0.609571 | 0.6710789 | 0.285491 |
| novel-m0346-3p | 0.0682596 | 0.2359 | 0.0644203 | 0.0267096 | 0.2427357 | 0.1286846 | 0.273306 | 0.0634375 |

Notes: CK, T1, T2 and T3 represent 0 h, 24 h, 36 h and 48 h MeJA treatment respectively (two biological replicates for each treatment, named CK-1, CK-2, T1-1, T1-2, T2-1, T2-2, T3-1 and T3-2).
